# Ethyl Pinacol Boronates as Advantageous Precursors for Copper-Mediated Radiofluorination

**DOI:** 10.1021/acs.orglett.5c02055

**Published:** 2025-06-10

**Authors:** Nikolaos Hadjipaschalis, Sebastiano Ortalli, Zijun Chen, Robert S. Paton, Joseph Ford, Matthew Tredwell, Véronique Gouverneur

**Affiliations:** † Chemistry Research Laboratory, 6396 University of Oxford, 12 Mansfield Road, Oxford OX1 3TA, United Kingdom; ¶ Department of Chemistry, 224023 Colorado State University, Fort Collins, Colorado 80528, United States; ‡ Wales Research and Diagnostic PET Imaging Centre, Cardiff University, University Hospital of Wales, Heath Park, Cardiff, CF14 4XN, United Kingdom; § School of Chemistry, Cardiff University, Main Building, Park Place, Cardiff, CF10 3AT, United Kingdom

## Abstract

(Hetero)­aryl pinacol boronic esters are routinely used for ^18^F-labeling and have accelerated numerous diagnostic and drug discovery programs. An analysis of the current state-of-play, however, highlights a pending challenge. Reports have indicated that some pinacol boronic esters are unstable and difficult to purify, hindering broader adoption in the clinic. Herein, we demonstrate that more stable boronic esters derived from 3,4-diethylhexane-3,4-diol (Epin) are highly suitable for copper-mediated radiolabeling. Impact is illustrated with the automated synthesis of [^18^F]­FMZ.

## Graphical abstract

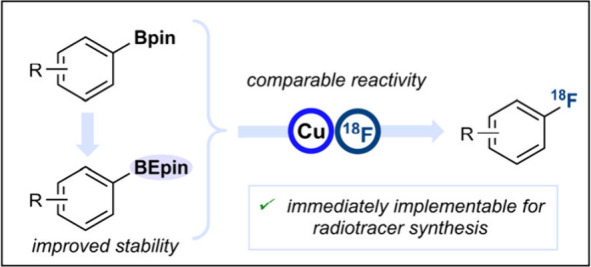

(Hetero)­aryl boron reagents are versatile intermediates for chemical synthesis and are utilized in many common reactions, such as the prominent Suzuki–Miyaura cross-coupling.^1^ It is therefore unsurprising that these substrates have also made substantial impact in radiosynthesis.^2^ Numerous deboronative methodologies have been developed that enable efficient radiolabeling with various radionuclides,^3^ most notably fluorine-18 (*t*_1/2_ = 109.8 min, 97% β^+^, *E*_max_(β^+^) = 635 keV), which is widely used in positron emission tomography (PET) imaging.^2,4^

First disclosed by our group in 2014, the copper-mediated radiofluorination of boron reagents was successfully implemented with readily available (hetero)­aryl pinacol boronic ester (Bpin) precursors (Scheme 1A).^5^ Since then, this technology has enabled access to a wide range of complex (hetero)­aryl ^18^F-labeled radiotracers.^6^ The combined efforts of many groups, including our own, have led to the identification of superior solvents, copper mediators and beneficial additives.^6b,6c,7^ Particular attention has been given to the optimization of reagents used during the elution and drying of [^18^F]­fluoride, due to base sensitivity under full-batch conditions.^8^ Automation on commercial radiosynthesizers has also been demonstrated, and detailed protocols for the preparation of clinical radiotracers compliant with current Good Manufacturing Practice (cGMP), e.g. [^18^F]­FDOPA and [^18^F]­flumazenil ([^18^F]­FMZ), have been disclosed.^6d–6f,9^ Together, this represents a wealth of knowledge that practitioners can readily exploit when applying this labeling technology to prepare ^18^F-labeled radiotracers.

**Scheme 1:**
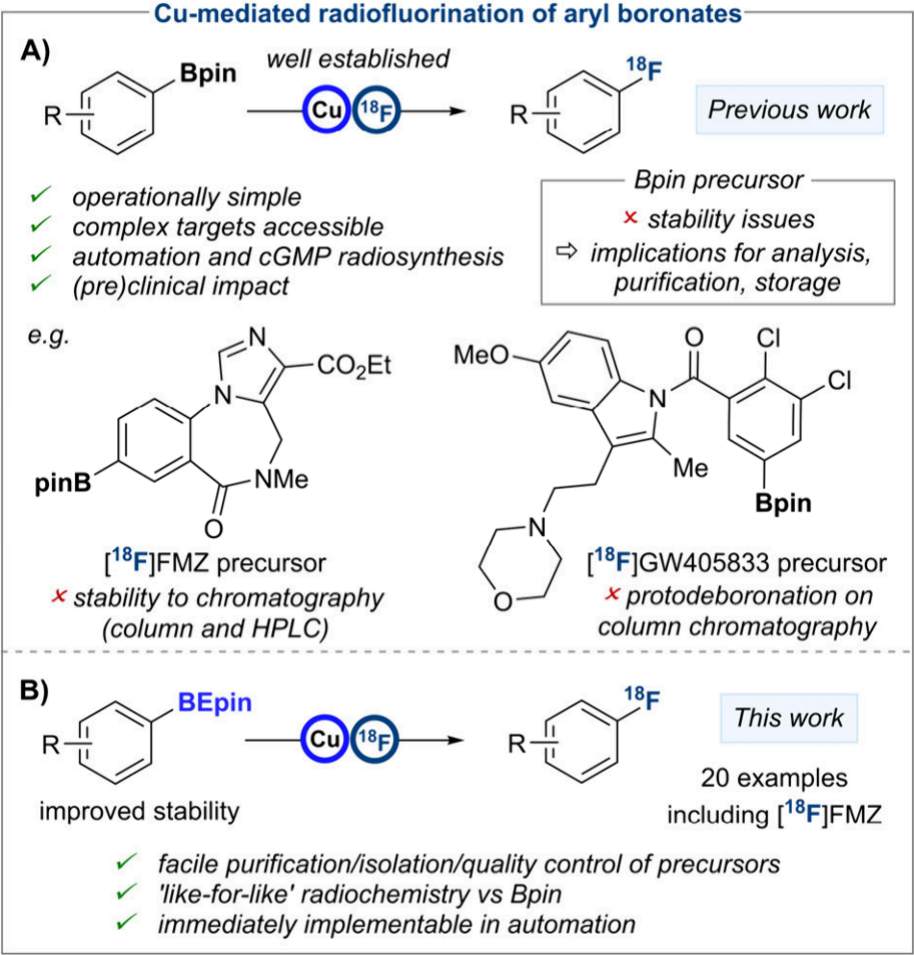
Copper-Mediated Radiofluorination of (Hetero)­aryl Boron Reagents: A) Advantages and Pending Challenges of Hetero­(aryl)-Bpin Precursors; B) ^18^F-Radiolabeling of More Stable (Hetero)­aryl-BEpins (This Work)

a pin = pinacol, Epin = 3,4-diethylhexane-3,4-diol.

Despite these advantages, a pending challenge has been identified for this methodology. The isolation and purification of some (hetero)­aryl-Bpin substrates can be challenging, with streaking, overadsorption or even degradation reported during silica gel chromatography.^10^ Such challenges were encountered in the isolation of the Bpin precursors for [^18^F]­FMZ and an ^18^F-labeled analogue of GW405833.^6b,11^ Scott, Sanford and co-workers also reported that a [^11^C]­LY2795050-Bpin precursor hydrolyzed on storage.^12^ Maurer and co-workers reported instability of an [^18^F]­olaparib-Bpin precursor on storage above −20 °C, or under reverse-phase high-performance liquid chromatography (RP-HPLC) purification.^6h^ This is undesirable as such hydrolysis during RP-HPLC complicates quality control of Bpin precursors for cGMP-compliant radiosynthesis and hinders adoption in the clinic.^13^

Scott, Sanford and co-workers reported an elegant solution with a tandem Ir-catalyzed C–H borylation/Cu-mediated radiofluorination protocol that bypasses the need to isolate or store unstable Bpin precursors.^11^ However, this strategy may be challenging to generalize as regioselectivity issues are observed for some arenes. We opted instead for a ‘like-for-like’ replacement of (hetero)­aryl-Bpin precursors with a superior boronic ester class that addresses these instability challenges yet is immediately applicable in well-established radiosynthesis protocols.

In our search for a new precursor class, we selected (hetero)­aryl boronic 1,1,2,2-tetraethylethylene glycol ester (BEpin) reagents, reported by Ikawa and co-workers for use in Suzuki–Miyaura reactions, but yet to be adopted in radiochemistry.^14^ These boron reagents were immediately appealing as radiofluorination substrates, due to their structural and electronic similarity to existing Bpin precursors. Also, they can be prepared analogously using commercial reagents. Crucially, in contrast to boronic acid or Bpin substrates, they exhibit greater stability to protodeboronation and are easily purified by conventional silica gel chromatography.^14^ The enhanced stability of (hetero)­aryl-BEpins is proposed to arise from the ability of the pendant ethyl groups to spatially protect the vacant p orbital on boron. Preliminary density functional theory (DFT) calculations corroborate this hypothesis. Conformational analysis of aryl-BEpin **1a**, derived from 4-cyanophenylboronic acid, reveals low-lying conformers that adopt structures where this steric protection is indeed possible (43% combined Boltzmann population at 298 K) (Figure S22). Herein, we demonstrate that (hetero)­aryl-BEpin precursors are amenable to copper-mediated radiofluorination. This new radiochemistry benefits from facile isolation, handling and quality control of radiotracer precursors, all advantageous characteristics for research and development, and clinical studies, alike (Scheme 1B).

We first sought to establish the reactivity of aryl-BEpin **1a** to copper-mediated radiofluorination (Scheme 2). Along with the analogous Bpin precursor **1b** as a benchmark, this was subjected to our initial set of conditions using an aliquot of a [^18^F]­KF/K_222_ solution (5–20 MBq), tetrakis­(pyridine)­copper­(II) triflate [Cu­(OTf)_2_py_4_] and DMF solvent.^5^ As in previous studies, Bpin **1b** underwent radiofluorination forming ^18^F-labeled fluoroarene [^18^F]**2** in 43% radiochemical yield (RCY), as determined by radio-HPLC analysis of the crude reaction mixture. Pleasingly, for BEpin substrate **1a**, [^18^F]**2** was formed in 58% RCY, highlighting its suitability for copper-mediated radiofluorination. For completeness, alternative aryl boron derivatives reported to exhibit increased stability, were evaluated as alternative substrates, however none proved superior to **1a** or **1b** (Scheme S2).^1a^

**Scheme 2:**
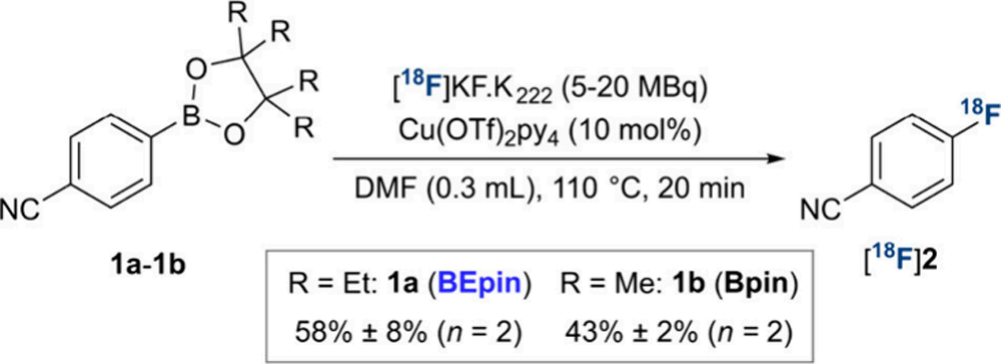
Comparison of Aryl Boronic Esters **1a** and **1b**

a
**1a** or **1b** (0.06 mmol), reaction purged with air (20 mL) before heating. Radiochemical yields (RCY) determined by radio-HPLC analysis of the crude reaction mixture.

The effect of various reaction parameters was investigated next (Table 1). DMF and DMA were similarly suitable solvents (Table 1, entries 1 and 2), while 1,3-dimethyl-2-imidazolidinone (DMI) delivered the highest RCY of [^18^F]**2** (81%) (Table 1, entry 3). Other polar aprotic solvents proved unsuitable (Table 1, entries 4–6). Increasing the loading of Cu­(OTf)_2_py_4_ from 0.1 up to 1 equiv had a beneficial effect, delivering [^18^F]**2** in 96% RCY (Table 1, entry 7). Pleasingly, a reduction in the loading of starting material from 0.06 to 0.02 mmol had no detrimental effect on RCY (Table 1, entry 8). Finally, the reaction tolerated the use of a combination of Cu­(OTf)_2_ and pyridine, rather than the preformed Cu­(OTf)_2_py_4_ complex (Table 1, entry 9).^6a^ These observations for BEpin precursors are in line with trends previously observed in the development of the reaction with (hetero)­aryl-Bpin substrates, suggesting the effect of reaction parameters is advantageously consistent for these classes of precursors.^5,6b,6c^

**Table 1:**
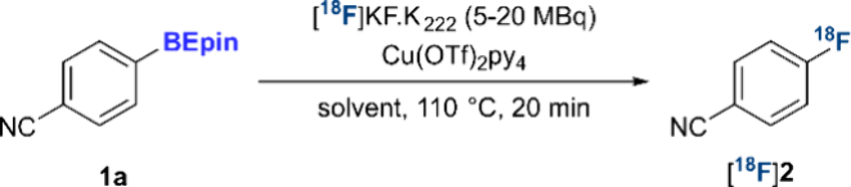
Optimization of the Copper-Mediated Radiofluorination of **1a**

**Entry**	**[Cu] loading**	**Solvent**	**RCY** ^b^
1	0.1 equiv	DMF	58% ± 8% (*n* = 2)
2	0.1 equiv	DMA	51% (*n* = 1)
3	0.1 equiv	DMI	81% (*n* = 1)
4	0.1 equiv	NMP	0% (*n* = 1)
5	0.1 equiv	DMSO	4% (*n* = 1)
6	0.1 equiv	MeCN	10% (*n* = 1)
7	1 equiv	DMI	96% (*n* = 1)
8^c^	1 equiv	DMI	95% ± 2% (*n* = 2)
9^c^ ^,^ [Table-fn t1fn4]	1 equiv	DMI	80% (*n* = 1)

Unless otherwise specified, **1a** (0.06 mmol), [Cu] = [Cu­(OTf)_2_py_4_], solvent (0.3 mL), reaction purged with air (20 mL) before heating. Reactions were conducted with aliquots of a [^18^F]­KF/K_222_ solution (5–20 MBq).

a Radiochemical yields (RCY) determined by radio-HPLC analysis of the crude reaction mixture.

b **1a** (0.02 mmol).

c Cu­(OTf)_2_ (0.02 mmol), pyridine (40 μL) added in place of [Cu­(OTf)_2_py_4_] (0.02 mmol).

We next evaluated the scope of the radiofluorination of (hetero)­aryl-BEpin reagents to identify differences in reactivity with the corresponding Bpin, if any (Scheme 3). Electronically and sterically differentiated BEpin substrates were prepared (10–92% yields), applying either esterification of the relevant boronic acid with 3,4-diethylhexane-3,4-diol (Epin) or a Miyaura-type borylation of the (hetero)­aryl halide precursor with 4,4,4′,4′,5,5,5′,5′-octaethyl-2,2′-bi­(1,3,2-dioxaborolane) (B_2_Epin_2_), both commercially available chemicals.^14^ Applying our optimized reaction conditions, biphenyl and naphthyl reagents were converted to the desired ^18^F-fluoroarenes in RCYs of up to 98% ([^18^F]**3**, [^18^F]**4**), and the reaction tolerated *ortho*-substitution ([^18^F]**5**, 95% RCY). Electron-rich substrates were successfully radiofluorinated ([^18^F]**6**: 87% RCY, [^18^F]**7**: 96% RCY). Several ^18^F-fluoroarenes bearing diverse electron-withdrawing substituents were obtained in 91–98% RCY, including trifluoromethyl ([^18^F]**8**), ethyl ester ([^18^F]**9**), nitro ([^18^F]**10**), methanesulfonyl ([^18^F]**11**) and sulfonamide ([^18^F]**12**) groups. BEpin substrates featuring heterocycles commonly encountered in medicinal chemistry were evaluated next.^15^ 2-Pyridyl-substituted ^18^F-fluoroarene [^18^F]**13** was formed in 89% and 90% RCY from the Bpin and BEpin precursors, respectively. Two regioisomers of [^18^F]­fluoroquinoline ([^18^F]**14**, [^18^F]**15**) were obtained in excellent and good RCY, respectively, and in yields comparable to the analogous Bpin. Electron-rich heteroaromatic compounds *N*-methylindole and benzoxazole underwent ^18^F-fluorination at the 5-position; in both cases, the BEpin and Bpin substrates delivered the desired labeled products [^18^F]**16** and [^18^F]**17** in excellent RCYs (83% vs 79% and 80% vs 75%, respectively).

**Scheme 3:**
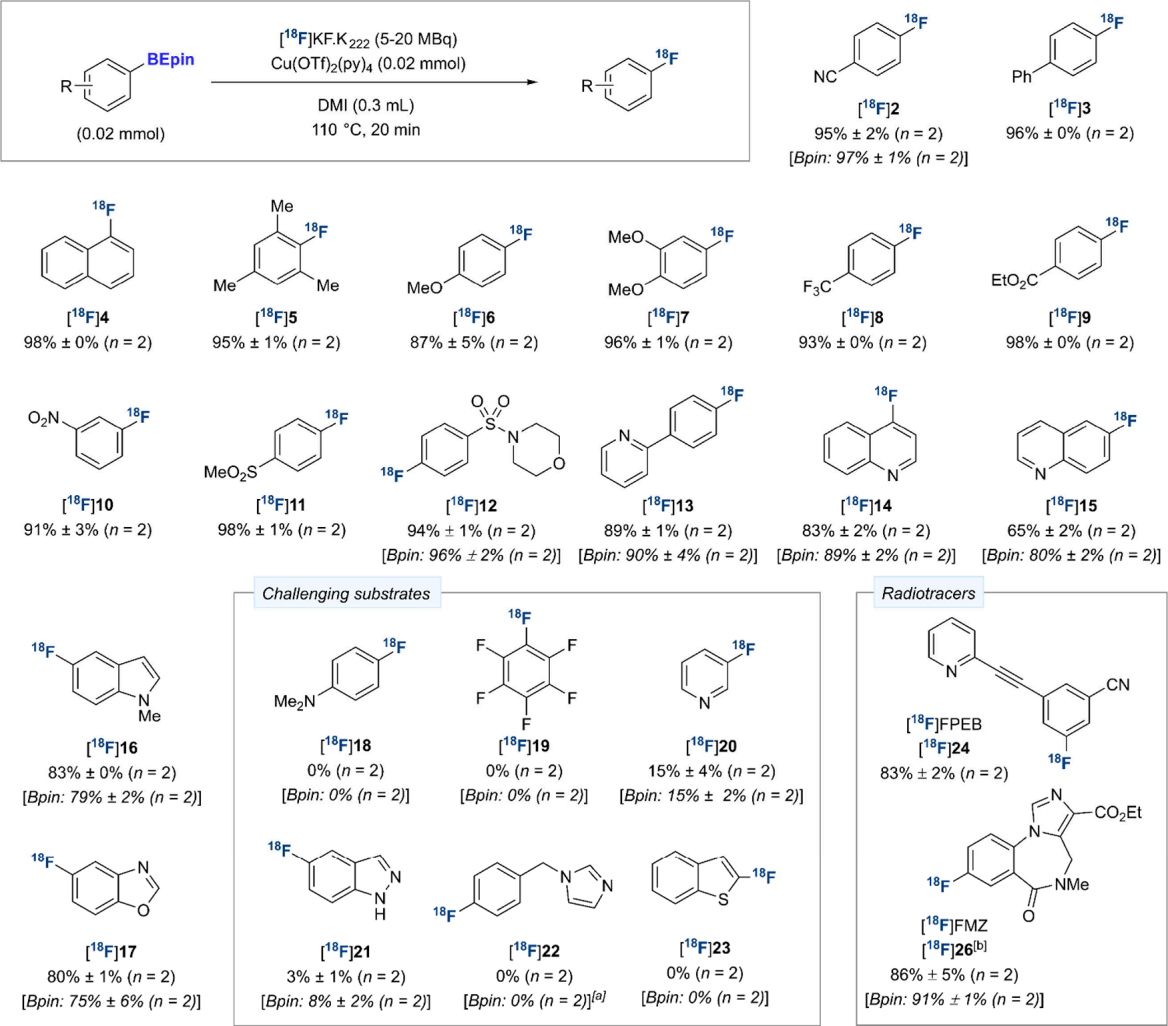
Scope of the Copper-Mediated ^18^F-Fluorination of (Hetero)­aryl-BEpin Substrates

All reactions were purged with air (20 mL) before heating. Reactions were conducted with aliquots of a [^18^F]­KF/K_222_ solution (5–20 MBq). Radiochemical yields (RCY) determined by radio-HPLC analysis of the crude reaction mixture.
a RCY from ref 6c.
b Conducted with [Cu­(OTf)_2_py_4_] (0.03 mmol).

A set of challenging substrates was next trialed to determine if the two classes of precursors display similar limitations.^6c^ A substrate featuring a dimethylamino group, as well as a per-fluorophenyl-derived substrate, were found unreactive for both the BEpin and Bpin precursors ([^18^F]**18**, [^18^F]**19**). As expected, the radiosynthesis of [^18^F]­3-fluoropyridine ([^18^F]**20**) proved challenging, with an identical, low RCY of 15% observed for the two boronates.^6c^ Boronates derived from the 5-membered heteroarenes 1*H*-indazole, 1-benzylimidazole and benzothiophene were poor substrates for copper-mediated radiofluorination ([^18^F]**21**–[^18^F]**23**).

To demonstrate applicability of these precursors to radioligand synthesis, two BEpin precursors of known ^18^F-radiotracers were prepared. [^18^F]­FPEB ([^18^F]**24**), an imaging agent for the metabotropic glutamate subtype 5 receptor, was accessed in 83% RCY.^16^ Using BEpin precursor **25**, [^18^F]­FMZ ([^18^F]**26**), a radiotracer used for imaging of γ-aminobutyric acid-A (GABA_A_) receptors, was obtained in 86% RCY.^9d,17^ As observed for other examples, **25** and its Bpin analogue displayed similar reactivity.

We next examined the use of BEpin precursors for PET radiotracer production, using [^18^F]­FMZ as a case study (Scheme 4). FMZ-BEpin **25** was prepared in 82% yield in a Pd-catalyzed borylation of an aryl bromide with B_2_Epin_2_, followed by facile purification by silica gel column chromatography. Having previously noted challenges in purifying the FMZ-Bpin precursor, a stability study was next carried out.^6b^ A known mass of the two boronates was loaded onto a silica gel column and eluted directly.^14^ While **25** was quantitatively recovered, only 70% of its Bpin analogue was eluted, suggesting overadsorption of the Bpin precursor.^10^ TLC analysis of the two samples also corroborated this, with significant streaking observed for the Bpin substrate (Figure S1).

**Scheme 4:**
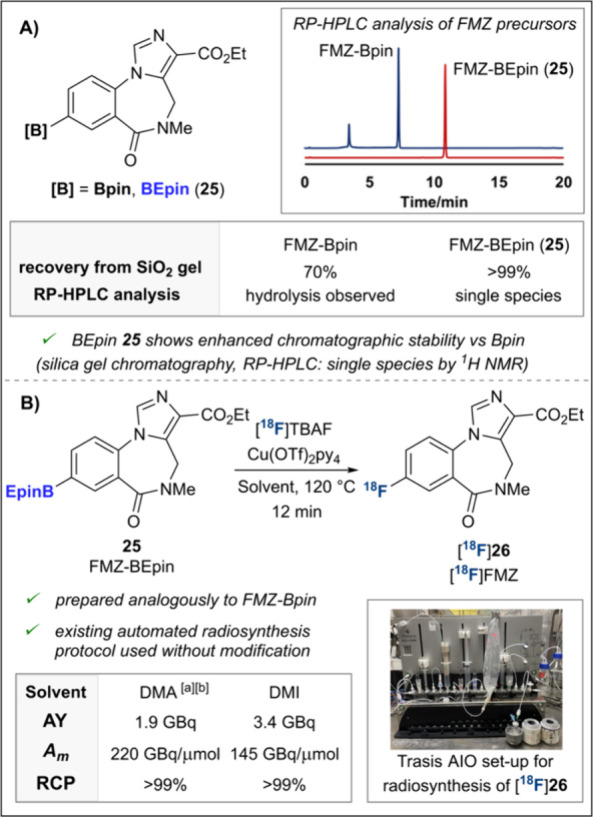
Automated Radiosynthesis of [^18^F]­FMZ: A) Stability Studies of FMZ-Boronate Precursors; B) [^18^F]­FMZ Radiosynthesis from BEpin **25** with Trasis AllinOne Radiosynthesizer

RP-HPLC and SiO_2_ gel stability test details available in Supporting Information. **25** (29 μmol), Cu­(OTf)_2_py_4_ (40 μmol), solvent (1 mL), starting activity = 25 GBq. AY = activity yield; *A*_m_ = molar activity; RCP = radiochemical purity, determined by radio-TLC and radio-HPLC.
a Original conditions (ref 9d) for the synthesis of [^18^F]**26**.
b Average over two runs.

Both boronates were then subjected to RP-HPLC analysis, commonly used for the quality control of radiotracer precursors (Scheme 4A). In an MeCN/H_2_O eluent system, the UV-HPLC trace for the [^18^F]­FMZ-Bpin precursor indicated on-column hydrolysis to the boronic acid, known for pinacol boronic esters (Figure S4).^13^ With a TFA additive (0.1% v/v, pH ∼ 2), this hydrolysis product was the only species observed, in line with findings by Lloyd-Jones and co-workers of accelerated hydrolysis of pinacol boronic esters at low pH (Figure S5).^18^ In contrast, all traces obtained for BEpin **25** featured a single sharp peak, suggesting its superior stability (Figures S2, S3). To probe this further, samples of the two boronates in MeCN-*d*_3_/D_2_O mixtures were analyzed by ^1^H NMR. For the Bpin precursor, degradation was observed when D_2_O was added, and with TFA-*d* near-complete loss of the Bpin 12H signal at 1.32 ppm occurred, with appearance of a singlet at 1.11 ppm, corresponding to free pinacol formed by hydrolysis (Figure S7). Meanwhile, **25** remained as a single stable species by ^1^H NMR, with no formation of Epin observed (Figure S6). These observations indicate that BEpin precursors can resolve quality control issues encountered with Bpin substrates as cGMP-grade radiotracer precursors.

Finally, we trialed FMZ-BEpin **25** in the cGMP-compliant automated radiosynthesis of [^18^F]­FMZ, developed by our laboratories in collaboration with Trasis for the Bpin precursor (Scheme 4B).^9d^ Using the existing sequence on a Trasis AllinOne synthesizer, with no reoptimization for BEpin **25** and an identical cassette and reagents, multipatient doses (1.9 GBq, average over two runs) of [^18^F]**26** were prepared in a form suitable for injection. Quality control was conducted based on appearance, residual amounts of copper and tetrabutylammonium salts, radiochemical purity (RCP), molar activity (*A*_m_) and pH. Furthermore, when DMI was used in place of DMA, a higher activity yield (AY) of [^18^F]**26** (3.4 GBq) was obtained.

In conclusion, this work has demonstrated the application of a novel class of (hetero)­aryl boronates in copper-mediated radiofluorination. BEpin precursors, readily prepared via established routes from commercial reagents, offer enhanced stability during silica gel chromatographic purification and analysis compared to their Bpin counterparts, while preserving reactivity when subjected to radiofluorination. The direct use of a BEpin precursor in an existing automated radiosynthesis of [^18^F]­FMZ was successfully demonstrated without the need for any modification. Given the value of copper-mediated radiofluorination strategies in (pre)­clinical imaging,^6g^ we anticipate that the advantageous characteristics of BEpin precursors will be immediately embraced by radiochemists in academia and industry.

## Supplementary Material



## Data Availability

The data underlying this study are available in the published article and its Supporting Information.

## Notes

Preparation of starting materials, substrates and reference materials; NMR data; stability tests; radiochemistry; computations; NMR spectra for novel compounds (PDF)

## References

[1] Lennox A. J. J., Lloyd-Jones G. C. (2014). Selection of boron reagents for Suzuki–Miyaura
coupling. Chem. Soc. Rev..

[2] Wilson T. C., Cailly T., Gouverneur V. (2018). Boron reagents
for divergent radiochemistry. Chem. Soc. Rev..

[3] Wilson T. C., McSweeney G., Preshlock S., Verhoog S., Tredwell M., Cailly T., Gouverneur V. (2016). Radiosynthesis of SPECT tracers via a copper mediated ^123^I iodination of (hetero)­aryl boron reagents. Chem. Commun..

[4] Ametamey S. M., Honer M., Schubiger P. A. (2008). Molecular
Imaging with PET. Chem. Rev..

[5] Tredwell M., Preshlock S. M., Taylor N. J., Gruber S., Huiban M., Passchier J., Mercier J., Génicot C., Gouverneur V. (2014). A General
Copper-Mediated Nucleophilic ^18^F Fluorination of Arenes. Angew. Chem., Int.
Ed..

[6] Mossine A. V., Brooks A. F., Makaravage K. J., Miller J. M., Ichiishi N., Sanford M. S., Scott P. J. H. (2015). Synthesis
of [^18^F]­Arenes via the Copper-Mediated [^18^F]­Fluorination
of Boronic Acids. Org. Lett..

[7] Antuganov D., Zykov M., Timofeeva K., Antuganova Y., Orlovskaya V., Krasikova R. (2017). Effect of
Pyridine Addition on the Efficiency of Copper-Mediated Radiofluorination
of Aryl Pinacol Boronates. ChemistrySelect.

[8] Zlatopolskiy B. D., Zischler J., Krapf P., Zarrad F., Urusova E. A., Kordys E., Endepols H., Neumaier B. (2015). Copper-Mediated Aromatic
Radiofluorination Revisited:
Efficient Production of PET Tracers on a Preparative Scale. Chem.—Eur. J..

[9] Mossine A. V., Brooks A. F., Bernard-Gauthier V., Bailey J. J., Ichiishi N., Schirrmacher R., Sanford M. S., Scott P. J. H. (2018). Automated synthesis of PET radiotracers
by copper-mediated ^18^F-fluorination of organoborons: Importance
of the order of addition and competing protodeborylation. J. Label. Compd. Radiopharm..

[10] Hitosugi S., Tanimoto D., Nakanishi W., Isobe H. (2012). A Facile Chromatographic
Method for Purification of Pinacol Boronic Esters. Chem. Lett..

[11] Wright J. S., Sharninghausen L. S., Preshlock S., Brooks A. F., Sanford M. S., Scott P. J. H. (2021). Sequential Ir/Cu-Mediated Method for the Meta-Selective
C–H Radiofluorination of (Hetero)­Arenes. J. Am. Chem. Soc..

[12] Kaur T., Shao X., Horikawa M., Sharninghausen L. S., Preshlock S., Brooks A. F., Henderson B. D., Koeppe R. A., DaSilva A. F., Sanford M. S., Scott P. J. H. (2023). Strategies
for the Production of [^11^C]­LY2795050 for Clinical Use. Org. Process Res. Dev..

[13] Xu J., Duran D., Mao B. (2006). On-Column
Hydrolysis Kinetics Determination of Boronic Pinacol Ester Intermediates
for Use in Optimization of Fast HPLC Methods. J. Liq. Chromatogr. Rel. Technol..

[14] Oka N., Yamada T., Sajiki H., Akai S., Ikawa T. (2022). Aryl Boronic
Esters Are Stable on Silica Gel and Reactive under Suzuki–Miyaura
Coupling Conditions. Org. Lett..

[15] Marshall C. M., Federice J. G., Bell C. N., Cox P. B., Njardarson J. T. (2024). An Update
on the Nitrogen Heterocycle Compositions and Properties of U.S. FDA-Approved
Pharmaceuticals (2013–2023). J. Med.
Chem..

[16] Hamill T. G., Krause S., Ryan C., Bonnefous C., Govek S., Seiders T. J., Cosford N. D. P., Roppe J., Kamenecka T., Patel S., Gibson R. E., Sanabria S., Riffel K., Eng W., King C., Yang X., Green M. D., O’Malley S. S., Hargreaves R., Burns H. D. (2005). Synthesis, characterization, and first successful monkey
imaging studies of metabotropic glutamate receptor subtype 5 (mGluR5)
PET radiotracers. Synapse.

[17] Vivash L., Gregoire M.-C., Lau E. W., Ware R. E., Binns D., Roselt P., Bouilleret V., Myers D. E., Cook M. J., Hicks R. J., O’Brien T. J. (2013). ^18^F-Flumazenil: A γ-Aminobutyric
Acid A–Specific PET Radiotracer for the Localization of Drug-Resistant
Temporal Lobe Epilepsy. J. Nucl. Med..

[18] Hayes H. L. D., Wei R., Assante M., Geogheghan K. J., Jin N., Tomasi S., Noonan G., Leach A. G., Lloyd-Jones G. C. (2021). Protodeboronation
of (Hetero)­Arylboronic Esters: Direct versus Prehydrolytic Pathways
and Self-/Auto-Catalysis. J. Am. Chem. Soc..

