# Supplementary material for: Ethyl Pinacol Boronates as Advantageous Precursors for Copper-Mediated Radiofluorination
Source: Org Lett. 2025 Jun 10;27(24):6545–50. doi: 10.1021/acs.orglett.5c02055 (PMC12186679; doi:10.1021/acs.orglett.5c02055)
Supplement: Supplementary file 1 [file ol5c02055_si_001.pdf]

Supporting Information for
Ethyl Pinacol Boronates as Advantageous Precursors for Copper-Mediated Radiofluorination

Nikolaos Hadjipaschalis [a], Sebastiano Ortalli [a], Zijun Chen [a], Robert S. Paton [b], Joseph Ford [a],*, Matthew Tredwell [c],[d],* and Véronique Gouverneur [a],*

[a] Department of Chemistry, Chemistry Research Laboratory, University of Oxford, Mansfield Road, Oxford, OX1 3TA, U.K.
[b] Department of Chemistry, Colorado State University, Fort Collins, Colorado 80528, United States.
[c] Wales Research and Diagnostic PET Imaging Centre, Cardiff University, University Hospital of Wales, Heath Park, Cardiff, CF14 4XN, U.K.
[d] School of Chemistry, Cardiff University, Main Building, Park Place, Cardiff, CF10 3AT, U.K.

*Correspondence should be addressed to veronique.gouverneur@chem.ox.ac.uk, tredwellm@cardiff.ac.uk, joseph.ford@chem.ox.ac.uk.

Table of Contents

General information S3

Preparation of starting materials, substrates and reference materials S4
  Preparation of aryl boronic ester substrates S4
  Preparation of aryl boronic 1,1,2,2-tetraethylethylene glycol (BEpin) esters S5
  Preparation of authentic reference compounds S18

Stability tests S20

Radiochemistry S26
  HPLC conditions S26
  Manual radiofluorination of aryl boronic esters S28
  Automated radiofluorination of FMZ-BEpin using a Trasis AllinOne radiosynthesizer . S31
  Quality control of [^{18}F]FMZ S33
  Radio and UV HPLC overlays and radiochemical yield calculations S44

Computations S64

References S70

NMR spectra for novel compounds S73

General information

Dry solvents were purchased from commercial suppliers or dried on a column of alumina. Reactions were monitored by thin-layer chromatography (TLC) on silica gel pre-coated aluminium sheets (Merck Kieselgel 60 F254 plates). Visualization was accomplished by irradiation with UV light at 254 nm, ceric ammonium molybdate and/or potassium permanganate and/or curcumin stain. Column chromatography was performed on Merck silica gel (60, particle size 0.040-0.063 mm). Preparative thin layer chromatography was performed on Uniplate silica gel GF 20x20 cm 500 µm plates. Deuterated solvents used for NMR experiments were purchased from commercial suppliers. All NMR spectra were recorded on Bruker AVIIIHD 400, AVIIIHD 500, AVII 500 or AV NEO 600. ^{1}H and ^{13}C NMR spectral data are reported as chemical shifts (δ) in parts per million (ppm) to 2 d.p. and 1 d.p., respectively, relative to the solvent peak using the Bruker internal referencing procedure (edlock). ^{19}F NMR spectra are reported as chemical shifts (δ) in parts per million (ppm) to 2 d.p. ^{19}F NMR spectra are externally referenced relative to $CFCl_3$. Coupling constants, *J*, are reported in Hz to the nearest 0.1 Hz. Unless otherwise stated, ^{13}C spectra are ^{1}H decoupled and reported coupling constants for ^{13}C spectra correspond to ^{19}F–^{13}C heteronuclear coupling, unless otherwise specified. Data are reported as follows: chemical shift, multiplicity (s = singlet, d = doublet, t = triplet, q = quartet, hept = heptet, br = broad, m = multiplet), coupling constants (Hz) and integration. NMR spectra were processed with MestReNova 15.0.1. IUPAC names were obtained using ChemDraw 19.1.1.32. High resolution mass spectra were determined on a Thermo Exactive mass spectrometer, for electrospray ionization (ESI-TOF), or an Agilent 7200 Accurate Mass Q-TOF GC-MS connected to a 7890 GC system, for electron ionization (GC-EI). Some compounds were found to be unstable under a variety of MS ionization methods (CI, EI, ESI, GC-MS) and therefore no HRMS could be obtained for them; this is stated for the relevant compounds. Infrared spectra were recorded as the neat compound (neat) or as an evaporated solution (thin layer film) using a Bruker Tensor 27 FT-IR spectrometer. Absorptions are reported in wavenumber (cm^{-1}). Melting points of solids were measured on a Griffin apparatus and are uncorrected.

For radiochemistry experiments: [^{18}F]Fluoride was produced in an IBA Cyclon 18/9 cyclotron using the $^{18}O(p,n)^{18}F$ reaction in PETIC (UK). Radiosynthesis was performed on an AllinOne radiosynthesizer (Trasis, Belgium).

All isolated activity yields are non-decay corrected (n.d.c.). All molar activities are decay corrected to the end of synthesis (EOS), unless stated otherwise.

Preparation of starting materials, substrates and reference materials

Preparation of aryl boronic ester substrates

4-(4,4,5,5-tetramethyl-1,3,2-dioxaborolan-2-yl)benzonitrile (**1b**), *N*,*N*-dimethyl-4-(4,4,5,5-tetramethyl-1,3,2-dioxaborolan-2-yl)aniline, 4-(4,4,5,5-tetramethyl-1,3,2-dioxaborolan-2-yl)quinoline, 1-methyl-5-(4,4,5,5-tetramethyl-1,3,2-dioxaborolan-2-yl)-1*H*-indole, 2-(benzo[*b*]thiophen-2-yl)-4,4,5,5-tetramethyl-1,3,2-dioxaborolane, 2-(4-(4,4,5,5-tetramethyl-1,3,2-dioxaborolan-2-yl)phenyl)pyridine, 6-(4,4,5,5-tetramethyl-1,3,2-dioxaborolan-2-yl)quinoline, 5-(4,4,5,5-tetramethyl-1,3,2-dioxaborolan-2-yl)benzo[*d*]oxazole, 5-(4,4,5,5-tetramethyl-1,3,2-dioxaborolan-2-yl)-1*H*-indazole, 4-((4-(4,4,5,5-tetramethyl-1,3,2-dioxaborolan-2-yl)phenyl)sulfonyl)morpholine, 4,4,5,5-tetramethyl-2-(perfluorophenyl)-1,3,2-dioxaborolane, 3-(4,4,5,5-tetramethyl-1,3,2-dioxaborolan-2-yl)pyridine, *N*-methyliminodiacetic acid, (4-cyanophenyl)boronic acid (**1d**), pinacol, 2,2'-(methylazanediyl)diacetic acid, naphthalene-1,8-diamine, and neopentyl glycol were purchased from commercial suppliers (Fluorochem, Sigma Aldrich, TCI, BLDpharm, Apollo Scientific, Ambeed, Manchester Organics, Biosynth) and used as received, without further purification.

Ethyl 5-methyl-6-oxo-8-(4,4,5,5-tetramethyl-1,3,2-dioxaborolan-2-yl)-5,6-dihydro-4*H*-benzo[*f*]imidazo[1,5-*a*][1,4]diazepine-3-carboxylate and 4-(1*H*-naphtho[1,8-*de*][1,3,2]diazaborinin-2(3*H*)-yl)benzonitrile (**1f**) were prepared exactly as described *via* literature procedures.[1,2]

4-(5,5-dimethyl-1,3,2-dioxaborinan-2-yl)benzonitrile (1c)

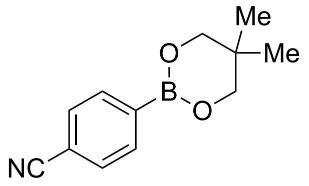

The title compound was prepared according to general procedure A (see below) using (4-cyanophenyl)boronic acid (147 mg, 1.0 mmol, 1.0 equiv) and neopentyl glycol (104 mg, 1.0 mmol, 1.0 equiv) in CH_2Cl_2 (0.1 M). The crude material was purified by flash column chromatography (pentane/EtOAc/AcOH = 79:20:1) to afford a white solid (197 mg, 92%).

^{1}H NMR (400 MHz, $CDCl_3$) δ 7.87 (d, J = 8.2 Hz, 2H), 7.62 (d, J = 8.3 Hz, 2H), 3.78 (s, 4H), 1.03 (s, 6H); **^{13}C NMR** (101 MHz, $CDCl_3$) δ 134.4, 131.2, 119.3, 114.1, 72.6, 32.1, 22.0; **HRMS** (ESI-TOF) m/z $[M+H]^+$ Calcd for $C_{12}H_{14}BNO_2$ 216.1190; Found 216.1184.

The recorded spectral data are in agreement with those reported in literature.[3]

4-(6-methyl-4,8-dioxo-1,3,6,2-dioxazaborocan-2-yl)benzonitrile (1e)

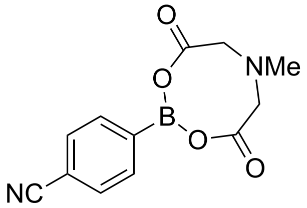

The title compound was synthesized *via* an adapted literature procedure.[4]

To a flame-dried round-bottom flask under N_2 were added (4-cyanophenyl)boronic acid (147 mg, 1.0 mmol, 1.0 equiv), *N*-methyliminodiacetic acid (MIDA) (442 mg, 3.0 mmol, 3.0 equiv) and 4 Å molecular sieves activated prior to use (360 mg) as solids. Anhydrous DMF (10 mL) was then added *via* syringe and the reaction mixture was stirred at 120 °C in an oil bath for 2 hours. The reaction mixture was then allowed to cool to room temperature and concentrated under reduced pressure. The residue was redissolved in acetone and filtered through celite to remove excess MIDA. The filtrate was concentrated under reduced pressure and purified by flash column chromatography (acetone). The product was further redissolved in a minimum volume of acetone and Et_2O was added to induce precipitation of a solid, which was triturated with additional Et_2O to afford the product as a white solid (138 mg, 53%).

^{1}H NMR (400 MHz, DMSO-*d*$_6$) δ 7.81 (d, J = 7.9 Hz, 2H), 7.63 (d, J = 7.8 Hz, 2H), 4.37 (d, J = 17.2 Hz, 2H), 4.15 (d, J = 17.2 Hz, 2H), 2.52 (s, 3H); **^{13}C NMR** (101 MHz, DMSO-*d*$_6$) δ 169.2, 133.4, 131.2, 119.0, 111.6, 62.1, 47.7; **HRMS** (ESI-TOF) m/z $[M+H]^+$ Calcd for $C_{12}H_{12}BN_2O_4$ 259.0885, found 259.0892; **M.P.** 251 °C; **IR** (neat) (cm^{-1}) 1760, 1456, 1395, 1337, 1299, 1257, 1238, 1222, 1179, 1113, 1088, 1068, 1043, 1011, 969, 876, 833, 765, 733, 705, 656, 640, 609.

Preparation of aryl boronic 1,1,2,2-tetraethylethylene glycol (BEpin) esters

[1,1′-bis(diphenylphosphino)ferrocene]dichloropalladium(II), 3,4-diethylhexane-3,4-diol (Epin) and 4,4,4',4',5,5,5',5'-octaethyl-2,2'-bi(1,3,2-dioxaborolane) (B_2Epin_2), (4-cyanophenyl)boronic acid (**1d**), (4-methoxyphenyl)boronic acid, [1,1'-biphenyl]-4-ylboronic acid, 3,4-dimethoxyphenyl)boronic acid, (4-(ethoxycarbonyl)phenyl)boronic acid, (4-(dimethylamino)phenyl)boronic acid, (4-(trifluoromethyl)phenyl)boronic acid, (3-nitrophenyl)boronic acid, quinolin-4-ylboronic acid, (1-methyl-1*H*-indol-5-yl)boronic acid, benzo[b]thiophen-2-ylboronic acid, 2-(4-bromophenyl)pyridine, 6-bromoquinoline, 5-bromoindolin-2-one, 5-bromobenzo[*d*]oxazole, mesitylboronic acid, (1*H*-indazol-5-yl)boronic acid, 1-(4-bromobenzyl)-1*H*-imidazole, (4-(methylsulfonyl)phenyl)boronic acid, 4-((4-bromophenyl)sulfonyl)morpholine, (perfluorophenyl)boronic acid, pyridin-3-ylboronic acid and ethyl 8-bromo-5-methyl-6-oxo-5,6-dihydro-4H-benzo[*f*]imidazo[1,5-*a*][1,4]diazepine-3-carboxylate were purchased from commercial suppliers (Fluorochem,

Sigma Aldrich, TCI, BLDpharm, Apollo Scientific, Ambeed, Manchester Organics, Biosynth) and used as received, without further purification.

4,4,5,5-tetraethyl-2-(naphthalen-1-yl)-1,3,2-dioxaborolane (**S4**) was prepared exactly as described *via* a literature procedure.[5]

General procedure A: Preparation of aryl-BEpin substrates *via* dehydrative esterification.
The following is adapted from a literature procedure.[5] To a flame-dried round-bottom flask under N_2 were added aryl boronic acid (1.0 equiv.) and 3,4-diethylhexane-3,4-diol (Epin) (1.0–1.1 equiv.) as solids. The flask was evacuated and backfilled with N_2 and anhydrous CH_2Cl_2 (0.1 M) was then added *via* syringe. The reaction mixture was stirred at room temperature for 16 hours. The reaction mixture was then diluted with water and extracted three times with CH_2Cl_2. The combined organics were dried over anhydrous Na_2SO_4 and concentrated under reduced pressure. The crude material was then purified by flash column chromatography.

General procedure B: Preparation of aryl-BEpin substrates *via* Pd-catalyzed Miyaura borylation.
The following is adapted from a literature procedure.[6] To a flame-dried Schlenk flask under N_2 were added aryl bromide (1.0 equiv.), B_2Epin_2 (1.1 equiv.), KOAc (3.0 equiv.) and Pd(dppf)Cl_2 (5 mol%) as solids. Anhydrous, degassed 1,4-dioxane (0.067 M) was then added *via* syringe. The reaction mixture was stirred at 80 °C for 16 hours in an oil bath. The reaction mixture was then allowed to cool to room temperature and filtered celite to remove any insoluble material, eluting with EtOAc. The filtrate was then concentrated under reduced pressure and purified by flash column chromatography.

4-(4,4,5,5-tetraethyl-1,3,2-dioxaborolan-2-yl)benzonitrile (1a)

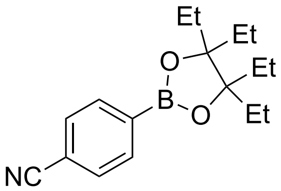

The title compound was prepared according to general procedure A using (4-cyanophenyl)boronic acid (147 mg, 1.0 mmol, 1.0 equiv) and Epin (174 mg, 1.0 mmol, 1.0 equiv) in CH_2Cl_2 (0.1 M). The crude material was purified by flash column chromatography (pentane/EtOAc = 95:5) to afford a colorless oil (194 mg, 68%).

^{1}H NMR (400 MHz, $CDCl_3$) δ 7.89 (d, J = 8.3 Hz, 2H), 7.63 (d, J = 8.3 Hz, 2H), 1.85 – 1.66 (m, 8H), 0.96 (t, J = 7.5 Hz, 12H); **^{13}C NMR** (101 MHz, $CDCl_3$) δ 135.3, 131.3, 119.1, 114.5, 89.7, 26.6, 8.9; **HRMS** (ESI-TOF) m/z $[M+H]^+$ Calcd for $C_{17}H_{25}BNO_2$ 286.1973; Found 286.1969; **IR** (neat) (cm^{-1}) 2978, 1460, 1400, 1366, 1269, 1090, 1022, 912, 838, 733, 649.

2-([1,1'-biphenyl]-4-yl)-4,4,5,5-tetraethyl-1,3,2-dioxaborolane (S3)

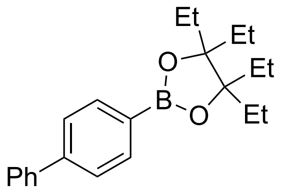

The title compound was prepared according to general procedure A using [1,1'-biphenyl]-4-ylboronic acid (100 mg, 0.5 mmol, 1.0 equiv) and Epin (88 mg, 0.5 mmol, 1.0 equiv) in CH_2Cl_2 (0.1 M). The crude material was purified by flash column chromatography (hexane/EtOAc = 75:25) to afford a colorless solid (126 mg, 74%).

^{1}H NMR (400 MHz, $CDCl_3$) δ 7.95 (d, J = 8.3 Hz, 2H), 7.71 – 7.61 (m, 4H), 7.53 – 7.44 (m, 2H), 7.42 – 7.36 (m, 1H), 1.94 – 1.71 (m, 8H), 1.03 (t, J = 7.5 Hz, 12H); **^{13}C NMR** (101 MHz, $CDCl_3$) δ 143.9, 141.3, 135.4, 128.9, 127.6, 127.4, 126.6, 88.9, 26.6, 9.0; **HRMS** (ESI-TOF) m/z: $[M+H]^+$ Calcd for $C_{22}H_{30}BO_2$ 337.2333; Found 337.2344; **MP** 50 °C; **IR** (neat) (cm^{-1}) 2968, 1611, 1600, 1524, 1456, 1402, 1385, 1369, 1349, 1314, 1293, 1261, 1184, 1097, 1025, 1010, 919, 844, 821, 766, 741, 699, 674, 658.

4,4,5,5-tetraethyl-2-mesityl-1,3,2-dioxaborolane (S5)

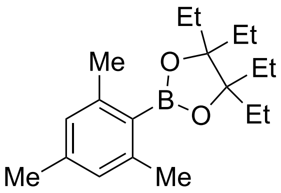

The title compound was prepared according to general procedure A using toluene as the solvent and heating the reaction mixture at 80 °C with mesitylboronic acid (164 mg, 1.0 mmol, 1.0 equiv) and Epin (192 mg, 1.1 mmol, 1.1 equiv) in CH_2Cl_2 (0.1 M). The crude material was purified by flash column chromatography (pentane/EtOAc = 95:5) to afford a yellow oil (60 mg, 20%).

^{1}H NMR (400 MHz, $CDCl_3$) δ 6.78 (s, 2H), 2.39 (s, 6H), 2.24 (s, 3H), 1.90 – 1.69 (m, 8H), 0.97 (t, J = 7.5 Hz, 12H); **^{13}C NMR** (101 MHz, $CDCl_3$) δ 142.9, 139.2, 127.8, 88.8, 26.3, 22.6, 21.3, 9.0; **HRMS** (ESI-TOF) m/z: $[M+H]^+$ Calcd for $C_{19}H_{32}BO_2$ 303.2490; Found 303.2488; **IR** (neat) (cm^{-1}) 2973, 1730, 1611, 1586, 1457, 1435, 1399, 1328, 1289, 1173, 1115, 1071, 958, 921, 848, 803, 782, 736, 657.

4,4,5,5-tetraethyl-2-(4-methoxyphenyl)-1,3,2-dioxaborolane (S6)

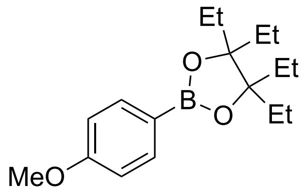

The title compound was prepared according to general procedure A using (4-methoxyphenyl)boronic acid (152 mg, 1.0 mmol, 1.0 equiv) and Epin (174 mg, 1.0 mmol, 1.0 equiv) in CH_2Cl_2 (0.1 M). The crude material was purified by flash column chromatography (pentane/EtOAc = 90:10) to afford a colorless oil (95 mg, 33%).

^{1}H NMR (400 MHz, $CDCl_3$) δ 7.78 (d, J = 8.7 Hz, 2H), 6.90 (d, J = 8.7 Hz, 2H), 3.83 (s, 3H), 1.84 – 1.67 (m, 8H), 0.97 (t, J = 7.5 Hz, 12H); **^{13}C NMR** (101 MHz, $CDCl_3$) δ 162.2, 136.6, 113.4, 88.6, 55.2, 26.6, 9.0; **HRMS** (ESI-TOF) m/z: $[M+H]^+$ Calcd for $C_{17}H_{28}BO_3$ 291.2126; Found 291.2120; **IR** (neat) (cm^{-1}) 2976, 1606, 1571, 1459, 1398, 1366, 1352, 1307, 1247, 1175, 1093, 1033, 923, 833, 735, 652.

2-(3,4-dimethoxyphenyl)-4,4,5,5-tetraethyl-1,3,2-dioxaborolane (S7)

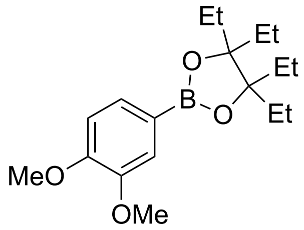

The title compound was prepared according to general procedure A using (3,4-dimethoxyphenyl)boronic acid (182 mg, 1.0 mmol, 1.0 equiv) and Epin (192 mg, 1.1 mmol, 1.1 equiv) in CH_2Cl_2 (0.1 M). The crude material was purified by flash column chromatography (pentane/EtOAc = 90:10) to afford a colorless oil (190 mg, 59%).

^{1}H NMR (400 MHz, $CDCl_3$) δ 7.44 (d, J = 8.1 Hz, 1H), 7.30 (s, 1H), 6.88 (d, J = 8.0 Hz, 1H), 3.92 (s, 3H), 3.90 (s, 3H), 1.86 – 1.65 (m, 8H), 0.97 (t, J = 7.5 Hz, 12H); **^{13}C NMR** (101 MHz, $CDCl_3$) δ 151.7, 148.5, 128.7, 116.8, 110.7, 88.7, 56.0, 55.9, 26.7, 8.9; **HRMS** (ESI-TOF) m/z: $[M+H]^+$ Calcd for $C_{18}H_{30}BO_4$ 321.2232; Found 321.2228; **IR** (neat) (cm^{-1}) 2976, 1600, 1519, 1452, 1411, 1352, 1256, 1227, 1176, 1140, 1115, 1098, 1029, 969, 929, 873, 814, 758, 738, 699, 683, 616.

4,4,5,5-tetraethyl-2-(4-(trifluoromethyl)phenyl)-1,3,2-dioxaborolane (S8)

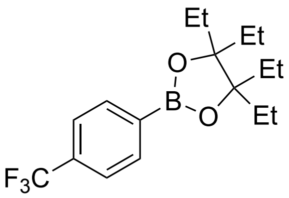

The title compound was prepared according to general procedure A using (4-(trifluoromethyl)phenyl)boronic acid (189 mg, 1.0 mmol, 1.0 equiv) and Epin (192 mg, 1.1 mmol, 1.1 equiv) in CH_2Cl_2 (0.1 M). The crude material was purified by flash column chromatography (pentane/EtOAc = 85:15) to afford a colorless oil (284 mg, 87%).

^{1}H NMR (400 MHz, $CDCl_3$) δ 7.96 – 7.89 (m, 2H), 7.65 – 7.58 (m, 2H), 1.87 – 1.67 (m, 8H), 0.97 (t, *J* = 7.5 Hz, 12H); **^{13}C NMR** (101 MHz, $CDCl_3$) δ 135.2, 132.8 (q, *J* = 32.1 Hz), 124.4 (q, *J* = 3.8 Hz), 124.3 (q, *J* = 272.4 Hz) 89.4, 26.6, 9.0; **^{19}F NMR** (377 MHz, $CDCl_3$): -63.00 (s); **HRMS** (EI) m/z: $[M]^{\cdot+}$ Calcd for $C_{17}H_{24}BF_3O_2$ 327.18523; Found 327.18454; **IR** (neat) (cm^{-1}) 2980, 1521, 1460, 1404, 1369, 1321, 1165, 1129, 1096, 1064, 1020, 920, 841, 767, 656.

ethyl 4-(4,4,5,5-tetraethyl-1,3,2-dioxaborolan-2-yl)benzoate (S9)

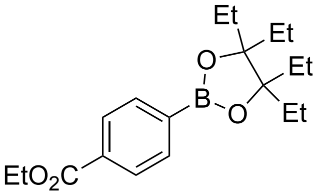

The title compound was prepared according to general procedure A using (4-(ethoxycarbonyl)phenyl)boronic acid (194 mg, 1.0 mmol, 1.0 equiv) and Epin (174 mg, 1.0 mmol, 1.0 equiv) in CH_2Cl_2 (0.1 M). The crude material was purified by flash column chromatography (pentane/EtOAc = 90:10) to afford a colorless oil (292 mg, 88%).

^{1}H NMR (400 MHz, $CDCl_3$) δ 8.02 (d, *J* = 7.7 Hz, 2H), 7.88 (d, *J* = 7.8 Hz, 2H), 4.38 (q, *J* = 7.1 Hz, 2H), 1.87 – 1.66 (m, 8H), 1.39 (t, *J* = 7.1 Hz, 3H), 0.97 (t, *J* = 7.4 Hz, 12H); **^{13}C NMR** (101 MHz, $CDCl_3$) δ 166.8, 134.8, 132.7, 128.6, 89.3, 61.1, 26.6, 14.4, 8.9; **HRMS** (ESI-TOF) m/z: $[M+H]^+$ Calcd for $C_{19}H_{30}BO_4$ 333.2232; Found 333.2236; **IR** (neat) (cm^{-1}) 2979, 1721, 1510, 1460, 1401, 1365, 1353, 1309, 1269, 1177, 1108, 1097, 1022, 920, 860, 815, 771, 734, 710, 649.

4,4,5,5-tetraethyl-2-(3-nitrophenyl)-1,3,2-dioxaborolane (S10)

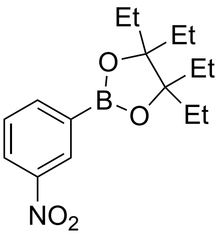

The title compound was prepared according to general procedure A using (3-nitrophenyl)boronic acid (167 mg, 1.0 mmol, 1.0 equiv) and Epin (192 mg, 1.1 mmol, 1.1 equiv) in CH_2Cl_2 (0.1 M). The crude material was purified by flash column chromatography (pentane/EtOAc = 90:10) to afford a white solid (280 mg, 92%).

^{1}H NMR (400 MHz, $CDCl_3$) δ 8.63 (d, J = 1.9 Hz, 1H), 8.32 – 8.25 (m, 1H), 8.11 (d, J = 7.3 Hz, 1H), 7.54 (t, J = 7.8 Hz, 1H), 1.87 – 1.67 (m, 8H), 0.97 (t, J = 7.5 Hz, 12H); **^{13}C NMR** (101 MHz, $CDCl_3$) δ 148.0, 140.9, 129.5, 128.9, 125.9, 89.8, 26.6, 8.9; **HRMS** (ESI-TOF) m/z: $[M+Na]^+$ Calcd for $C_{16}H_{24}BNO_4Na$ 328.1691; Found 328.1701; **MP** 35–36 °C; **IR** (neat) (cm^{-1}) 2978, 1616, 1529, 1486, 1458, 1429, 1387, 1368, 1358, 1343, 1317, 1271, 1183, 1111, 956, 921, 881, 813, 772, 740, 695, 650.

4,4,5,5-tetraethyl-2-(4-(methylsulfonyl)phenyl)-1,3,2-dioxaborolane (S11)

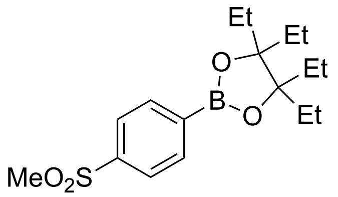

The title compound was prepared according to general procedure A using (4-(methylsulfonyl)phenyl)boronic acid (200 mg, 1.0 mmol, 1.0 equiv) and Epin (192 mg, 1.1 mmol, 1.1 equiv) in CH_2Cl_2 (0.1 M). The crude material was purified by flash column chromatography (pentane/EtOAc = 100:0 to 90:10) to afford a colorless oil (72 mg, 21%).

^{1}H NMR (500 MHz, $CDCl_3$) δ 8.00 (d, J = 8.4 Hz, 2H), 7.92 (d, J = 8.2 Hz, 2H), 3.04 (s, 3H), 1.85–1.68 (m, 8H), 0.97 (t, J = 7.5 Hz, 12H); **^{13}C NMR** (126 MHz, $CDCl_3$) δ 142.6, 135.7, 126.4, 89.7, 44.6, 26.6, 9.0; **HRMS** (ESI-TOF) m/z: $[M+H]^+$ Calcd for $C_{17}H_{28}BO_4S$ 339.1796; Found 339.1790; **IR** (neat) (cm^{-1}) 2978, 1602, 1459, 1396, 1365, 1353, 1316, 1269, 1154, 1096, 1080, 1020, 957, 917, 838, 767, 730, 706, 649.

4-((4-(4,4,5,5-tetraethyl-1,3,2-dioxaborolan-2-yl)phenyl)sulfonyl)morpholine (S12)

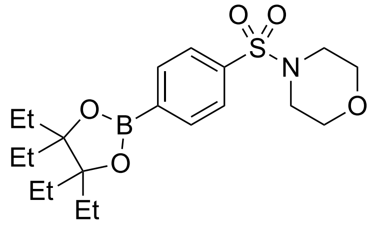

The title compound was prepared according to general procedure B using 4-((4-bromophenyl)sulfonyl)morpholine (306 mg, 1 mmol, 1.0 equiv) in 1,4-dioxane (0.067 M). The

crude material was purified by flash column chromatography (hexane/EtOAc = 100:0 to 80:20, then CH_2Cl_2/hexane = 50:50 to 100:0) to afford a colorless viscous oil (368 mg, 90%).

^{1}H NMR (400 MHz, $CDCl_3$) *δ* 8.07 – 7.91 (m, 2H), 7.79 – 7.67 (m, 2H), 3.81 – 3.66 (m, 4H), 3.07 – 2.90 (m, 4H), 1.87 – 1.67 (m, 8H), 0.97 (t, *J* = 7.5 Hz, 12H); **^{13}C NMR** (101 MHz, $CDCl_3$) *δ* 137.2, 135.5, 126.9, 89.7, 66.3, 46.1, 26.6, 9.0; **HRMS** (ESI-TOF) m/z: $[M+H]^+$ Calcd for $C_{20}H_{33}BNO_5S$ 410.2167; Found 410.2164; **IR** (neat) (cm^{-1}) 2976, 1600, 1454, 1396, 1353, 1297, 1262, 1216, 1170, 1115, 1101, 1081, 1019, 945, 919, 838, 745, 733, 705, 652, 613.

The recorded spectral data are in agreement with those reported in literature.[7]

2-(4-(4,4,5,5-tetraethyl-1,3,2-dioxaborolan-2-yl)phenyl)pyridine (S13)

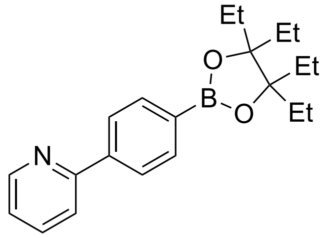

The title compound was prepared according to general procedure B using 2-(4-bromophenyl)pyridine (234 mg, 1.0 mmol, 1.0 equiv) in 1,4-dioxane (0.067 M). The crude material was purified by flash column chromatography (CH_2Cl_2/pentane = 5:1) to afford a colorless oil (265 mg, 79 %).

^{1}H NMR (400 MHz, $CDCl_3$) *δ* 8.73 – 8.69 (m, 1H), 8.01 (d, *J* = 8.3 Hz, 2H), 7.94 (d, *J* = 8.3 Hz, 2H), 7.79 – 7.69 (m, 2H), 7.25 – 7.20 (m, 1H), 1.89 – 1.68 (m, 8H), 0.99 (t, *J* = 7.5 Hz, 12H); **^{13}C NMR** (101 MHz, $CDCl_3$) *δ* 157.5, 149.8, 141.8, 136.8, 135.3, 126.2, 122.4, 120.9, 89.0, 26.6, 9.0; **HRMS** (ESI-TOF) m/z: $[M+H]^+$ Calcd for $C_{21}H_{29}BNO_2$ 338.2286; Found 338.2277; IR (neat) **IR** (thin layer film) (cm^{-1}) 2977, 1610, 1586, 1553, 1517, 1465, 1398, 1366, 1352, 1312, 1291, 1257, 1098, 1018, 920, 853, 821, 783, 735, 654, 617.

4-(4,4,5,5-tetraethyl-1,3,2-dioxaborolan-2-yl)quinoline (S14)

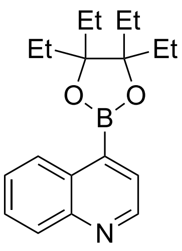

The title compound was prepared according to general procedure A using quinolin-4-ylboronic acid (173 mg, 1.0 mmol, 1.0 equiv) and Epin (192 mg, 1.1 mmol, 1.1 equiv) in CH_2Cl_2 (0.1 M). The crude material was purified by flash column chromatography (pentane/EtOAc = 95:5) to afford a white solid (91 mg, 29%).

^{1}H NMR (400 MHz, $CDCl_3$) *δ* 8.93 (d, *J* = 4.2 Hz, 1H), 8.68 (d, *J* = 10.0 Hz, 1H), 8.12 (d, *J* = 9.8 Hz, 1H), 7.89 (d, *J* = 4.2 Hz, 1H), 7.75 – 7.66 (m, 1H), 7.64 – 7.54 (m, 1H), 1.95 – 1.74 (m, 8H), 1.03 (t, *J* = 7.5 Hz, 12H); **^{13}C NMR** (101 MHz, $CDCl_3$) *δ* 149.6, 147.9, 131.4, 129.7, 129.2, 129.1, 128.5, 127.0, 89.8, 26.7, 9.1; **HRMS** (ESI-TOF) m/z: $[M+H]^+$ Calcd for $C_{19}H_{27}BNO_2$ 312.2129; Found 312.2130; **MP** 59 – 61 °C; **IR** (neat) (cm^{-1}) 2980, 1561, 1510, 1454, 1411, 1379, 1360, 1330, 1302, 1287, 1259, 1194, 1152, 1118, 1093, 1025, 994, 960, 944, 922, 891, 873, 859, 765, 684, 662, 636, 624.

6-(4,4,5,5-tetraethyl-1,3,2-dioxaborolan-2-yl)quinoline (S15)

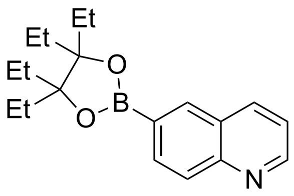

The title compound was prepared according to general procedure B using 6-bromoquinoline (208 mg, 1.0 mmol, 1.0 equiv) in 1,4-dioxane (0.067 M). The crude material was purified by flash column chromatography (pentane/EtOAc = 80:20 to 30:70) to afford a colorless oil (249 mg, 80%).

^{1}H NMR (400 MHz, $CDCl_3$) *δ* 8.93 (dd, *J* = 4.3, 1.8 Hz, 1H), 8.34 (s, 1H), 8.20 (dd, *J* = 8.3, 2.0 Hz, 1H), 8.14 – 8.04 (m, 2H), 7.39 (dd, *J* = 8.2, 4.3 Hz, 1H), 1.90 – 1.70 (m, 8H), 1.00 (t, *J* = 7.5 Hz, 12H); **^{13}C NMR** (101 MHz, $CDCl_3$) *δ* 151.4, 149.9, 136.8, 136.1, 134.5, 128.6, 127.8, 121.2, 89.3, 26.7, 9.0; **HRMS** (ESI-TOF) m/z: $[M+H]^+$ Calcd for $C_{19}H_{27}BNO_2$ 312.2129; Found 312.2126; **IR** (neat) (cm^{-1}) 2977, 2361, 1622, 1568, 1460, 1424, 1363, 1290, 1229, 1186, 1116, 1081,1031, 951, 922, 843, 801, 774, 734, 700, 685, 621.

1-methyl-5-(4,4,5,5-tetraethyl-1,3,2-dioxaborolan-2-yl)-1*H*-indole (S16)

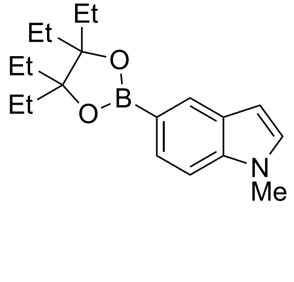

The title compound was prepared according to general procedure A using (1-methyl-1*H*-indol-5-yl)boronic acid (175 mg, 1.0 mmol, 1.0 equiv) and Epin (192 mg, 1.1 mmol, 1.1 equiv) in CH_2Cl_2 (0.1 M). The crude material was purified by flash column chromatography (pentane/EtOAc = 95:5) to afford a beige solid (88 mg, 28%).

^{1}H NMR (400 MHz, $CDCl_3$) δ 8.18 (s, 1H), 7.69 (d, J = 8.0 Hz, 1H), 7.32 (d, J = 8.3 Hz, 1H), 7.04 (d, J = 3.1 Hz, 1H), 6.51 (d, J = 3.1 Hz, 1H), 3.79 (s, 3H), 1.90 – 1.68 (m, 8H), 1.00 (t, J = 7.4 Hz, 12H); **^{13}C NMR** (101 MHz, $CDCl_3$) δ 138.7, 129.0, 128.9, 128.4, 127.9, 108.7, 101.8, 88.5, 32.9, 26.7, 9.1; **HRMS** (ESI-TOF) m/z: $[M+H]^+$ Calcd for $C_{19}H_{29}BNO_2$ 314.2286; Found 314.2290.

The recorded spectral data are in agreement with those reported in literature.[7]

5-(4,4,5,5-tetraethyl-1,3,2-dioxaborolan-2-yl)benzo[*d*]oxazole (S17)

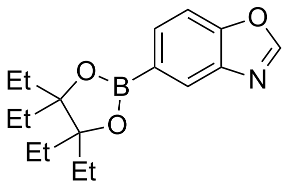

The title compound was prepared according to general procedure B using 5-bromobenzo[*d*]oxazole (198 mg, 1.0 mmol, 1.0 equiv) in 1,4-dioxane (0.067 M). The crude material was purified by flash column chromatography (pentane/EtOAc = 90:10) to afford a white solid (288 mg, 96%).

^{1}H NMR (400 MHz, $CDCl_3$) δ 8.27 (s, 1H), 8.09 (s, 1H), 7.87 (d, J = 8.2 Hz, 1H), 7.57 (d, J = 8.9 Hz, 1H), 1.89 – 1.68 (m, 8H), 0.98 (t, J = 7.5 Hz, 12H); **^{13}C NMR** (101 MHz, $CDCl_3$) δ 152.6, 152.2, 139.9, 132.3, 127.6, 110.6, 89.2, 26.6, 9.0; **HRMS** (ESI-TOF) m/z: $[M+H]^+$ Calcd for $C_{17}H_{25}BNO_3$ 302.1922; Found 302.1929; **MP** 49–50 °C; **IR** (neat) (cm^{-1}) 3091, 2979, 1613, 1520, 1483, 1455, 1421, 1396, 1385, 1363, 1346, 1294, 1261,1222, 1184, 1162, 1141, 1112, 1077, 1050, 970, 930, 902, 855, 820, 781, 707, 691, 675, 626.

***N*,*N*-dimethyl-4-(4,4,5,5-tetraethyl-1,3,2-dioxaborolan-2-yl)aniline (S18)**

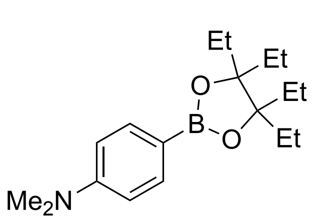

The title compound was prepared according to general procedure A using (4-(dimethylamino)phenyl)boronic acid (165 mg, 1.0 mmol, 1.0 equiv) and Epin (192 mg, 1.1

mmol, 1.1 equiv) in CH_2Cl_2 (0.1 M). The crude material was purified by flash column chromatography (pentane/EtOAc = 100:0 to 98:2) to afford an off-white solid (29 mg, 10%).

^{1}H NMR (400 MHz, $CDCl_3$) δ 7.71 (d, J = 8.8 Hz, 2H), 6.70 (d, J = 8.8 Hz, 2H), 2.98 (s, 6H), 1.92 – 1.60 (m, 8H), 0.96 (t, J = 7.5 Hz, 12H); **^{13}C NMR** (101 MHz, $CDCl_3$) δ 152.6, 136.3, 111.4, 88.2, 40.3, 26.6, 9.0; **HRMS** (ESI-TOF) m/z: $[M+H]^+$ $C_{18}H_{31}BNO_2$ Calcd for 304.2442; Found 304.2438; **MP** 29–30 °C; **IR** (neat) (cm^{-1}) 2923, 2855, 1731, 1605, 1530, 1459, 1401, 1361, 1309, 1292, 1264, 1230, 1195, 1170, 1120, 1094, 1064, 1022, 947, 926, 816, 739, 705, 656, 621.

4,4,5,5-tetraethyl-2-(perfluorophenyl)-1,3,2-dioxaborolane (S19)

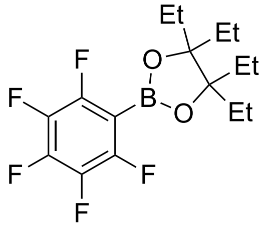

The title compound was prepared according to general procedure A using (perfluorophenyl)boronic acid (212 mg, 1.0 mmol, 1.0 equiv) and Epin (192 mg, 1.1 mmol, 1.1 equiv) in CH_2Cl_2 (0.1 M). The crude material was purified by flash column chromatography (pentane/EtOAc = 95:5) to afford a white solid (235 mg, 67%).

^{1}H NMR (600 MHz, $CDCl_3$) δ 1.86 – 1.70 (m, 8H), 0.98 (t, J = 7.4 Hz, 12H); **^{13}C NMR** (151 MHz, $CDCl_3$) δ 150.6 – 148.1 (m), 144.3 – 141.8 (m), 138.6 – 135.9 (m), 90.3, 26.4, 8.8; **^{19}F NMR** (565 MHz, $CDCl_3$) δ -129.28 – -129.45 (m), -150.05 – -150.21 (m), -161.95 – -162.17 (m); **HRMS** the title compound did not ionize; **MP** 42 – 43 °C; **IR** (neat) (cm^{-1}) 2987, 2945, 2889, 1651, 1524, 1486, 1462, 1410, 1387, 1362, 1344, 1291, 1258, 1180, 1114, 1099, 1025, 977, 920, 852, 763, 732, 673.

3-(4,4,5,5-tetraethyl-1,3,2-dioxaborolan-2-yl)pyridine (S20)

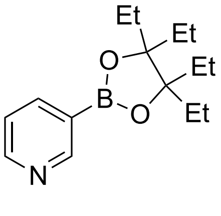

The title compound was prepared following a procedure adapted from the literature.[5]

To a flame-dried round-bottom flask under N_2 were added pyridin-3-ylboronic acid (123 mg, 1.0 mmol, 1.0 equiv) and 3,4-diethylhexane-3,4-diol (192 mg, 1.1 mmol, 1.1 equiv) as solids. The flask was evacuated and backfilled with N_2 and anhydrous CH_2Cl_2 (0.1 M) was then added

via syringe, followed by AcOH (63 µL, 1.1 mmol, 1.1 equiv). The reaction mixture was stirred at room temperature for 16 hours. The reaction mixture was then diluted with water and extracted three times with CH_2Cl_2. The combined organics were dried over anhydrous $MgSO_4$ and concentrated under reduced pressure. The crude material was then purified by flash column chromatography (hexane/EtOAc = 50:50) to afford a white solid (82 mg, 31%).

^{1}H NMR (500 MHz, $CDCl_3$) δ 8.95 (s, 1H), 8.68 – 8.63 (m, 1H), 8.08 – 8.02 (m, 1H), 7.29 – 7.23 (m, 1H), 1.85 – 1.66 (m, 8H), 0.96 (t, *J* = 7.5 Hz, 12H); **^{13}C NMR** (126 MHz, $CDCl_3$) δ 155.7, 152.0, 142.4, 123.2, 89.4, 26.6, 8.9; **HRMS** (ESI-TOF) m/z: $[M+H]^+$ Calcd for $C_{15}H_{25}BNO_2$ 262.1973; Found 262.1964.

The recorded spectral data are in agreement with those reported in literature.[5]

5-(4,4,5,5-tetraethyl-1,3,2-dioxaborolan-2-yl)-1*H*-indazole (S21)

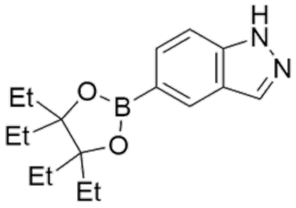

The title compound was prepared according to general procedure A using (1*H*-indazol-5-yl)boronic acid (162 mg, 1.0 mmol, 1.0 equiv) and Epin (192 mg, 1.1 mmol, 1.1 equiv) in CH_2Cl_2 (0.1 M). The crude material was purified by flash column chromatography (pentane/EtOAC = 80:20) to afford a white solid (100 mg, 33%).

^{1}H NMR (400 MHz, $CDCl_3$) δ 10.09 (s, 1H), 8.32 (t, *J* = 1.0 Hz, 1H), 8.10 (d, *J* = 1.1 Hz, 1H), 7.83 (dd, *J* = 8.4, 1.0 Hz, 1H), 7.48 (dt, *J* = 8.4, 1.0 Hz, 1H), 1.89 – 1.68 (m, 8H), 0.99 (t, *J* = 7.5 Hz, 12H); **^{13}C NMR** (101 MHz, $CDCl_3$) δ 141.6, 135.7, 132.6, 129.1, 123.2, 108.8, 88.8, 26.5, 8.9; **HRMS** (ESI-TOF) m/z: $[M+H]^+$ Calcd for $C_{17}H_{26}BN_2O_2$ 301.2082; Found 301.2078; **MP** 116– 117 °C; **IR** (neat) (cm^{-1}) 3189, 3145, 3044, 2945, 2880, 1625, 1517, 1458, 1410, 1388, 1367, 1354, 1329, 1307, 1289, 1184, 1129, 1112, 1084, 953, 935, 915, 841, 808, 774, 757, 696, 679, 620.

1-(4-(4,4,5,5-tetraethyl-1,3,2-dioxaborolan-2-yl)benzyl)-1*H*-imidazole (S22)

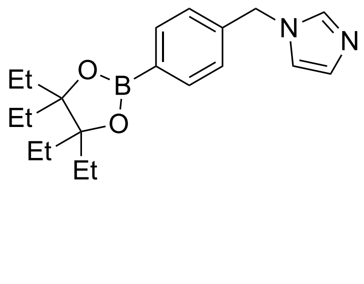

The title compound was prepared according to general procedure B from 1-(4-bromobenzyl)-1*H*-imidazole (237, 1.0 mmol, 1.0 equiv) in 1,4-dioxane (0.067 M). The crude material was purified by flash column chromatography (EtOAc) to afford a brown oil (60 mg, 18%).

^{1}H NMR (400 MHz, $CDCl_3$) δ 7.80 (d, J = 8.2 Hz, 2H), 7.54 (s, 1H), 7.14 (d, J = 8.2 Hz, 2H), 7.08 (s, 1H), 6.88 (s, 1H), 5.12 (s, 2H), 1.86 – 1.67 (m, 8H), 0.96 (t, J = 7.5 Hz, 12H); **^{13}C NMR** (101 MHz, $CDCl_3$) δ 139.0, 137.6, 135.6, 130.0, 126.7, 119.4, 89.1, 51.0, 26.6, 9.0; **HRMS** (ESI-TOF) m/z: $[M+H]^+$ Calcd for $C_{20}H_{30}BN_2O_2$ 341.2395; Found 341.2388; **IR** (neat) (cm^{-1}) 2976, 2884, 2361, 1614, 1505, 1459, 1401, 1366, 1352, 1313, 1289, 1232, 1183, 1108, 1091, 1023, 989, 922, 818, 792, 735, 702, 661.

2-(benzo[*b*]thiophen-2-yl)-4,4,5,5-tetraethyl-1,3,2-dioxaborolane (S23)

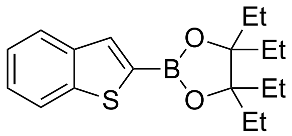

The title compound was prepared according to general procedure A using benzo[*b*]thiophen-2-ylboronic acid (178 mg, 1.0 mmol, 1.0 equiv) and Epin (192 mg, 1.1 mmol, 1.1 equiv) in CH_2Cl_2 (0.1 M). The crude material was purified by flash column chromatography (pentane/EtOAc = 95:5) to afford a white solid (291 mg, 92%).

^{1}H NMR (400 MHz, $CDCl_3$) δ 7.93 – 7.81 (m, 3H), 7.41 – 7.30 (m, 2H), 1.90 – 1.67 (m, 8H), 0.99 (t, J = 7.5 Hz, 12H); **^{13}C NMR** (101 MHz, $CDCl_3$) δ 143.9, 140.7, 134.4, 125.3, 124.4, 124.2, 122.7, 89.6, 26.6, 9.0; **HRMS** (ESI-TOF) m/z: $[M]^+$ Calcd for $C_{18}H_{25}BO_2S$ 316.1663; Found 316.1661; **M.P.** 75–77 °C; **IR** (neat) (cm^{-1}) 2978, 2962, 2932, 2880, 1557, 1522, 1456, 1438, 1357, 1347, 1312, 1285, 1179, 1159, 1141, 1110, 1028, 1004, 909, 865, 842, 776, 749, 729, 685, 665.

3-(pyridin-2-ylethynyl)-5-(4,4,5,5-tetraethyl-1,3,2-dioxaborolan-2-yl)benzonitrile (S24)

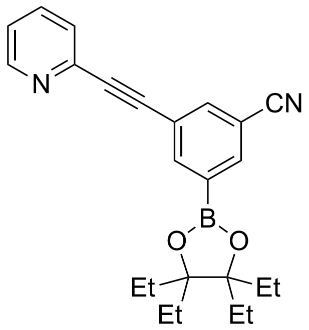

The title compound was prepared according to general procedure B using 3-bromo-5-(pyridin-2-ylethynyl)benzonitrile (89 mg, 0.31 mmol, 1.0 equiv) in 1,4-dioxane (0.067 M). The crude material was purified by flash column chromatography (pentane/EtOAc = 100:0 to 80:20)

followed by preparative thin layer chromatography (pentane/EtOAc = 80:20) to afford an off-white solid (21 mg, 17%).

^{1}H NMR (500 MHz, $CDCl_3$) δ 8.64 (d, J = 5.2 Hz, 1H), 8.24 (s, 1H), 8.05 (s, 1H), 7.90 (s, 1H), 7.71 (t, J = 7.6 Hz, 1H), 7.54 (d, J = 7.8 Hz, 1H), 7.28 (dd, J = 7.8, 4.7 Hz, 1H), 1.83–1.69 (m, 8H), 0.97 (t, J = 7.5 Hz, 12H); **^{13}C NMR** (126 MHz, $CDCl_3$) δ 150.4, 142.9, 142.4, 138.3, 137.1, 136.4, 127.5, 123.4, 123.4, 118.2, 112.7, 90.7, 90.0, 86.8, 26.6, 8.9; **HRMS** (ESI-TOF) m/z: $[M+H]^+$ Calcd for $C_{24}H_{27}BN_2O_2$ 387.2238; Found 387.2232; **MP** 124 – 126 °C.

Ethyl 5-methyl-6-oxo-8-(4,4,5,5-tetraethyl-1,3,2-dioxaborolan-2-yl)-5,6-dihydro-4*H*-benzo[*f*]imidazo[1,5-*a*][1,4]diazepine-3-carboxylate (25)

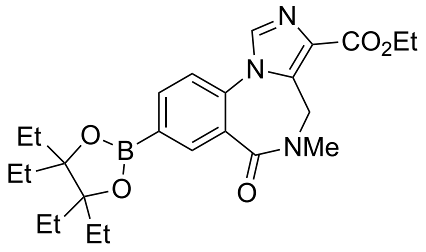

To a dry Schlenk flask under N_2 atmosphere and equipped with a stir bar was added ethyl 8-bromo-5-methyl-6-oxo-5,6-dihydro-4*H*-benzo[*f*]imidazo[1,5-*a*][1,4]diazepine-3-carboxylate (146 mg, 0.4 mmol, 1.0 equiv.), B_2Epin_2 (220 mg, 0.6 mmol, 1.5 equiv.), Pd(dppf)Cl_2 (15 mg, 0.02 mmol, 5 mol%), KOAc (118 mg, 1.2 mmol, 3 equiv.) and DMSO (4 mL, 0.1 M) as the solvent. The reaction mixture was heated at 80 °C for 16 h. The reaction was then cooled to rt and then filtered through a celite plug, eluting EtOAc. The filtrate was transferred to a separatory funnel containing water (40 mL) and brine (10 mL) and extracted with EtOAc. The organic layers were combined and dried over Na_2SO_4. The volatiles were evaporated under reduced pressure. The crude material was purified by flash column chromatography (CH_2Cl_2/acetone = 75:25) to afford a white solid (153 mg, 82%).

^{1}H NMR (400 MHz, $CDCl_3$) δ 8.50 (d, J = 1.4 Hz, 1H), 8.02 (dd, J = 7.9, 1.5 Hz, 1H), 7.89 (s, 1H), 7.39 (d, J = 8.0 Hz, 1H), 5.29 – 5.10 (m, 1H), 4.54 – 4.19 (m, 3H), 3.24 (s, 3H), 1.86 – 1.66 (m, 8H), 1.44 (t, J = 7.2 Hz, 3H), 0.96 (t, J = 7.5 Hz, 12H); **^{13}C NMR** (101 MHz, $CDCl_3$) δ 166.8, 163.2, 139.3, 138.8, 135.8, 135.0, 133.9, 128.9, 128.5, 121.1, 89.6, 61.1, 42.4, 35.9, 26.6, 14.5, 8.9; **HRMS** (ESI-TOF) m/z: $[M+H]^+$ Calcd for $C_{25}H_{35}BN_3O_5$ 468.2664; Found 468.2665; **MP** 160 °C; **IR** (neat) (cm^{-1}) 2976, 2943, 2884, 1699, 1650, 1605, 1584, 1566, 1494, 1458, 1368, 1345, 1310, 1287, 1268, 1227, 1191, 1158, 1118, 1066, 971, 952, 913, 839, 777, 762, 726, 695, 670, 636, 621.

Preparation of authentic reference compounds

4-fluorobenzonitrile (**2**), 4-fluoro-1,1'-biphenyl (**3**), 1-fluoronaphthalene (**4**), 2-fluoro-1,3,5-trimethylbenzene (**5**), 1-fluoro-4-methoxybenzene (**6**), 4-fluoro-1,2-dimethoxybenzene (**7**), 1-fluoro-4-(trifluoromethyl)benzene (**8**), ethyl 4-fluorobenzoate (**9**), 1-fluoro-3-nitrobenzene (**10**), 1-fluoro-4-(methylsulfonyl)benzene (**11**), 2-(4-fluorophenyl)pyridine (**13**), 4-fluoroquinoline (**14**), 6-fluoroquinoline (**15**), 5-fluorobenzo[*d*]oxazole (**17**), 3-fluoropyridine (**20**), 5-fluoro-1*H*-indazole (**21**) and ethyl 8-fluoro-5-methyl-6-oxo-5,6-dihydro-4*H*-benzo[*f*]imidazo[1,5-*a*][1,4]diazepine-3-carboxylate (FMZ) (**26**) were purchased from commercial suppliers (Fluorochem, Sigma Aldrich, TCI, BLDpharm, Apollo Scientific, Ambeed, Manchester Organics, Biosynth, Enamine) and used as received, without further purification.

4-((4-Fluorophenyl)sulfonyl)morpholine (**12**), 5-fluoro-1-methyl-1*H*-indole (**16**), 1-(4-fluorobenzyl)-1*H*-imidazole (**22**), 2-fluorobenzo[*b*]thiophene (**23**) and 3-fluoro-5-(pyridin-2-ylethynyl)benzonitrile (**24**) were prepared exactly as described in literature procedures. [2,8-11]

Ethyl 5-methyl-6-oxo-5,6-dihydro-4*H*-benzo[*f*]imidazo[1,5-*a*][1,4]diazepine-3-carboxylate (FMZ-H)

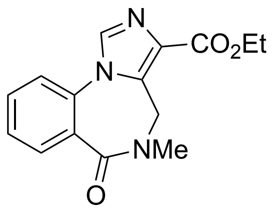

The title compound was prepared following a procedure adapted from the literature.[12]

To a two-necked round-bottomed flask charged with a magnetic stir bar was added ethyl 8-bromo-5-methyl-6-oxo-5,6-dihydro-4H-benzo[*f*]imidazo[1,5-*a*][1,4]diazepine-3-carboxylate (36 mg, 0.1 mmol) as a solid, followed by methanol (2 mL). Triethylamine (25 µL, 0.18 mmol, 1.8 equiv.) and 10% Pd/C (21 mg) were then added to the flask. The flask was evacuated under vacuum and refilled with a H_2 balloon. The reaction mixture was stirred under an H_2 atmosphere at rt for 45 minutes, after which time TLC analysis indicated full consumption of the starting material. The H_2 balloon was removed and the reaction mixture was filtered over a pad of celite, eluting with EtOAc. The volatiles were removed under reduced pressure and the crude material was purified by flash column chromatography (CH_2Cl_2 + 2% MeOH), followed by preparative thin layer chromatography (EtOAc) to afford a white solid (22 mg, 77%).

^{1}H NMR (500 MHz, $CDCl_3$) δ 8.10 – 8.05 (m, 1H), 7.89 (s, 1H), 7.68 – 7.61 (m, 1H), 7.57 – 7.50 (m, 1H), 7.43 (d, J = 7.9 Hz, 1H), 5.22 – 5.18 (m, 1H), 4.58 – 4.23 (m, 3H), 3.25 (s, 3H), 1.45 (t,

J = 7.2 Hz, 3H); **^{13}C NMR** (126 MHz, $CDCl_3$) *δ* 166.7, 163.2, 135.8, 135.1, 132.8, 132.2, 129.3, 128.9, 128.8, 122.0, 61.1, 42.5, 36.0, 14.5; **HRMS** (ESI-TOF) m/z: $[M+H]^+$ Calcd for $C_{15}H_{15}N_3O_3$ 286.1186; Found 286.1180; **MP** 160 – 162 °C. **IR** (neat) (cm^{-1}) 3106, 2933, 1723, 1637, 1564, 1499, 1450, 1429, 1399, 1376, 1346, 1296, 1262, 1237, 1200, 1166, 1107, 1067, 1026, 962, 937, 875, 844, 794, 764, 701, 684, 660.

Stability tests

Stability test of [^{18}F]FMZ precursors towards silica gel column chromatography

Stability studies were performed following a procedure adapted from the literature.[5]

Aryl boronic ester derivative of FMZ (25.0 mg) was dissolved in CH_2Cl_2 (1.0 mL) in a round-bottom flask (50 mL). Silica gel (300 mg) was added to the flask and the contents were concentrated under reduced pressure. The resulting residue was dry-loaded onto a column (18 mm diameter, containing 12.5 g of silica gel, compacted under nitrogen using acetone as eluent). The material was flushed with 50 mL of acetone, which was collected into a 100 mL round-bottom flask. The acetone was evaporated under reduced pressure and the mass of the recovered material was measured. Percentage recovery values were then calculated. The results of the stability test are summarised below (Table S1).

Table S1. Outcome of the stability test of FMZ precursors towards silica gel column chromatography.

Entry	Substrate	Recovered mass	Percentage recovery
1	FMZ-Bpin	17.4 mg	70%
2	FMZ-BEpin (**25**)	quantitative	>99%

Stability test of [^{18}F]FMZ precursors towards silica gel thin layer chromatography (TLC)

Solutions of FMZ-BEpin (**25**) and FMZ-Bpin were prepared by dissolving 1 mg of the relevant compound in 1 mL of CH_2Cl_2. 5 µL each solution were then individually spotted on 10 cm silica gel thin layer chromatography plates. The plates were ran using a 3:1 mixture (v/v) of CH_2Cl_2 and acetone. Visualisation was accomplished by means of: a) UV light (254 nm); b) staining with a solution of ceric ammonium nitrate (CAN) followed by heating; and c) staining with a solution of curcumin followed by heating (Figure S1).

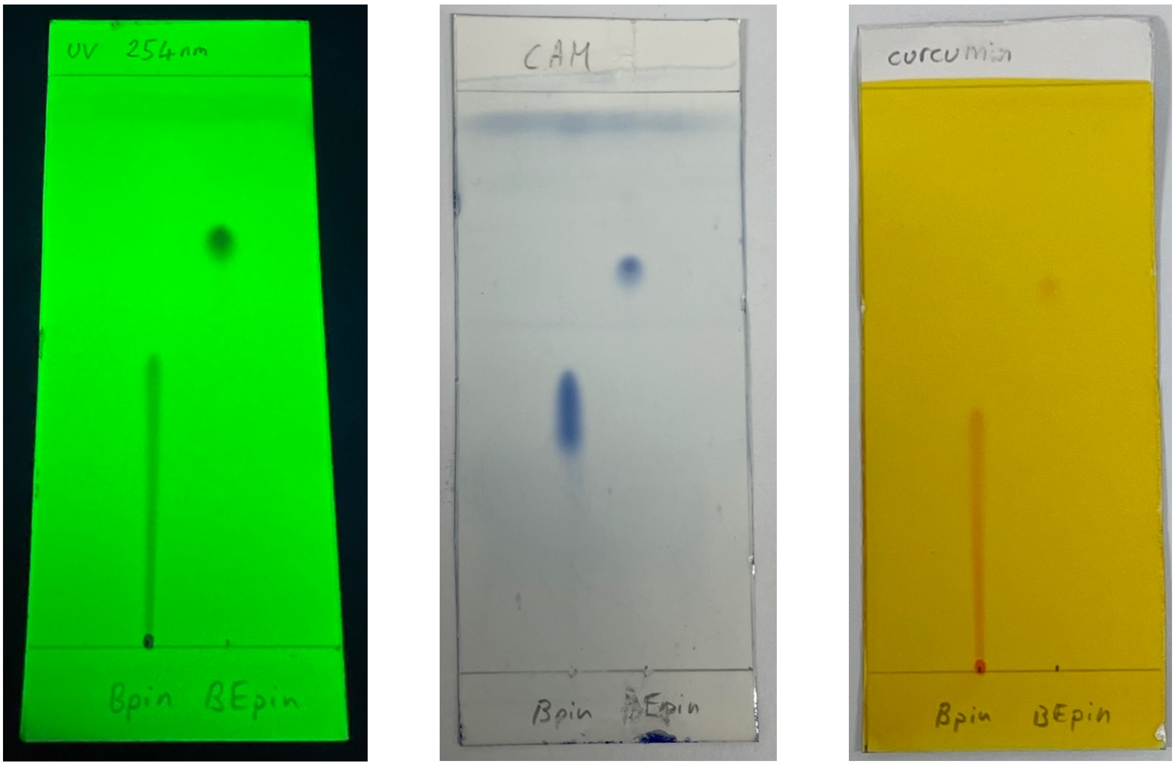

A B C

Figure S1. Silica gel thin layer chromatography (TLC) plates TLC plates of FMZ-BEpin (**25**) and FMZ-Bpin visualized by: A) UV light (254 nm), B) ceric ammonium nitrate (CAM) staining; and C) curcumin staining.

Stability test of [^{18}F]FMZ precursors towards high-performance liquid chromatography (HPLC) analysis

Solutions of FMZ-BEpin (**25**) and FMZ-Bpin were prepared by dissolving the relevant compound (1 mg) in MeCN (1 mL). The samples were then analyzed by HPLC using conditions E (injection volume = 10 µL). The corresponding UV-HPLC traces are shown below (Figures S2–S5).

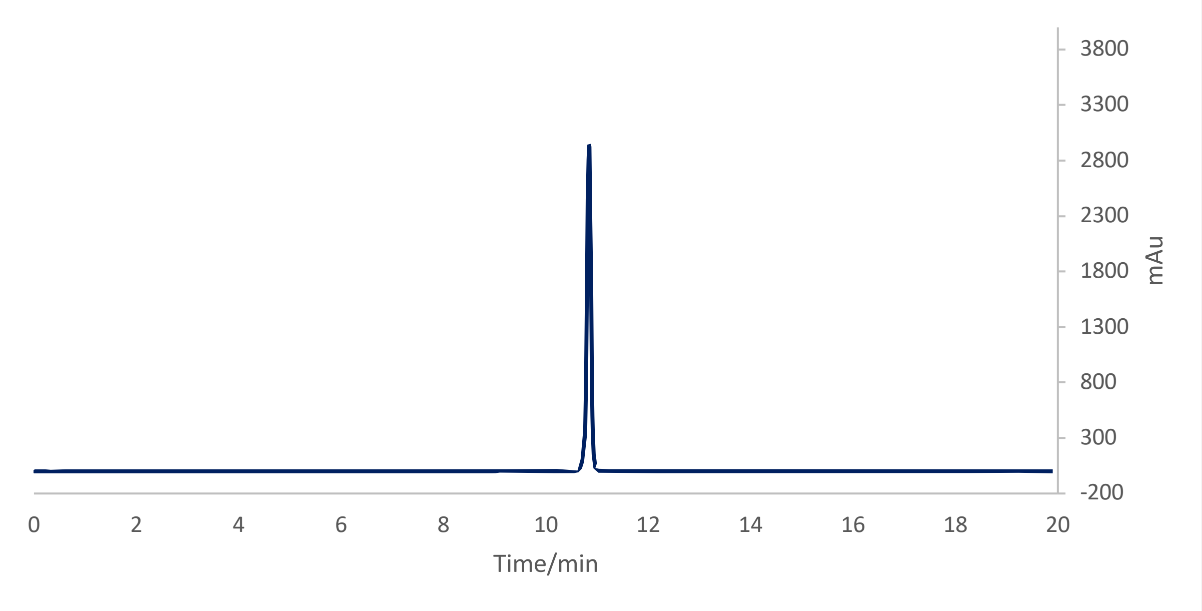

Figure S2. UV-HPLC trace of FMZ-BEpin. Analyzed using conditions E and MeCN/water as the eluent.

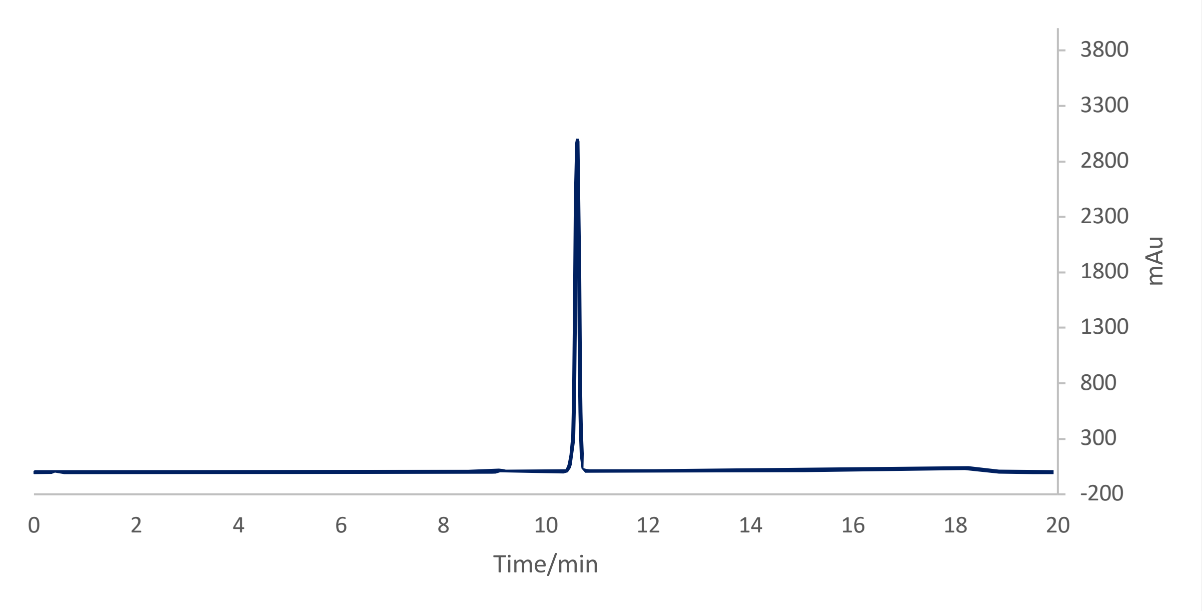

Figure S3. UV-HPLC trace of FMZ-BEpin. Analyzed using conditions E and 0.1% TFA in water/0.1% TFA in MeCN as the eluent.

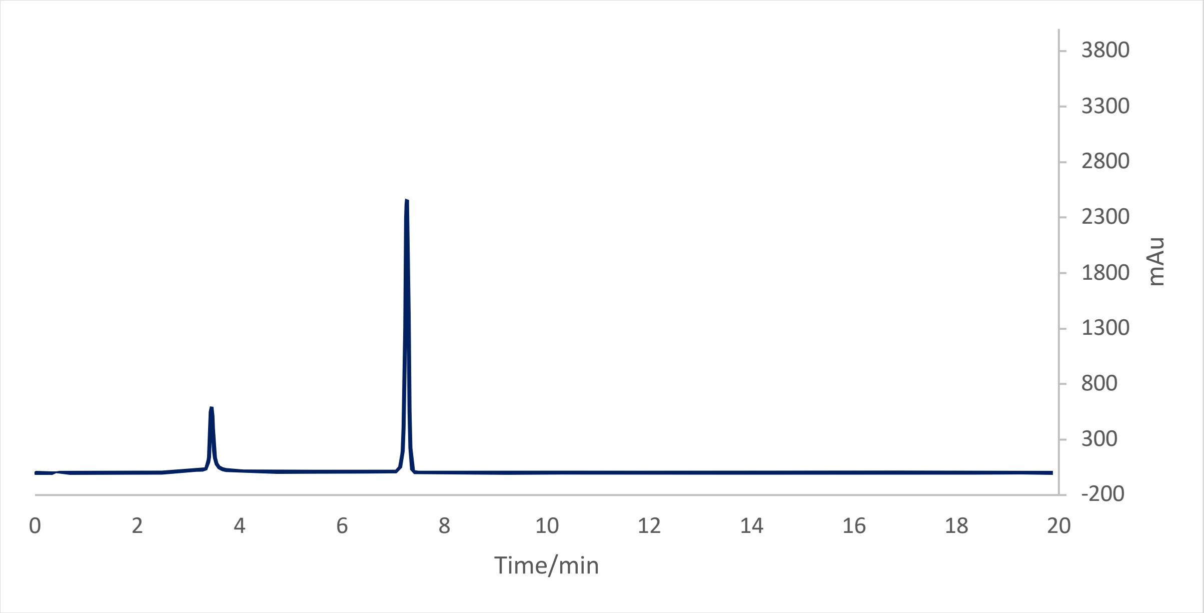

Figure S4. UV-HPLC trace of FMZ-Bpin. Analyzed using conditions E and MeCN/water as the eluent.

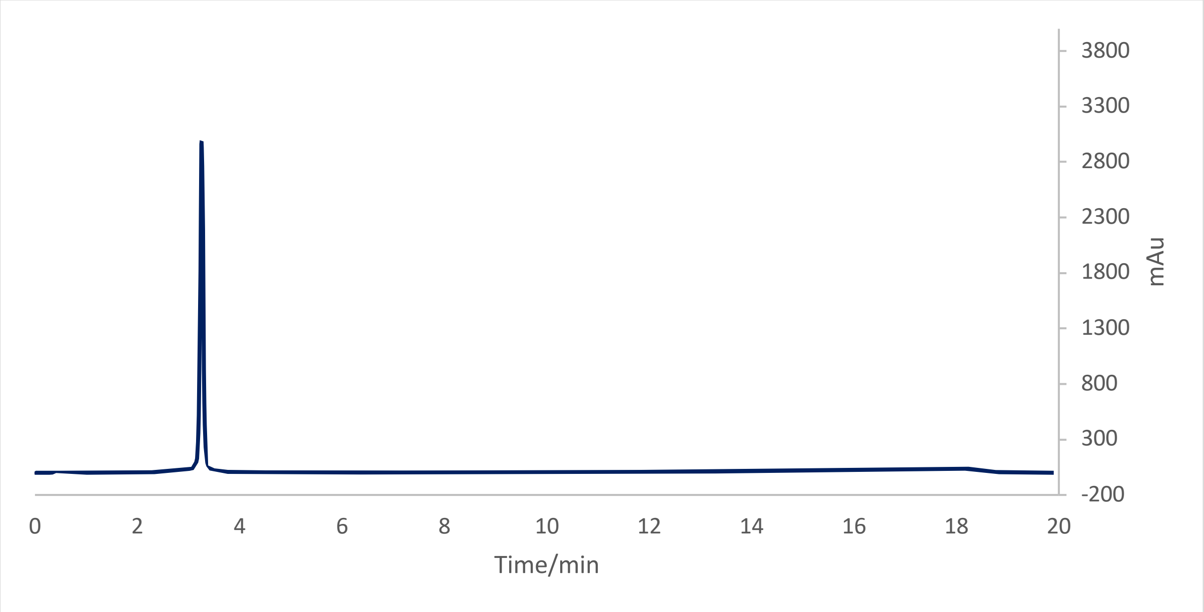

Figure S5. UV-HPLC trace of FMZ-Bpin. Analyzed using conditions E and 0.1% TFA in water/0.1% TFA in MeCN as the eluent.

Hydrolytic stability test of [^{18}F]FMZ precursors by NMR

NMR samples of FMZ-BEpin (**25**) and FMZ-BPin were prepared by dissolving 5 mg of the relevant precursor in 0.5 mL of MeCN-d_3. ^{1}H NMR spectra were then acquired (Figure S6, A and Figure S7, A). 0.25 mL of D_2O were then added to each sample, which was sonicated for 1 minute prior to the acquisition of a new set of ^{1}H NMR spectra (Figure S6, B and Figure S7, B). The above process was repeated with 1 µL of TFA-*d* (Figure S6, C and Figure S7, C).

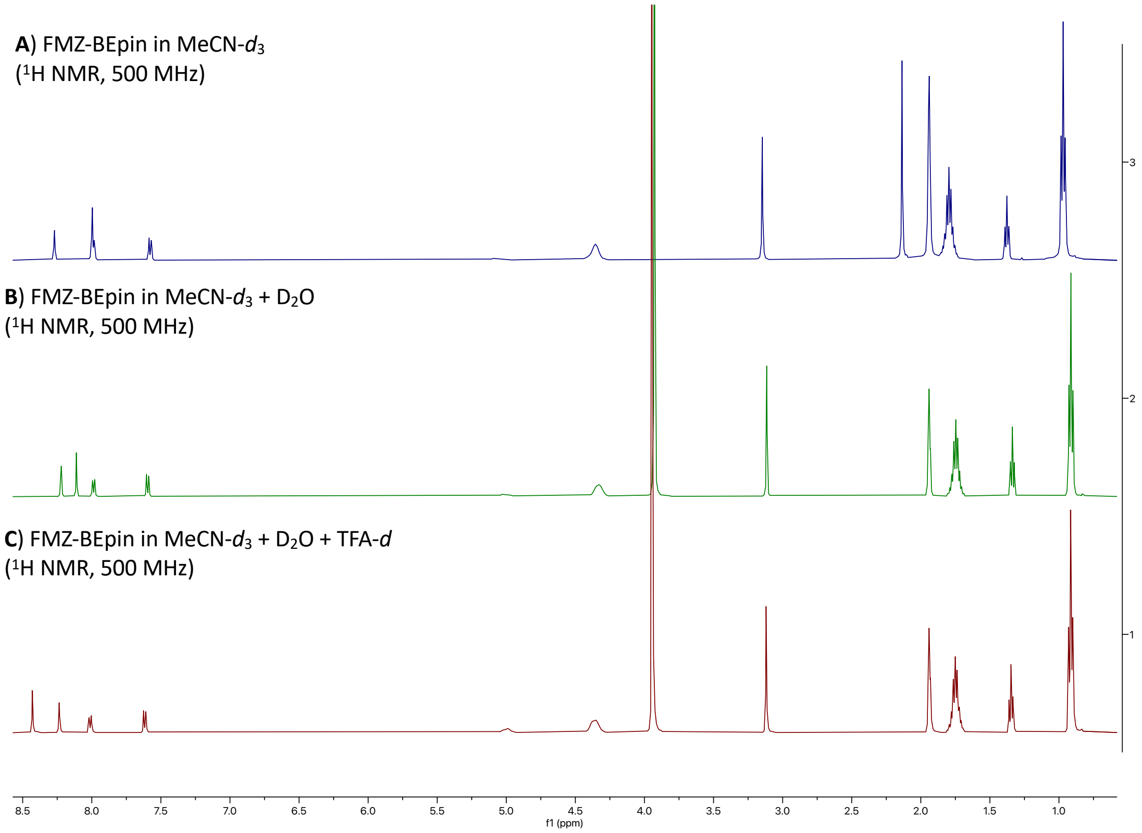

Figure S6. ^{1}H NMR spectra of FMZ-BEpin in MeCN-d_3 with: A) no additives; B) D_2O; c) D_2O and TFA-*d*. No formation of Epin (3,4-diethylhexane-3,4-diol) observed in all cases [Epin: **^{1}H NMR** (500 MHz, MeCN-d_3/D_2O) δ 1.61 – 1.45 (m, 8H), 0.84 (t, J = 7.5 Hz, 12H)].

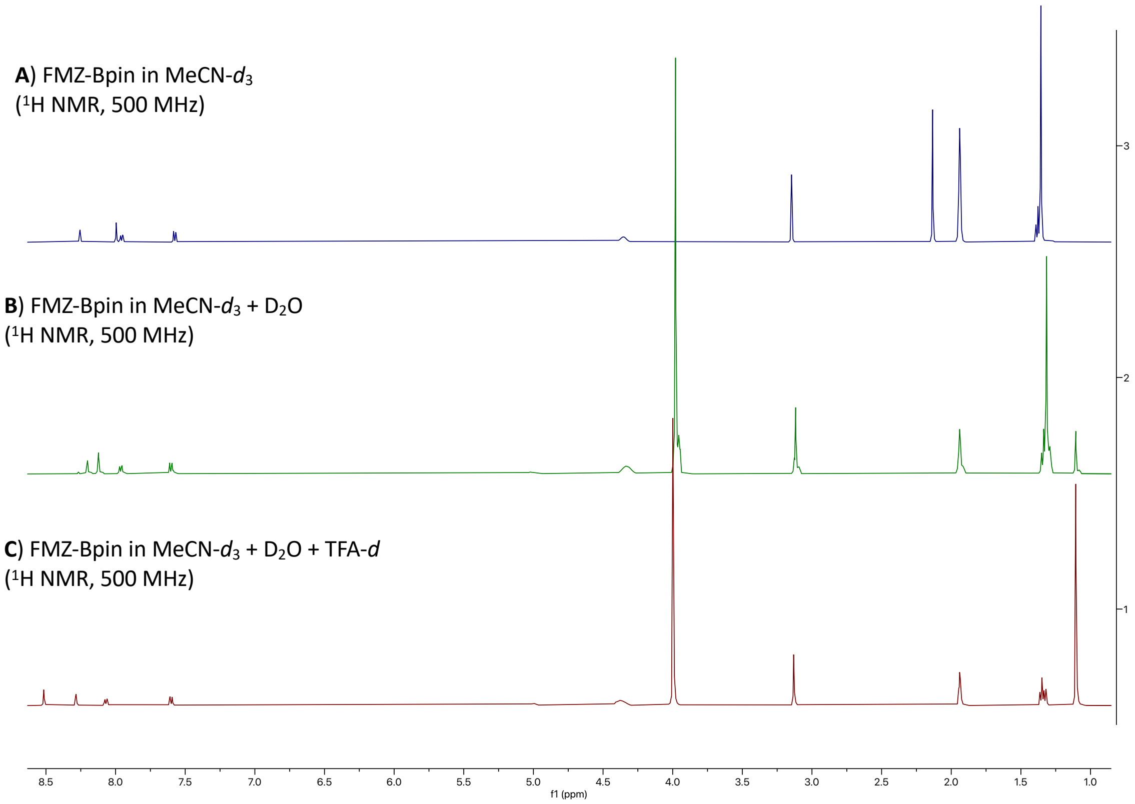

Figure S7. ^{1}H NMR spectra of FMZ-Bpin in MeCN-d_3 with: A) no additives; B) D_2O; c) D_2O and TFA-*d*. Formation of pinacol observed (B/C) [Pinacol: **^{1}H NMR** (500 MHz, MeCN-d_3/D_2O) δ 1.11 ppm (12H, s)].

Radiochemistry

***Caution!** Due to the spontaneous fission and β^+ and γ particle emission from fluorine-18, the radioisotope sample represents a serious health hazard. All studies with fluorine-18 were conducted in a radiation laboratory equipped with appropriate lead-shielded hot cells.*

HPLC conditions

Analytical HPLC runs were performed either with an Agilent 1200 equipped with a UV detector and LabLogic gamma-RAM Model 4 detector (approximate radio-UV detector offset = +0.1 min) or a LabLogic FlowRAM detector (approximate radio-UV detector offset = -0.1 min) (Conditions A-E).

Semi-preparative purification of radiolabelled products was achieved using the integrated HPLC system (with UV and radio detectors) of a Trasis AllInOne synthesizer (Conditions F).

***Conditions A:** Analytical*

Flow rate = 1.0 mL/min
Temperature = 25 °C
Wavelength = 220 nm (unless otherwise specified)
Column: Agilent C18 Eclipse Plus 80 Å 150 x 4.6 mm LC column
HPLC gradient: water/MeCN

- 0-1 min (25% MeCN) isocratic
- 1-10 min (25% MeCN to 95% MeCN) linear increase
- 10-16 min (95% MeCN) isocratic
- 16-18 min (95% MeCN to 25% MeCN) linear decrease
- 18-20 min (25% MeCN) isocratic

***Conditions B:** Analytical*

Flow rate = 1.0 mL/min
Temperature = 25 °C
Wavelength = 220 nm (unless otherwise specified)
Column: Agilent C18 Eclipse Plus 80 Å 150 x 4.6 mm LC column
HPLC gradient: water/MeCN

- 0-1 min (25% MeCN) isocratic
- 1-8 min (25% MeCN to 95% MeCN) linear increase
- 8-13 min (95% MeCN) isocratic
- 13-14 min (95% MeCN to 25% MeCN) linear decrease
- 14-15 min (25% MeCN) isocratic

***Conditions C:** Analytical*

Flow rate = 1.0 mL/min

Temperature = 25 °C
Wavelength = 220 nm (unless otherwise specified)
Column: Agilent C18 Eclipse Plus 80 Å 150 x 4.6 mm LC column
HPLC gradient: water/MeCN

- 0-1 min (5% MeCN) isocratic
- 1-8 min (5% MeCN to 95% MeCN) linear increase
- 8-13 min (95% MeCN) isocratic
- 13-14 min (95% MeCN to 5% MeCN) linear decrease
- 14-15 min (5% MeCN) isocratic

Conditions D: *Analytical*

Flow rate = 2.0 mL/min
Temperature = 25 °C
Wavelength = 246 nm
Column: Chromolith® Performance RP-18 endcapped 100 x 3.0 mm LC column
HPLC gradient: 0.1% TFA in water/0.1% TFA in MeCN

- 0-5 min (5% MeCN to 30% MeCN) linear increase
- 5-5.5 min (30% MeCN to 90% MeCN) linear increase
- 5.5-7.5 min (90% MeCN) isocratic
- 7.5-8 min (90% MeCN to 5% MeCN) linear decrease
- 8-15 min (25% MeCN) isocratic

Conditions E: *Analytical*

Flow rate = 2.0 mL/min
Temperature = 25 °C
Wavelength = 220 nm
Column: Chromolith® Performance RP-18 endcapped 100 x 3.0 mm LC column
HPLC gradient: water/MeCN or : 0.1% TFA in water/0.1% TFA in MeCN

- 0-17.5 min (5% MeCN to 90% MeCN) linear increase
- 17.5-18.5 min (90% MeCN to 5% MeCN) linear decrease
- 18.5-20 min (5% MeCN) isocratic

Conditions F: *Semi-preparative purification*

Flow rate = 4.0 mL/min
Temperature = room temperature
Wavelength = 254 nm
Column: Phenomenex Luna 5 µm C18(2) 100 Å LC column (250 x 10 mm)
Isocratic: 25% EtOH in 10 nM aqueous phosphate buffer

Manual radiofluorination of aryl boronic esters

General procedure for the ^{18}F-fluorination of aryl boronic esters

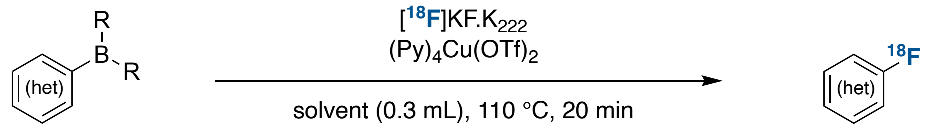

Scheme S1. Radiofluorination of aryl boronic esters with [^{18}F]KF.

[^{18}F]KF elution and drying. Using a Trasis AllinOne automated synthesiser, [^{18}F]fluoride was separated from ^{18}O-enriched-water using an anion exchange cartridge (Waters Sep-Pak AccellPlus QMA Carbonate Plus Light Cartridge, activated with H_2O (10.0 mL) prior to use) and released with a solution of K_{222} (7.5 mg) and K_2CO_3 (1.5 mg) in MeCN/H_2O (0.75 mL, 4:1, v/v). The solution was dried over a period of 20 min by azeotropic drying using MeCN (3 x 0.7 mL) under a flow of N_2 at 110 °C.

Manual ^{18}F-fluorination. To an oven-dried 3 mL glass v-vial equipped with a magnetic stir bar and sealed with a septum was added the relevant aryl boron reagent, $Cu(OTf)_2py_4$ and solvent (300 µL). To the vial was then added an aliquot of the [^{18}F]KF.K_{222} solution (5-20 MBq) in MeCN (for optimisation entries) or DMI (for scope entries) (approx. 20 µL). The reaction vial was purged with air (20 mL) over the course of 20 seconds and the reaction mixture was stirred at 110 °C in an aluminium heating block for 20 minutes. The reaction mixture was cooled to rt and then diluted with MeCN/H_2O (200 µL, 3:1 v/v). An aliquot of this mixture was subsequently analyzed by radioHPLC (HPLC conditions A–C) for radiochemical yield (RCY) and product identity. The RCY was determined by integration of the desired ^{18}F-product relative to the total peak area for all radioactive species observed. Identity of the ^{18}F-product was confirmed by matching of the UV-HPLC retention time of an authentic ^{19}F-reference.

Comparison of different aryl boron reagents under copper-mediated radiofluorination.

Scheme S2. Copper-mediated radiofluorination of aryl boron reagents derived from benzonitrile with [^{18}F]KF.

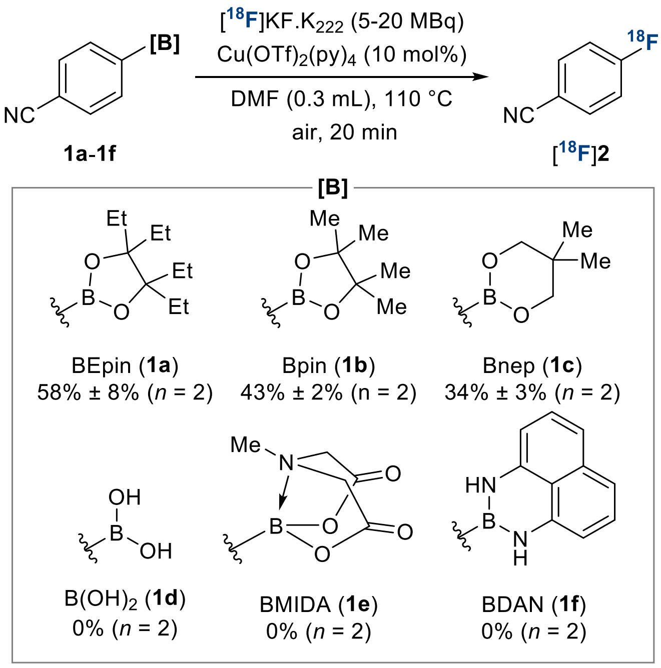

Radiofluorination experiments performed according to the general procedure. RCY determined by radio-HPLC analysis of the crude reaction mixture.

Optimisation data

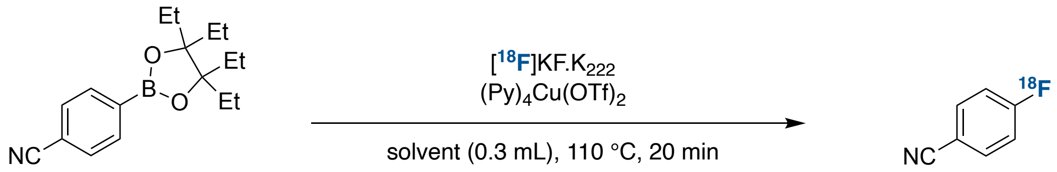

Table S2. Optimisation of the radiofluorination of 4-(4,4,5,5-tetraethyl-1,3,2-dioxaborolan-2-yl)benzonitrile (**1a**) with [^{18}F]KF.

Entry	Loading of **1a**	Equivalents of $Cu(OTf)_2py_4$	Solvent	RCY[a]
1	0.06 mmol	0.1	DMF	58% ± 8% (*n* = 2)
2	0.06 mmol	0.1	DMA	51% (*n* = 1)
3	0.06 mmol	0.1	DMI	81% (*n* = 1)
4	0.06 mmol	0.1	NMP	0% (*n* = 1)
5	0.06 mmol	0.1	DMSO	4% (*n* = 1)
6	0.06 mmol	0.1	MeCN	10% (*n* = 1)
7	0.06 mmol	0.5	DMF	72% (*n* = 1)
8	0.06 mmol	0.5	DMI	97% (*n* = 1)
9	0.06 mmol	1.0	DMI	96% (*n* = 1)
10	0.02 mmol	1.0	DMI	95% ± 2% (*n* = 2)
11[b]	0.02 mmol	-	DMI	80% (*n* = 1)

Radiofluorination experiments performed according to the general procedure. **[a]** RCY determined by radio-HPLC analysis of the crude reaction mixture. **[b]** $Cu(OTf)_2$ (0.02 mmol), pyridine (40 µL) added in place of $Cu(OTf)_2py_4$.

Automated radiofluorination of FMZ-BEpin using a Trasis AllinOne radiosynthesizer

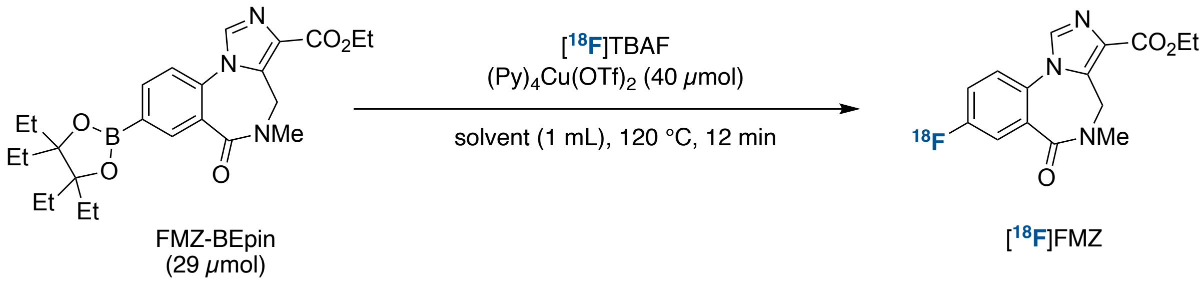

Scheme S3. Radiofluorination of FMZ-BEpin with [^{18}F]TBAF.

The automated radiosynthesis of [^{18}F]**FMZ** was performed exactly as described in a literature procedure using a Trasis AllInOne synthesizer using an automated program and pre-built cassette, with a single modification, in which FMZ-BEpin was used in place of FMZ-Bpin.[13] For all radiosyntheses, a starting activity of 25 GBq [^{18}F]fluoride was used.

The following reagents were added to the cassette for the radiosynthesis:

- 4 mL clear glass vial in slot 2 was charged with $TBAHCO_3$ (8 mg), H_2O (0.1 mL) and MeCN (0.4 mL).
- 4 mL amber glass vial in slot 8 was charged with FMZ-BEpin (14 mg, 29 µmol) and DMA solvent (0.5 mL).
- 4 mL clear glass vial in slot 10 was charged with $Cu(OTf)_2py_4$ (27 mg, 40 µmol) and DMA solvent (0.5 mL).
- 20 mL clear glass vial in slot 12 was charged with EtOH (10 mL).
- 20 mL clear glass vial in slot 15 was charged with sodium ascorbate (125 mg).

At the end of the radiosynthesis, the product vial was disconnected from the synthesizer and transferred to the dose calibrator, where the activity of the collected product was measured. An aliquot (ca. 20 MBq) of the isolated product was analyzed by radio-HPLC to determine molar activity. HPLC analysis was performed under HPLC conditions D. The molar activity was determined, as follows. The same sample was injected in triplicate, with the injected activity and time of injection recorded. The UV response corresponding to the desired radiofluorinated product was then integrated, to give the amount of non-radioactive product that was detected. Molar activity was then calculated. The concentrations of known impurities (FMZ-H and FMZ-OH) were determined in the same way.

Radiosynthesis data are summarized below (Table S3).

Table S3. Radiosynthesis of [^{18}F]FMZ.

Entry	Solvent	Starting activity	AY	Synthesis time	RCP	A_m
1	DMA	25 GBq	2.07 GBq	51 min	>99%	202 GBq/µmol
2	DMA	25 GBq	1.82 GBq	52 min	>99%	236 GBq/µmol
3	DMI	25 GBq	3.36 GBq	51 min	>99%	145 GBq/µmol

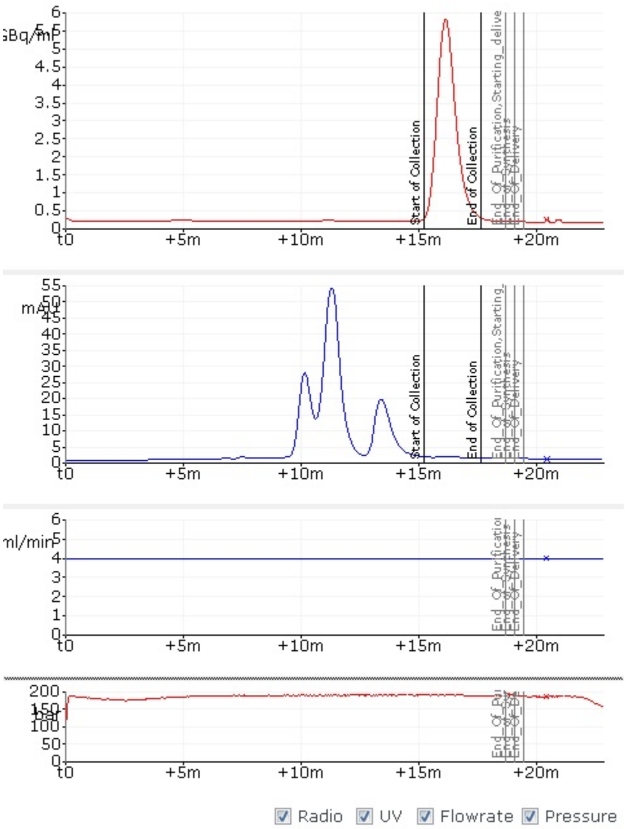

Figure S8. Semi-preparative HPLC chromatogram obtained during the purification of [^{18}F]FMZ using DMA as solvent (HPLC conditions F). From top to bottom: radioHPLC trace (GBq/mL), UV-HPLC trace (mAuS), flowrate (mL/min), pressure (bar).

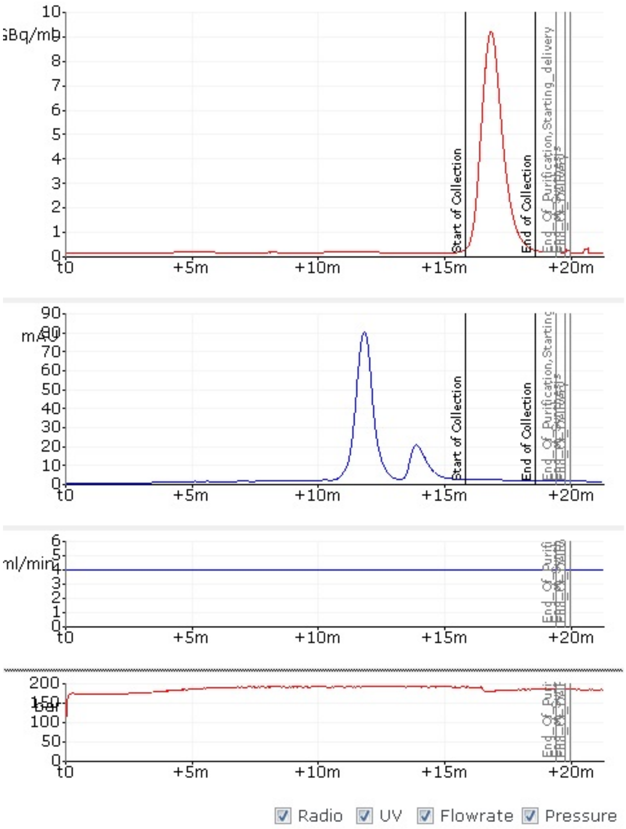

Figure S9. Semi-preparative HPLC chromatogram obtained during the purification of [^{18}F]FMZ using DMI as solvent (HPLC conditions F). From top to bottom: radioHPLC trace (GBq/mL), UV-HPLC trace (mAuS), flowrate (mL/min), pressure (bar).

Quality control of [^{18}F]FMZ

Table S4. Acceptance criteria for formulated [^{18}F]FMZ, set according to the European Pharmacopoeia and ICH Q3D(R1) guidelines.[13]

Entry	Control	Acceptance criteria
1	Appearance	Clear and colorless
2	pH	4.5 to 8.5
3	Radiochemical purity	[^{18}F]FMZ $\geq$ 95%<br>[^{18}F]fluoride $\leq$ 5%
4	Chemical purity	^{19}F-FMZ $\leq$ 50 µg/V<br>TBA-HCO_3 $\leq$ 2.6 µg/V<br>Cu^{II} $\leq$ 340 µg/V

V = volume of the formulated radiofluorinated product (25 mL).

Appearance

The content of the final product vial was visually assessed behind appropriate shielding. All final product solutions were clear, colourless, and free of visible particulate matter.

Identity

Identity of [^{18}F]FMZ was confirmed by matching of the UV-HPLC retention time of the authentic ^{19}F-reference FMZ (Figures S10 and S11).

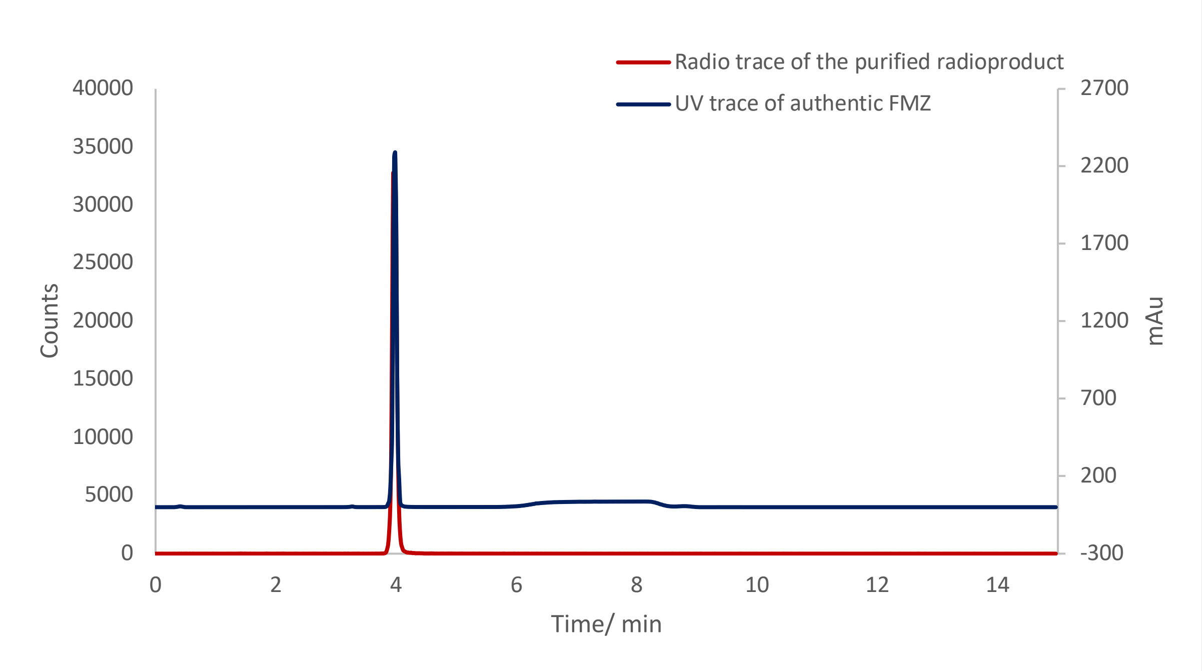

Figure S10. Overlay of radio-HPLC trace for isolated [^{18}F]FMZ using DMA as solvent and UV-HPLC trace for isolated [^{18}F]FMZ (HPLC conditions D).

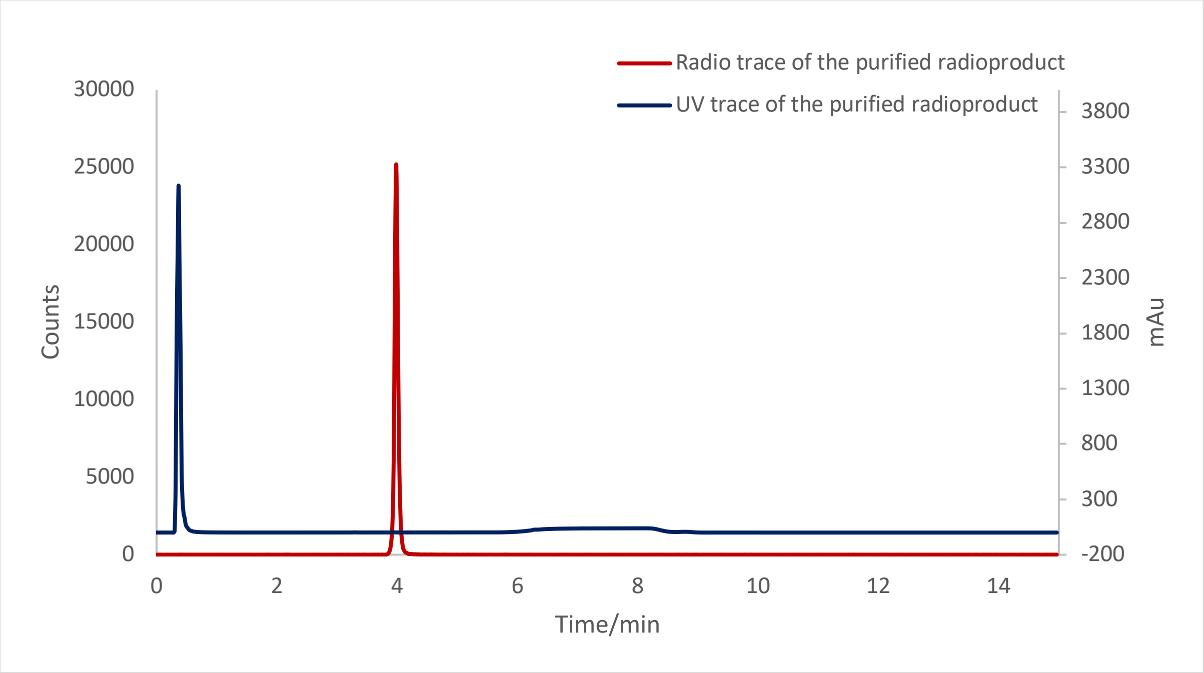

Figure S11. Overlay of radio-HPLC trace for isolated [^{18}F]FMZ using DMA as solvent and UV-HPLC trace for isolated [^{18}F]FMZ (HPLC conditions D). *Note*: The most intense UV peak at approx. 0.5 min is due to the presence of sodium ascorbate in the formulation.

pH

The pH of the final product solutions was measured using colorimetric pH paper strips and was found to fall between 6 and 7 (Figure S12)

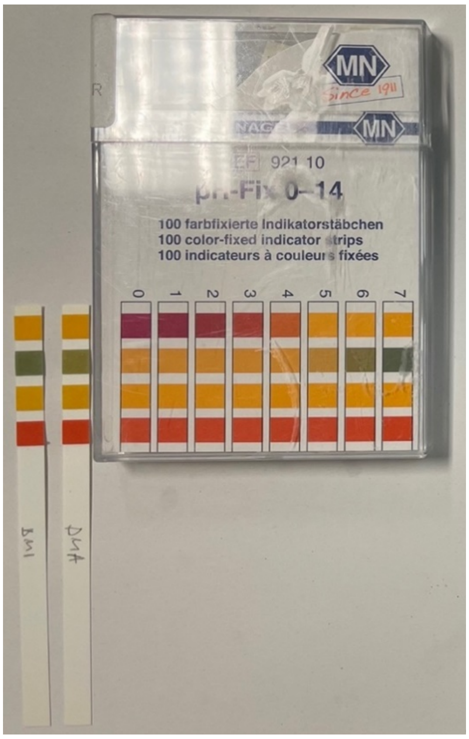

Figure S12. Determination of the pH of the final solutions of [^{18}F]FMZ using colorimetric pH paper strips.

Radiochemical purity

The radiochemical purity was assessed by radio-HPLC (Figure S13 and S15) and radio-TLC (Figure S14 and S16). In all cases, RCP was found to be greater than 99%.

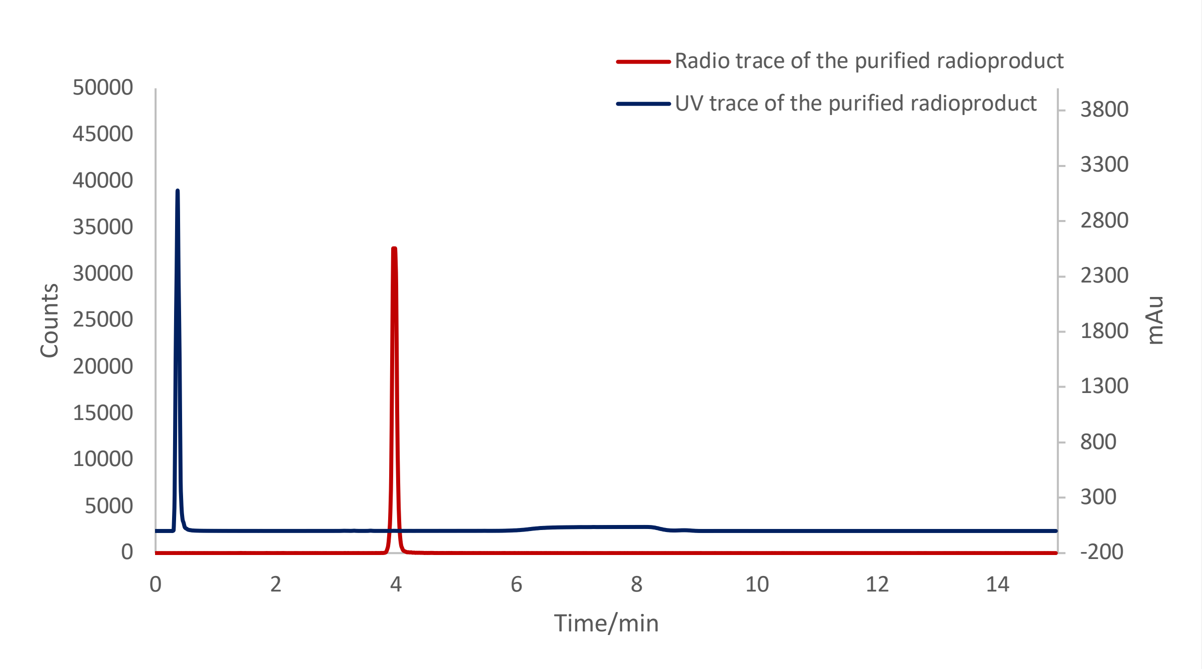

Figure S13. Overlay of radio-HPLC trace for isolated [^{18}F]FMZ using DMA as solvent and UV-HPLC trace for authentic FMZ reference (HPLC conditions D). *Note*: The most intense UV peak at approx. 0.5 min is due to the presence of sodium ascorbate in the formulation.

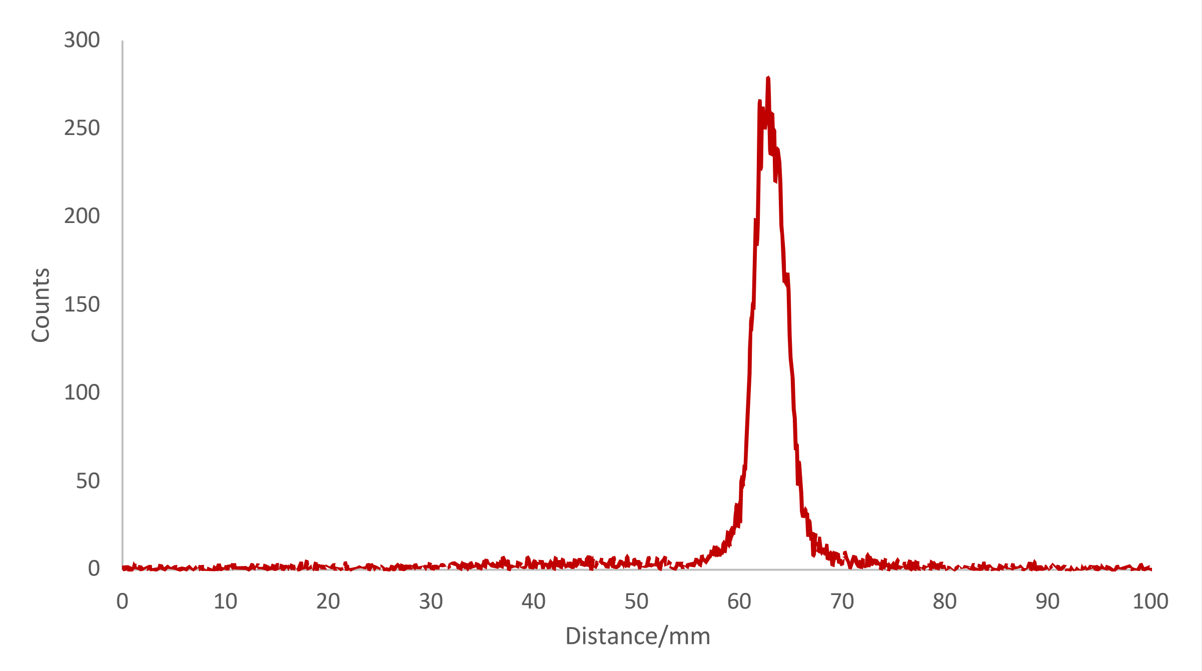

Figure S14. Radio-TLC for isolated [^{18}F]FMZ using DMA as solvent. Eluent = 5% MeOH in CH_2Cl_2, 100 mm silica gel TLC plates (Merck Kieselgel 60 F254). Solvent front = 900 mm; retention factor (R_f = 0.78).

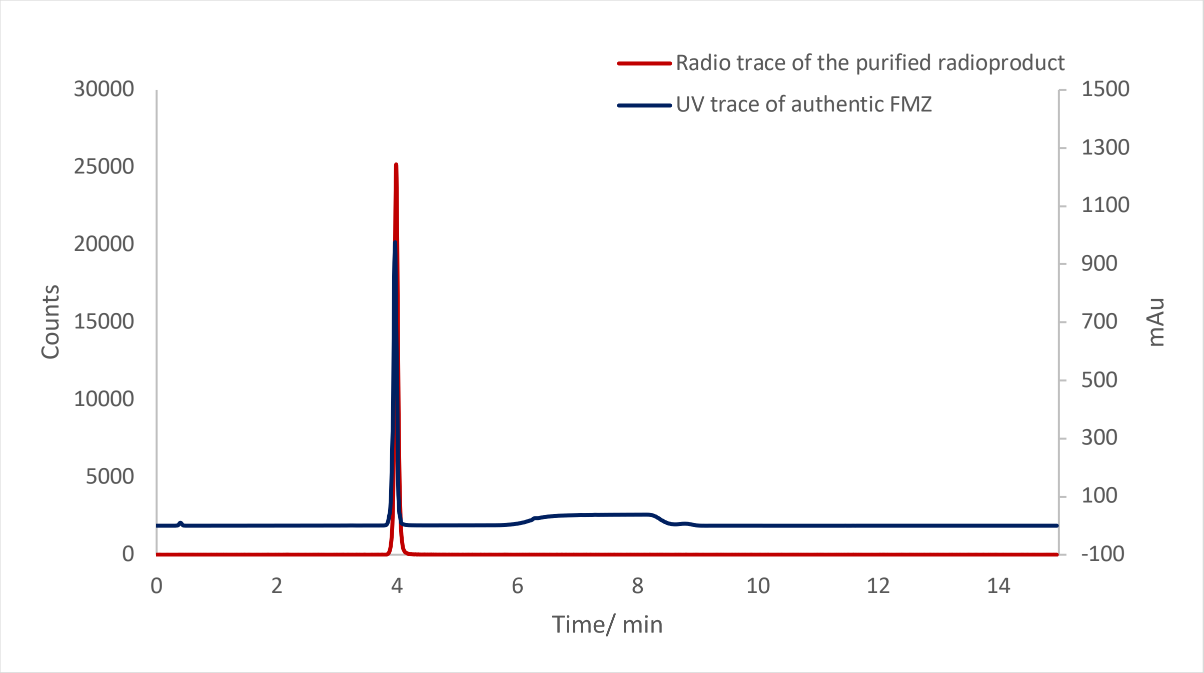

Figure S15. Overlay of radio-HPLC trace for isolated [^{18}F]FMZ using DMI as solvent and UV-HPLC trace for authentic FMZ reference (HPLC conditions D). Solvent front = 900 mm; retention factor (R_f = 0.73).

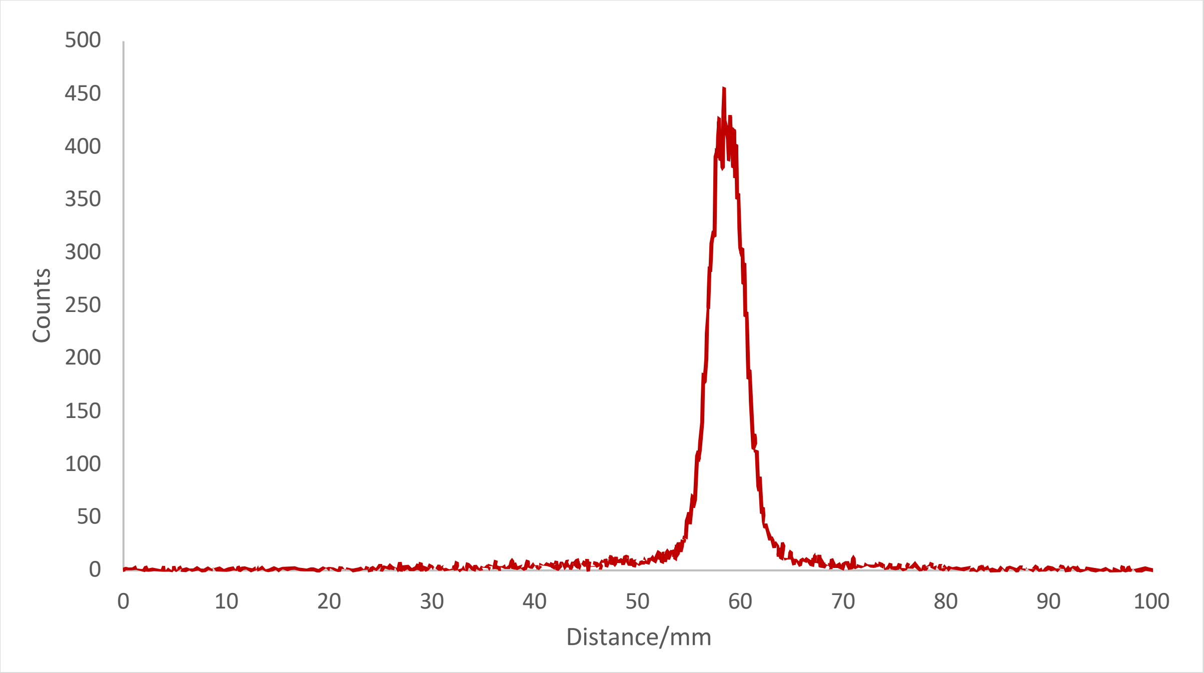

Figure S16. Radio-TLC for isolated [^{18}F]FMZ using DMI as solvent. Eluent = 5% MeOH in CH_2Cl_2, 100 mm silica gel TLC plates (Merck Kieselgel 60 F254).

Chemical purity

The chemical purity of the final product was assessed by quantitative HPLC. The three main non-radioactive impurities identified in the final formulation were:

1. ^{19}F-Flumazenil
2. Defluoro 8-hydroxy flumazenil (otherwise known as USP Flumazenil impurity B, abbreviated as FMZ-OH)
3. Desfluoro flumazenil, abbreviated as FMZ-H or HMZ

Calibration curves for all three compounds were obtained using authentic standards (Figures S17–S19).

Procedure for the calibration curve of FMZ. A calibration curve for authentic reference FMZ was recorded by preparing samples of a range of concentrations by serial dilution, starting with a solution of FMZ (2 mg) in MeCN (1.0 mL) (Figure S17). These were injected onto an HPLC (5 µL injection volume from a 1.0 mL stock, HPLC conditions D) and the UV response was measured by integrating the peak of interest.

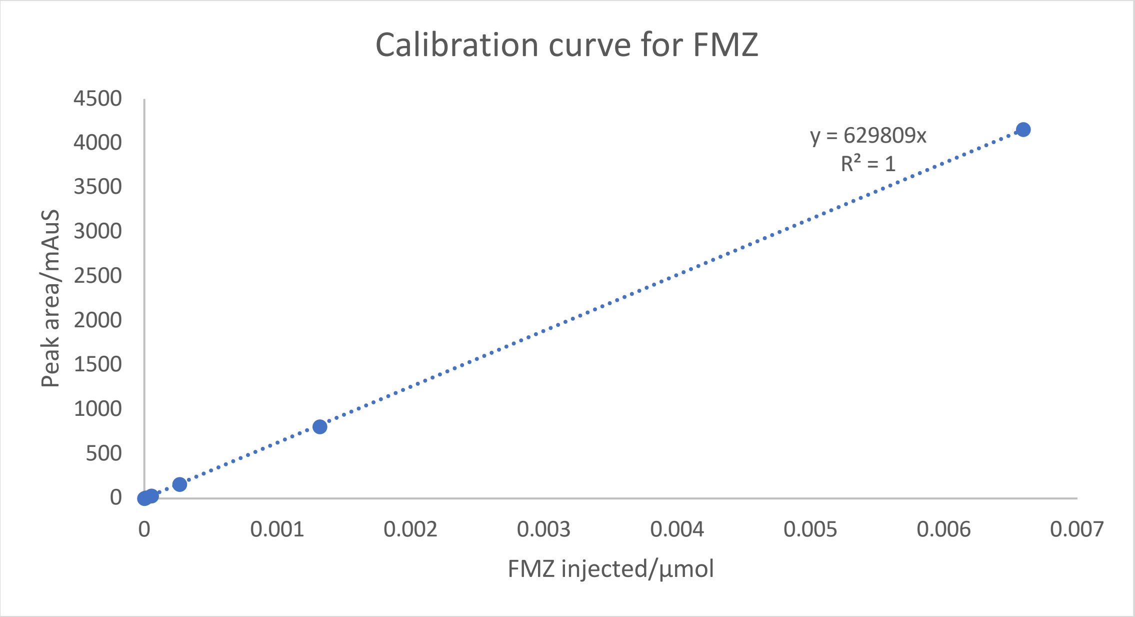

Figure S17. HPLC calibration curve for authentic reference FMZ.

Procedure for the calibration curve of FMZ-OH (European Pharmacopoeia Reference Standard)**.** A calibration curve for authentic reference FMZ-OH was recorded by preparing samples of a range of concentrations by serial dilution, starting with a solution of FMZ-OH (1 mg) in MeCN/DMSO (1.1 mL, 10:1 v/v) (Figure S18). These were injected onto an HPLC (5 µL injection volume from a 1.0 mL stock, HPLC conditions D) and the UV response was measured by integrating the peak of interest.

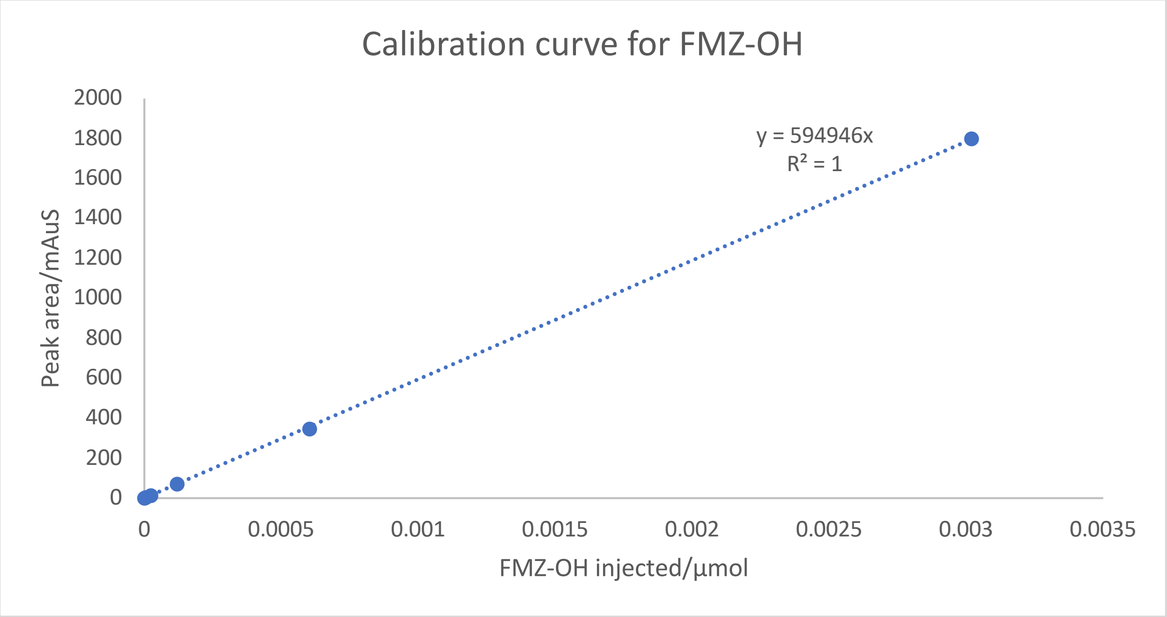

Figure S18. HPLC calibration curve for authentic reference FMZ-OH.

Procedure for the calibration curve of FMZ-H. A calibration curve for authentic reference **FMZ** was recorded by preparing samples of a range of concentrations by serial dilution, starting with a solution of **FMZ-H** (1 mg) in MeCN (1.0 mL) (Figure S19). These were injected onto an HPLC (5 µL injection volume from a 1.0 mL stock, HPLC conditions D) and the UV response was measured by integrating the peak of interest.

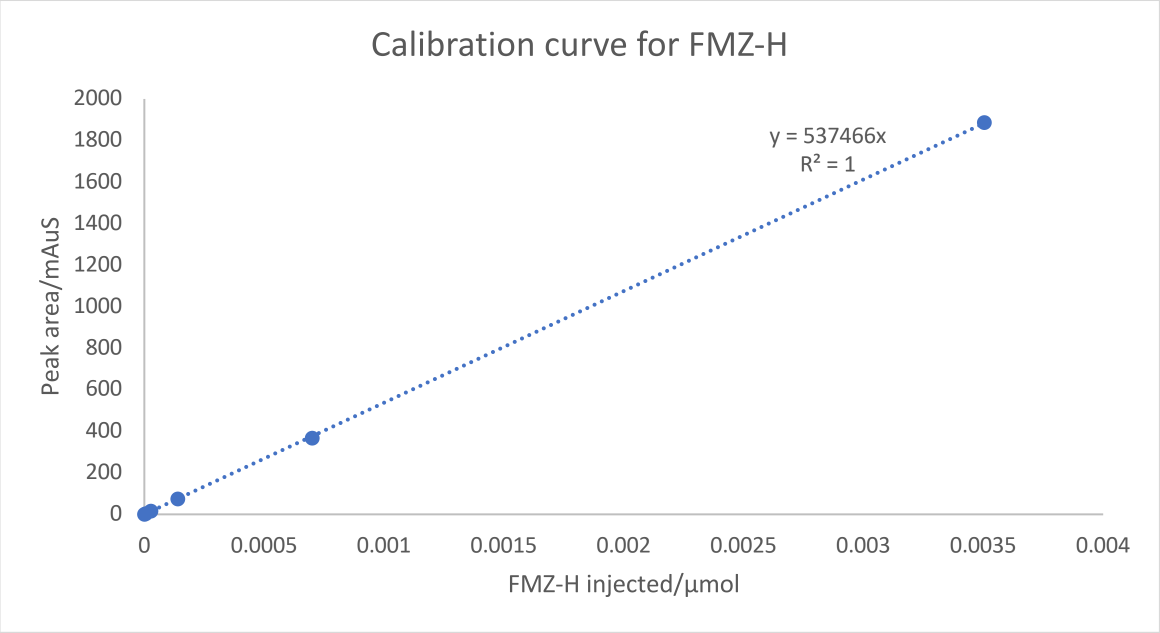

Figure S19. HPLC calibration curve for authentic reference FMZ-H.

Table S5. Calculation for **FMZ-OH** using DMA as the reaction solvent.

**Run 1**			
**Measurement**	**Peak area (mAu*s)**	**FMZ-OH injected (μg)**	**FMZ-OH (μg/V)**
1	4.8	2.43×10^{-3}	12.15
2	4.9	2.48×10^{-3}	12.40
3	3.3	1.67×10^{-3}	8.35
		**Average**	**11.0 ± 1.9**
**Run 2**			
**Measurement**	**Peak area (mAu*s)**	**FMZ-OH injected (μg)**	**FMZ-OH (μg/V)**
1	2.5	1.27×10^{-3}	6.33
2	3.2	1.62×10^{-3}	8.10
3	5.9	2.99×10^{-3}	14.95
		**Average**	**9.8 ± 3.7**

V = volume of the formulated radiofluorinated product (25 mL).

Table S6. Calculation for **FMZ-H** using DMA as the reaction solvent.

**Run 1**			
**Measurement**	**Peak area (mAu*s)**	**FMZ-H injected (μg)**	**FMZ-H (μg/V)**
1	5.1	2.71×10^{-3}	13.55
2	5.3	2.81×10^{-3}	14.08
3	4.7	2.50×10^{-3}	12.48
		**Average**	**13.4 ± 0.7**
**Run 2**			
**Measurement**	**Peak area (mAu*s)**	**FMZ-H injected (μg)**	**FMZ-H (μg/V)**
1	0.4	2.12×10^{-4}	1.06
2	0.3	1.59×10^{-4}	0.80
3	0.5	2.65×10^{-4}	1.33
		**Average**	**1.1 ± 0.2**

V = volume of the formulated radiofluorinated product (25 mL).

Table S7. Calculation for **FMZ-OH** using DMI as the reaction solvent.

**Run 1**			
**Measurement**	**Peak area (mAu*s)**	**FMZ-H injected (µg)**	**FMZ-OH (µg/V)**
1	7.8	3.95×10^{-3}	19.75
2	4.3	2.18×10^{-3}	10.90
3	5.3	2.68×10^{-3}	13.43
		**Average**	**14.7 ± 3.7**

V = volume of the formulated radiofluorinated product (25 mL).

Table S8. Calculation for **FMZ-H** using DMI as the reaction solvent.

**Run 1**			
**Measurement**	**Peak area (mAu*s)**	**FMZ-H injected (µg)**	**FMZ-H (µg/V)**
1	0.1	5.31×10^{-5}	0.27
2	0.1	5.31×10^{-5}	0.27
3	0.1	5.31×10^{-5}	0.27
		**Average**	**0.3 ± 0**

V = volume of the formulated radiofluorinated product (25 mL).

Semi-quantitative assessment of *tert*-butyl ammonium (TBA) residuals in the formulated [^{18}F]FMZ sample was carried out using a published TLC method using iodine staining (Figure S20).[13] 5 µL of the final product solution were spotted onto a 10 cm silica gel TLC plate. The plates were ran using a 9:1 mixture (v/v) of MeOH and aqueous NH_4OH. After 30 minutes, the plate was exposed to iodine vapor for 1 minute.

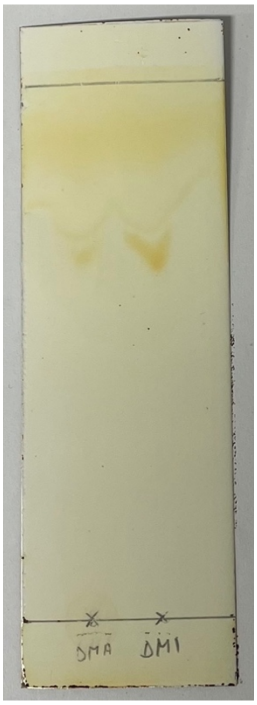

Figure S20. Determination of residual *tert*-butyl ammonium (TBA) in the final solutions of [^{18}F]FMZ using silica gel thin layer chromatography and iodine staining.

Copper content

Semi-quantitative assessment of residual copper content in the final solutions of [^{18}F]FMZ was performed using test strips specific to Cu(I)/(II) (Figure S21). In all cases, residual Cu(I)/(II) was found to be ≤10 mg/L.

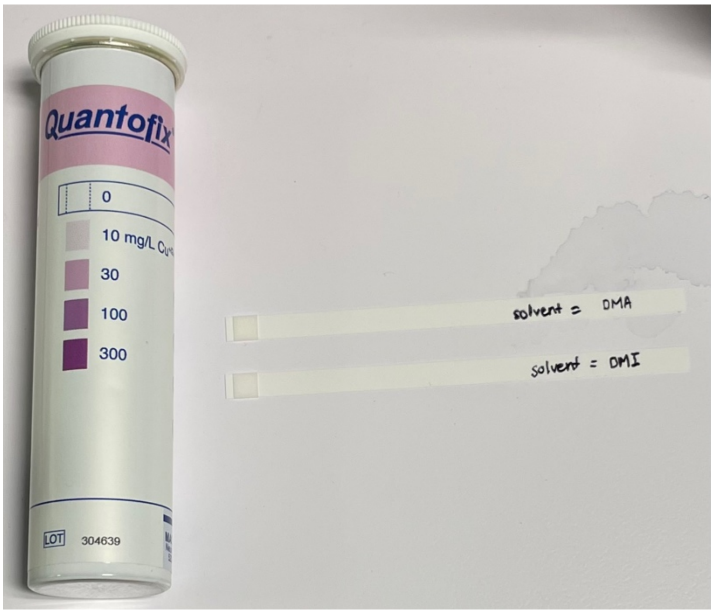

Figure S21. Determination of residual copper content in the final solutions of [^{18}F]FMZ using Quantofix Cu(I)/(II) semiquantitative test strips.

Molar activity

An aliquot (ca. 20 MBq) of the isolated product was analyzed by radio-HPLC to determine molar activity. HPLC analysis was performed under HPLC conditions D. The same sample was injected in triplicate, with the injected activity and time of injection recorded. The UV response corresponding to the desired radiofluorinated product was then integrated, to give the amount of non-radioactive product that was detected. Molar activity was then calculated. These data are summarized below (Tables S9 and S10).

Table S9. Molar activity calculation for [^{18}F]FMZ using DMA as the reaction solvent.

**Run 1**				
**Measurement**	**Activity injected (MBq, d.c.)**	**Peak area (mAu*s)**	**FMZ injected (µmol)**	***A_m* (GBq/µmol)**
1	0.93	3.0	4.76×10^{-6}	195
2	0.96	2.6	4.13×10^{-6}	233
3	0.79	2.8	4.45×10^{-6}	178
			**Average**	**202 ± 23**
**Run 2**				
**Measurement**	**Activity injected (MBq, d.c.)**	**Peak area (mAu*s)**	**FMZ injected (µmol)**	***A_m* (GBq/µmol)**
1	0.55	1.5	2.38×10^{-6}	231
2	0.54	1.3	2.06×10^{-6}	262
3	0.44	1.3	2.06×10^{-6}	214
			**Average**	**236 ± 20**

Table S10. Molar activity calculation of [^{18}F]FMZ using DMI as the reaction solvent.

**Measurement**	**Activity injected (MBq, d.c.)**	**Peak area (mAu*s)**	**FMZ injected (µmol)**	***A_m* (GBq/µmol)**
1	0.26	2.4	3.81×10^{-6}	68
2	0.41	1.3	2.06×10^{-6}	199
3	0.48	1.8	2.86×10^{-6}	168
			**Average**	**145 ± 56**

Radio and UV HPLC overlays and radiochemical yield calculations

UV/radio-HPLC trace overlay for 4-[^{18}F]fluorobenzonitrile ([^{18}F]2)

Prepared following the general procedure and analyzed by (radio)HPLC using conditions B.

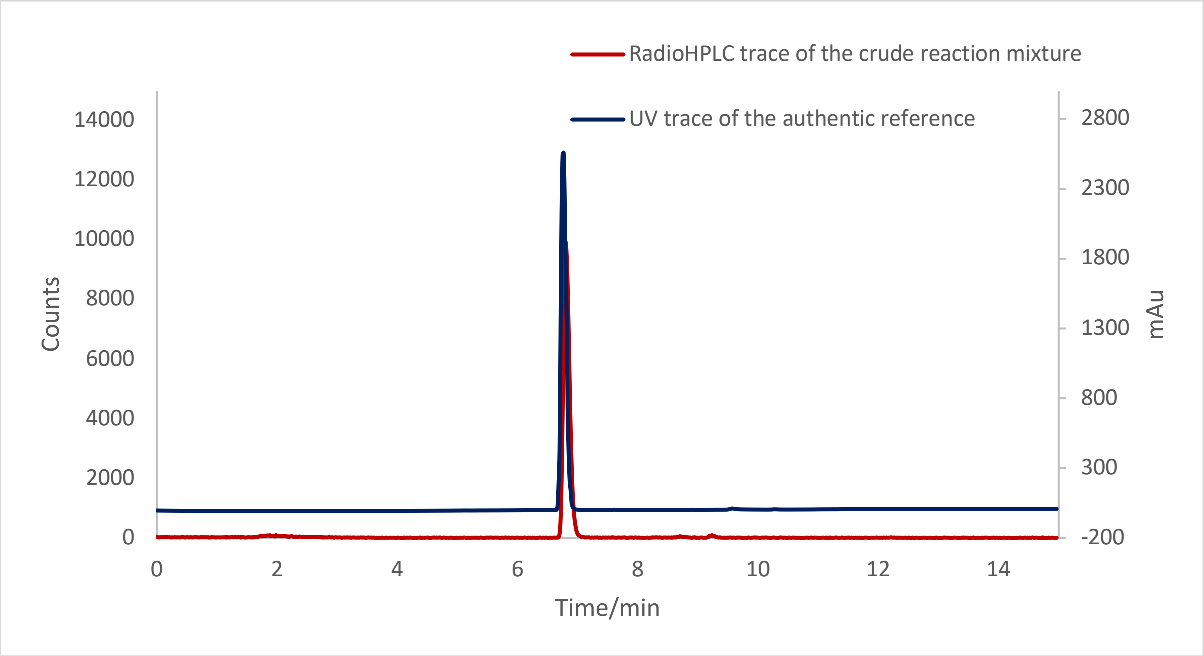

Run	RCY of [^{18}F]**2**	
1	97%	
2	93%	
	Average RCY	95% ± 2% (*n* = 2)

UV/radio-HPLC trace overlay for 4-[^{18}F]fluoro-1,1'-biphenyl ([^{18}F]3)

Prepared following the general procedure and analyzed by (radio)HPLC using conditions B.

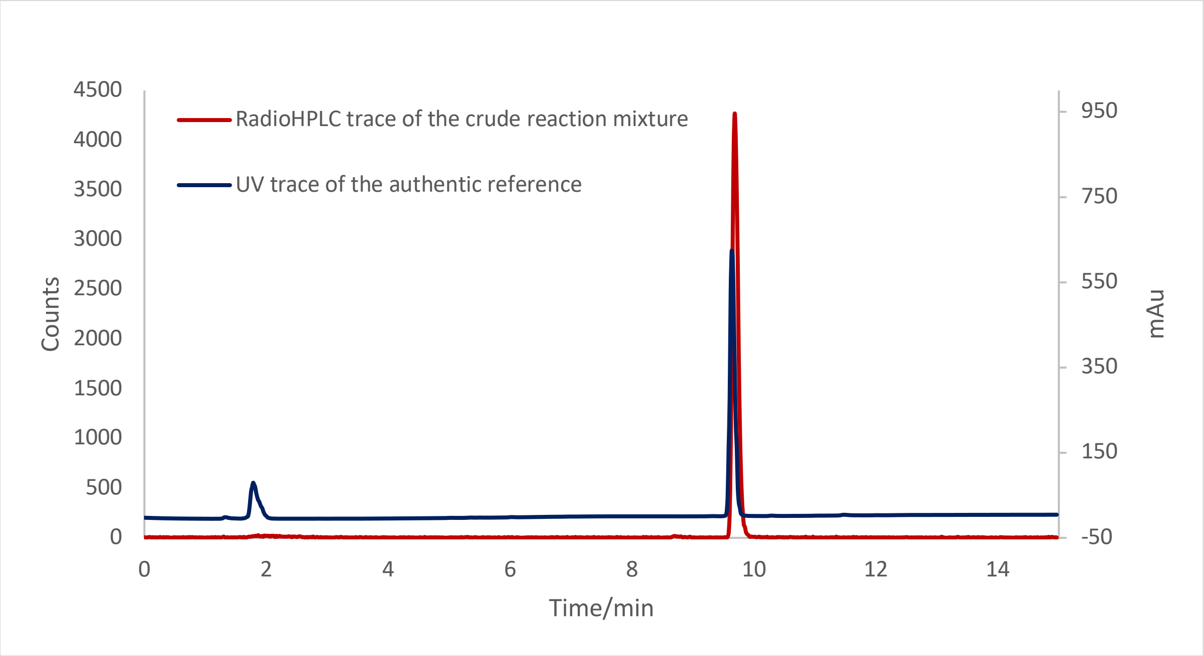

Run	RCY of [^{18}F]**3**	
1	96%	
2	96%	
	Average RCY	96% ± 0% (*n* = 2)

UV/radio-HPLC trace overlay for 1-[^{18}F]fluoronaphthalene ([^{18}F]4)

Prepared following the general procedure and analyzed by (radio)HPLC using conditions B.

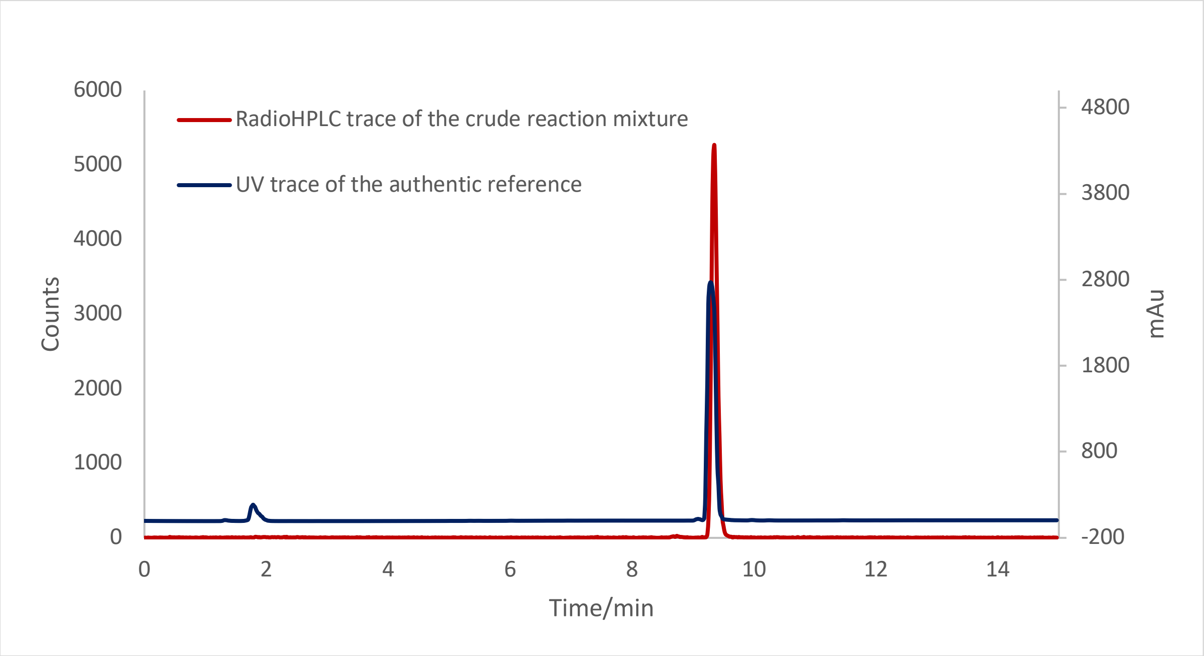

Run	RCY of [^{18}F]**4**	
1	98%	
2	98%	
	Average RCY	98% ± 0% (*n* = 2)

UV/radio-HPLC trace overlay for 2-[^{18}F]fluoro-1,3,5-trimethylbenzene ([^{18}F]5)

Prepared following the general procedure and analyzed by (radio)HPLC using conditions B.

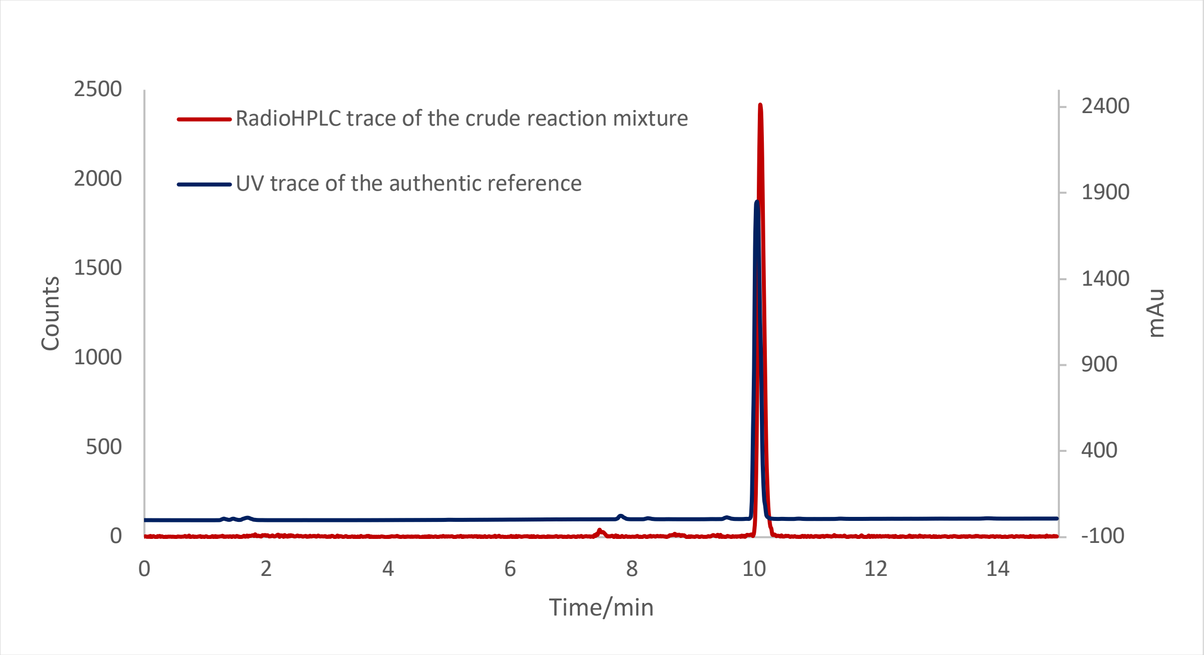

Run	RCY of [^{18}F]**5**	
1	94%	
2	95%	
	Average RCY	95% ± 1% (n = 2)

UV/radio-HPLC trace overlay for 1-[^{18}F]fluoro-4-methoxybenzene ([^{18}F]6)

Prepared following the general procedure and analyzed by (radio)HPLC using conditions B.

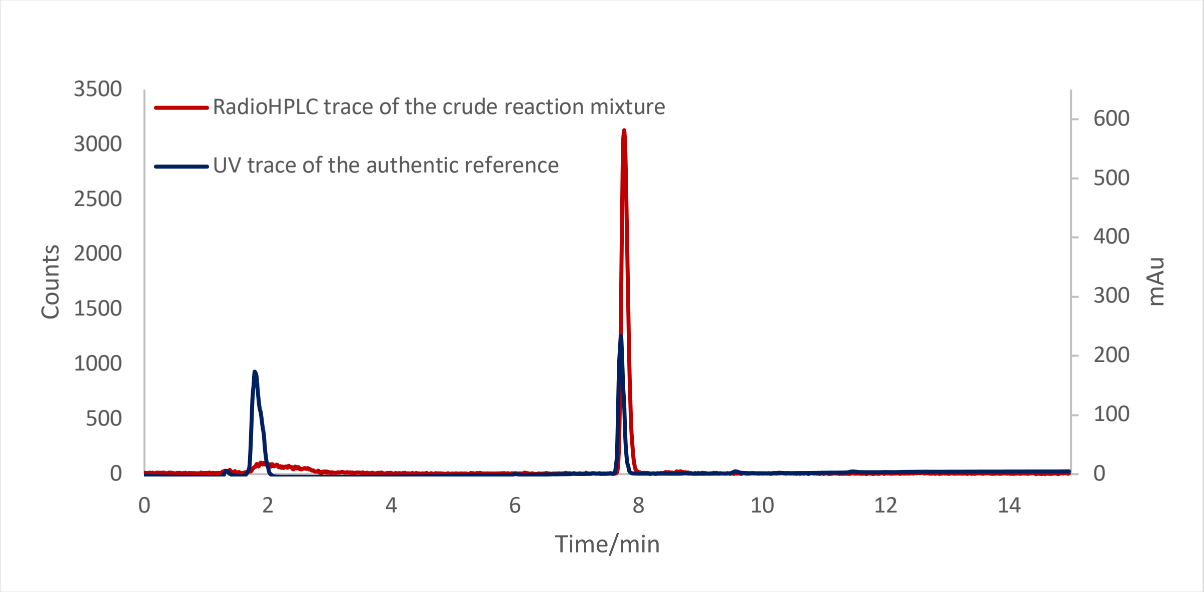

Run	RCY of [^{18}F]**6**	
1	82%	
2	92%	
	Average RCY	87% ± 5% (*n* = 2)

UV/radio-HPLC trace overlay for 4-[^{18}F]fluoro-1,2-dimethoxybenzene ([^{18}F]7)

Prepared following the general procedure and analyzed by (radio)HPLC using conditions B.

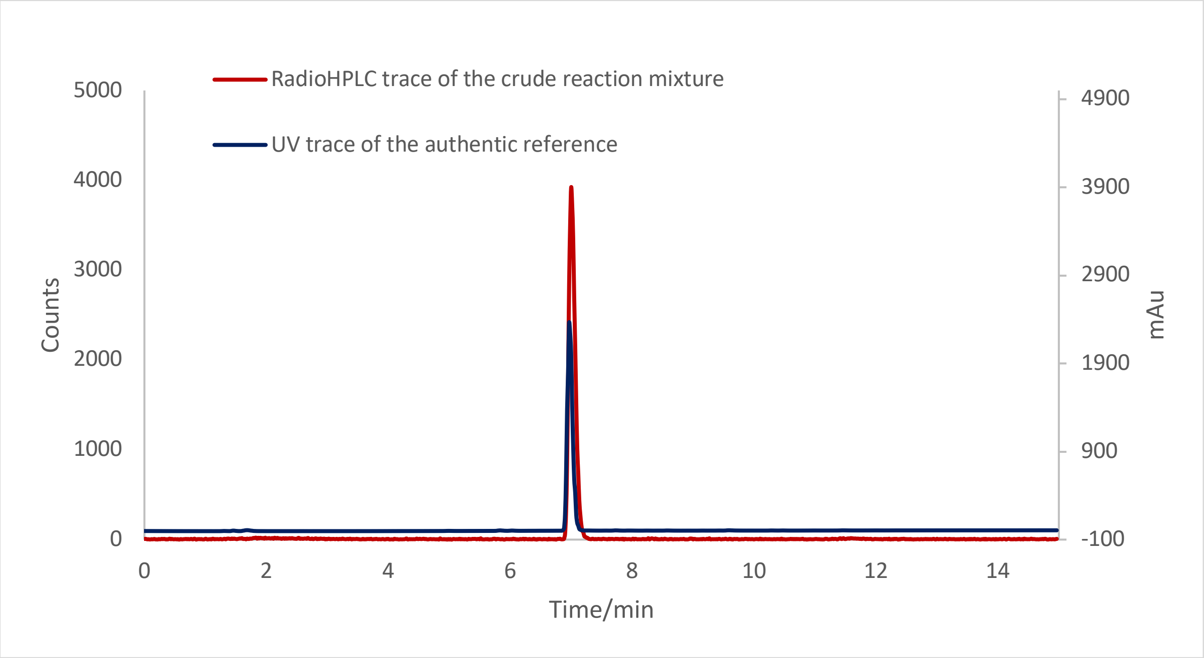

Run	RCY of [^{18}F]**7**	
1	97%	
2	95%	
	Average RCY	96% ± 1% (*n* = 2)

UV/radio-HPLC trace overlay for 1-[^{18}F]fluoro-4-(trifluoromethyl)benzene ([^{18}F]8)

Prepared following the general procedure and analyzed by (radio)HPLC using conditions B.

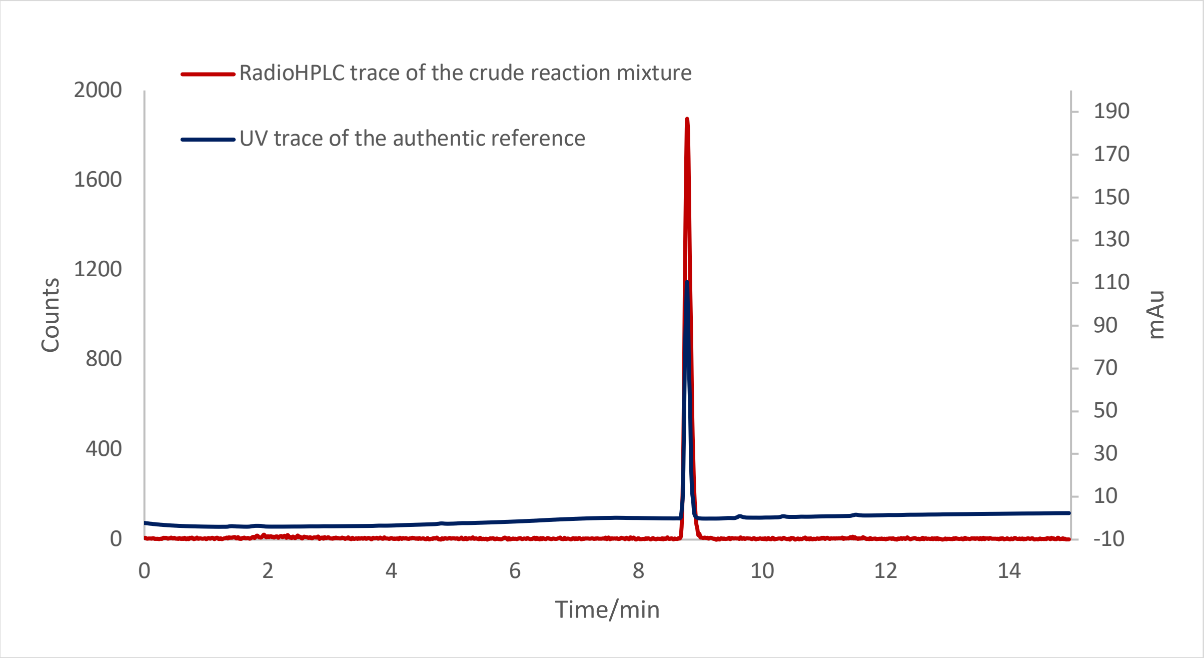

Run	RCY of [^{18}F]**8**	
1	93%	
2	93%	
	Average RCY	93% ± 0% (*n* = 2)

UV/radio-HPLC trace overlay for ethyl 4-[^{18}F]fluorobenzoate ([^{18}F]9)

Prepared following the general procedure and analyzed by (radio)HPLC using conditions B.

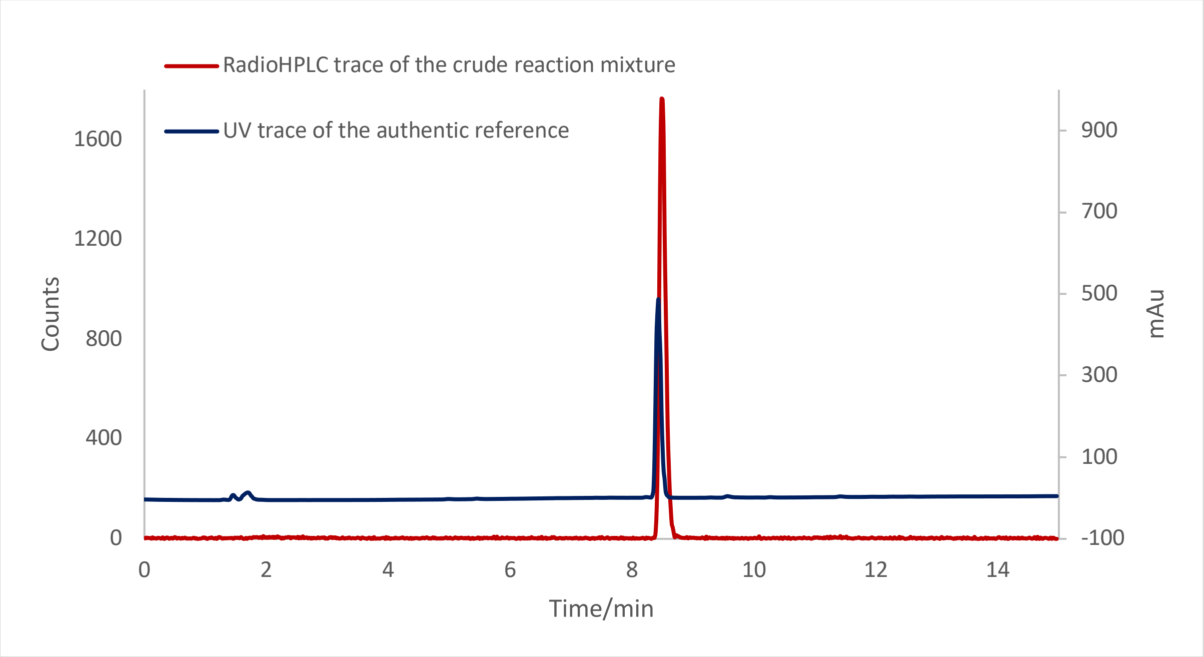

Run	RCY of [^{18}F]**9**	
1	98%	
2	98%	
	Average RCY	98% ± 0% (*n* = 2)

UV/radio-HPLC trace overlay for 1-[^{18}F]fluoro-3-nitrobenzene ([^{18}F]10)

Prepared following the general procedure and analyzed by (radio)HPLC using conditions B.

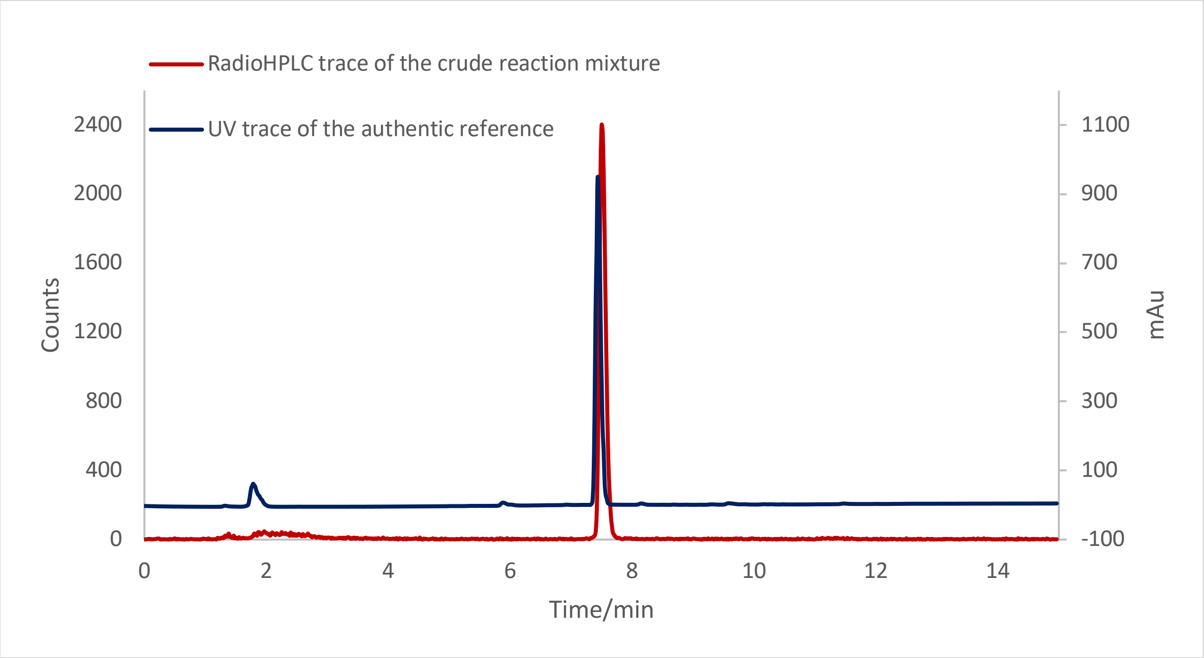

Run	RCY of [^{18}F]**10**	
1	88%	
2	94%	
	Average RCY	91% ± 3% (n = 2)

UV/radio-HPLC trace overlay for 1-[^{18}F]fluoro-4-(methylsulfonyl)benzene ([^{18}F]11)

Prepared following the general procedure and analyzed by (radio)HPLC using conditions B.

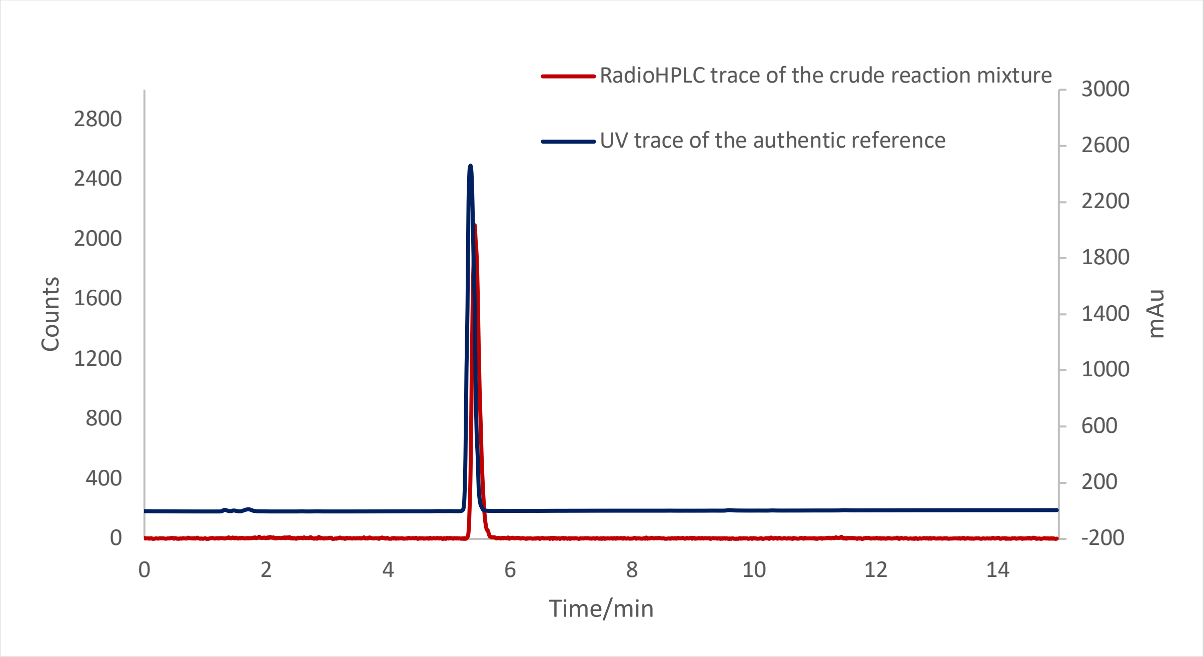

Run	RCY of [^{18}F]**11**	
1	98%	
2	97%	
	Average RCY	98% ± 1% (*n* = 2)

UV/radio-HPLC trace overlay for [^{18}F]4-((4-fluorophenyl)sulfonyl)morpholine ([^{18}F]12)

Prepared following the general procedure and analyzed by (radio)HPLC using conditions B.

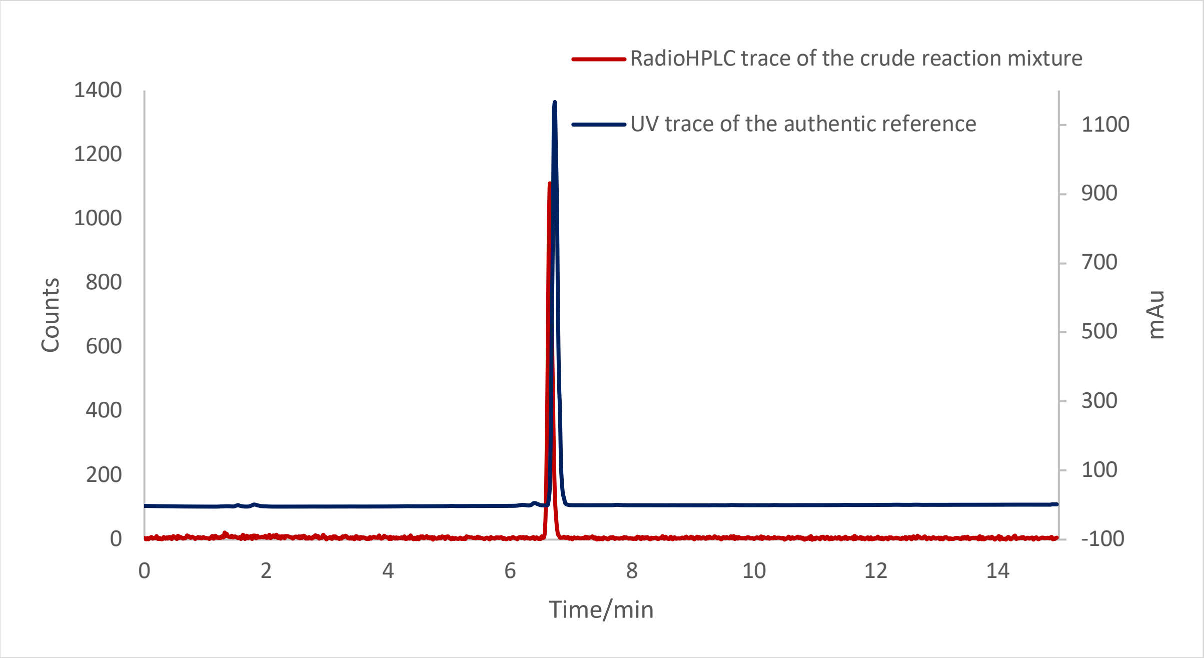

Run	RCY of [^{18}F]**12**	
1	93%	
2	94%	
	Average RCY	94% ± 1% (*n* = 2)

UV/radio-HPLC trace overlay for [^{18}F]2-(4-fluorophenyl)pyridine ([^{18}F]13)

Prepared following the general procedure and analyzed by (radio)HPLC using conditions B.

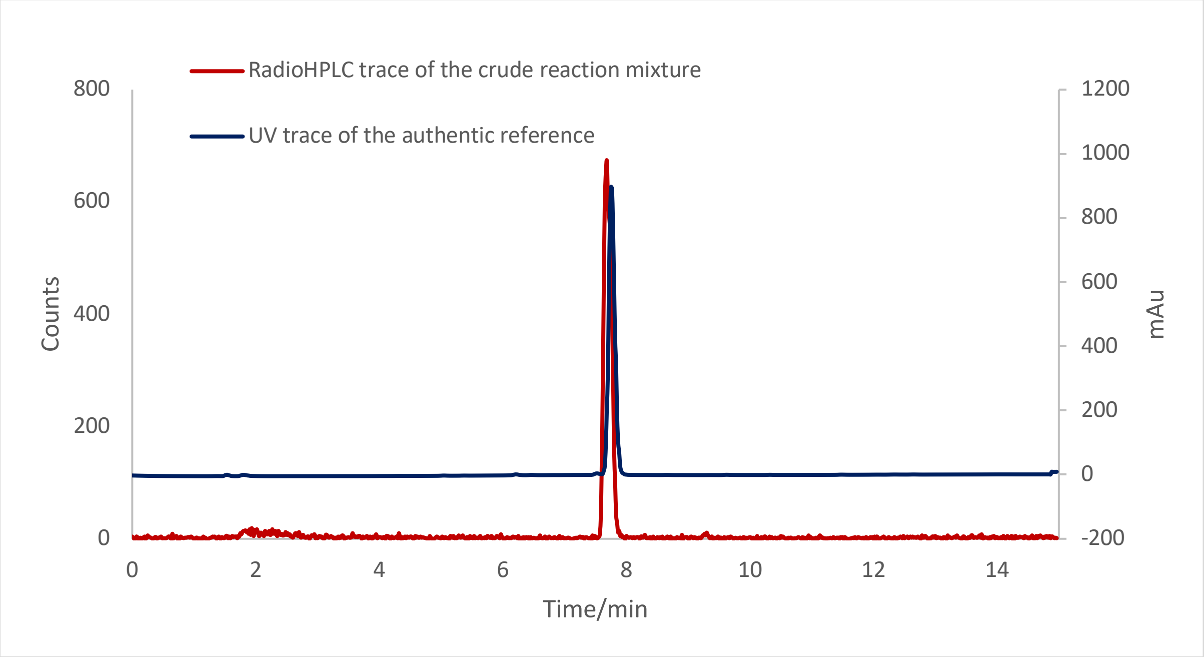

Run	RCY of [^{18}F]**13**	
1	88%	
2	89%	
	Average RCY	89% ± 1% (*n* = 2)

UV/radio-HPLC trace overlay for [^{18}F]4-fluoroquinoline ([^{18}F]14)

Prepared following the general procedure and analyzed by (radio)HPLC using conditions B.

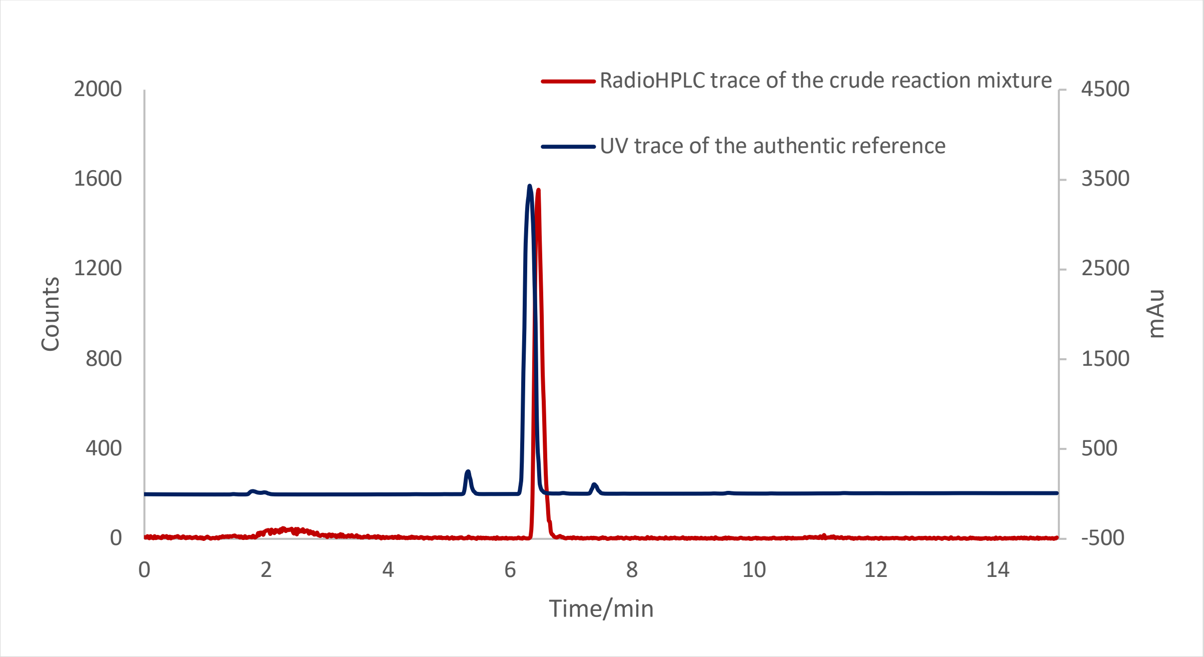

Run	RCY of [^{18}F]**14**	
1	81%	
2	84%	
	Average RCY	83% ± 2% (*n* = 2)

UV/radio-HPLC trace overlay for [^{18}F]6-fluoroquinoline ([^{18}F]15)

Prepared following the general procedure and analyzed by (radio)HPLC using conditions B.

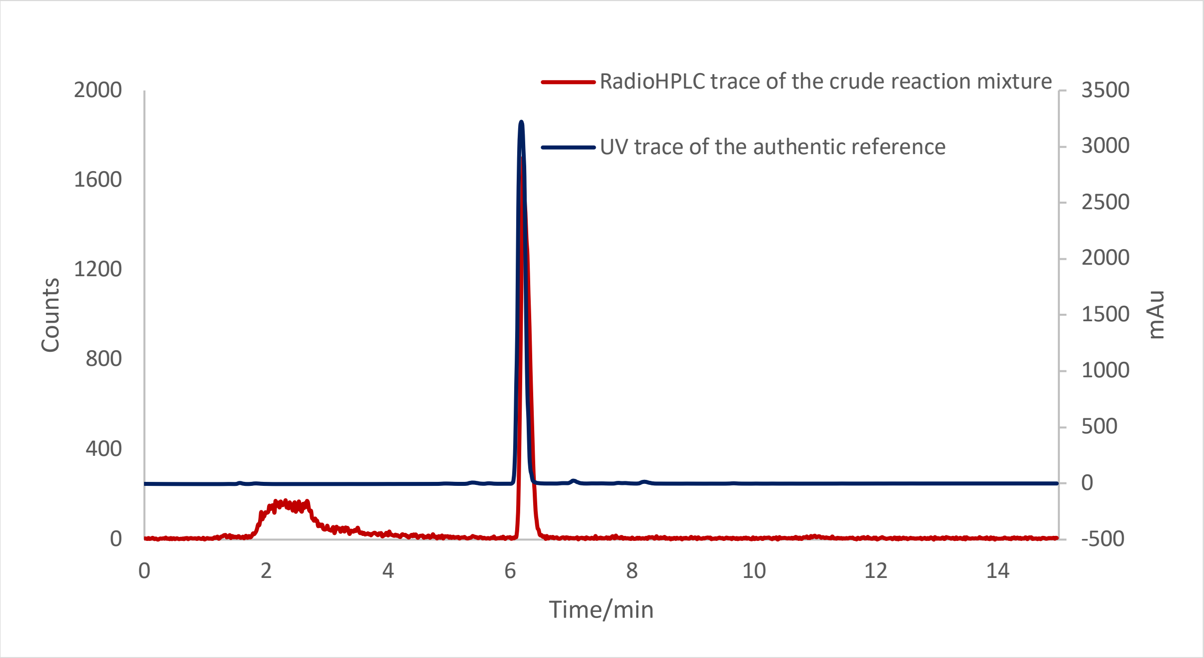

Run	RCY of [^{18}F]**15**	
1	63%	
2	66%	
	Average RCY	65% ± 2% (*n* = 2)

UV/radio-HPLC trace overlay for [^{18}F]5-fluoro-1-methyl-1*H*-indole ([^{18}F]16)

Prepared following the general procedure and analyzed by (radio)HPLC using conditions B.

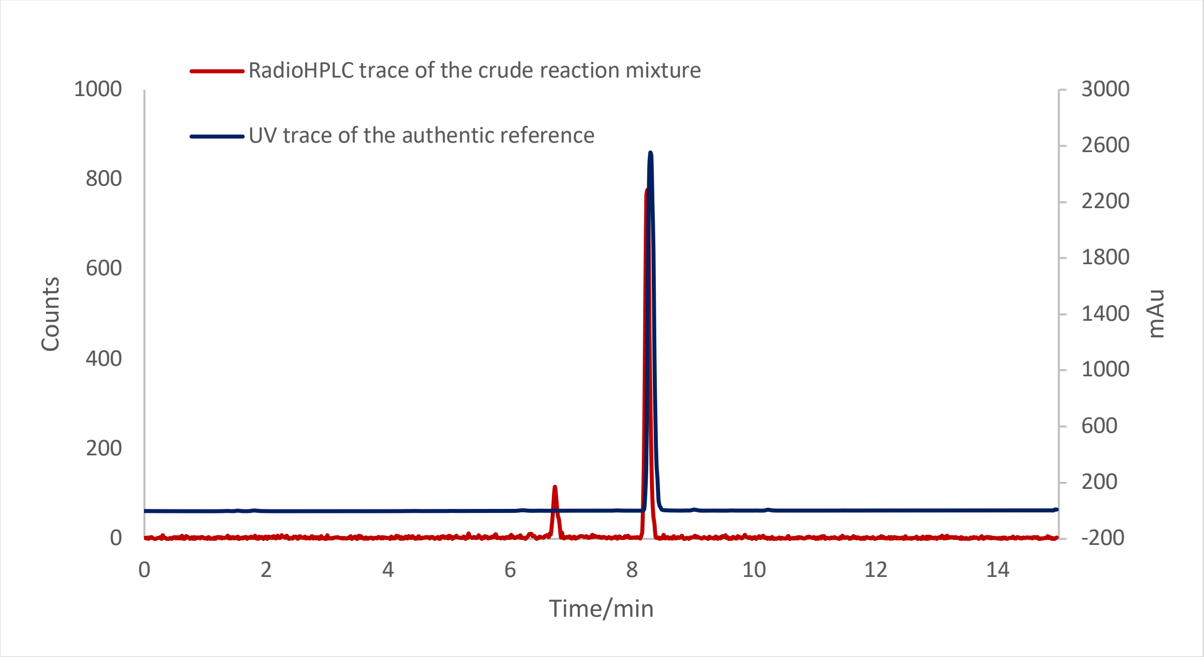

Run	RCY of [^{18}F]**16**	
1	83%	
2	83%	
	Average RCY	83% ± 0% (*n* = 2)

UV/radio-HPLC trace overlay for [^{18}F]5-fluorobenzo[*d*]oxazole ([^{18}F]17)

Prepared following the general procedure and analyzed by (radio)HPLC using conditions B.

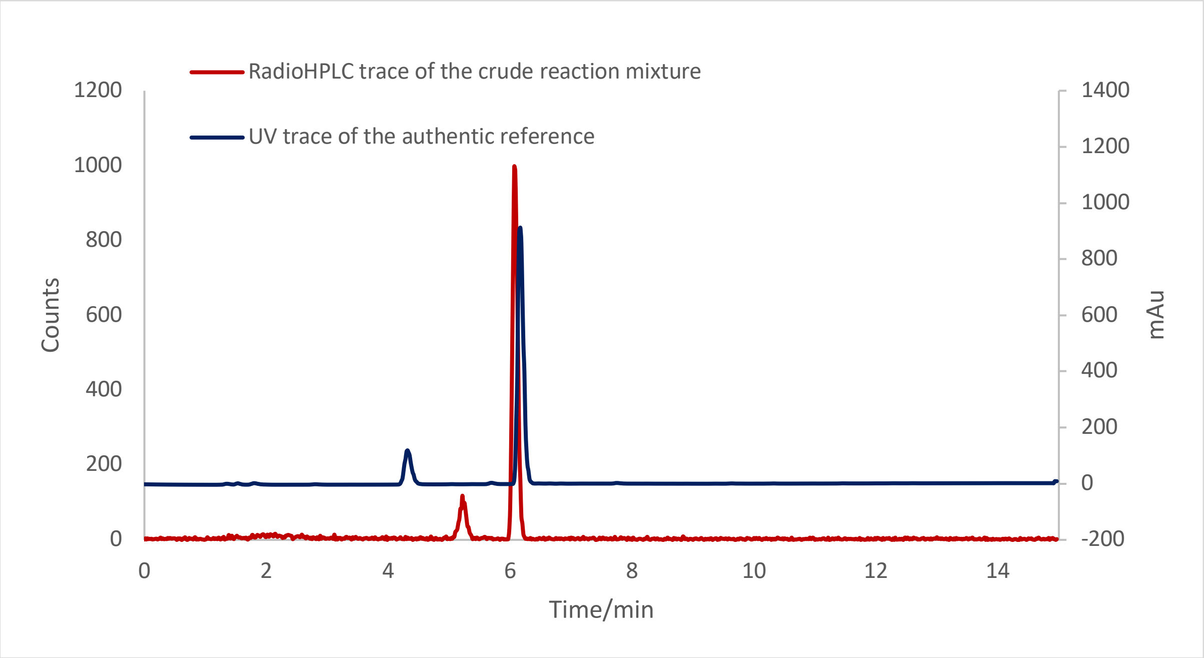

Run	RCY of [^{18}F]**17**	
1	79%	
2	81%	
	Average RCY	80% ± 1% (*n* = 2)

UV/radio-HPLC trace overlay for [^{18}F]3-fluoropyridine ([^{18}F]20)

Prepared following the general procedure and analyzed by (radio)HPLC using conditions C.

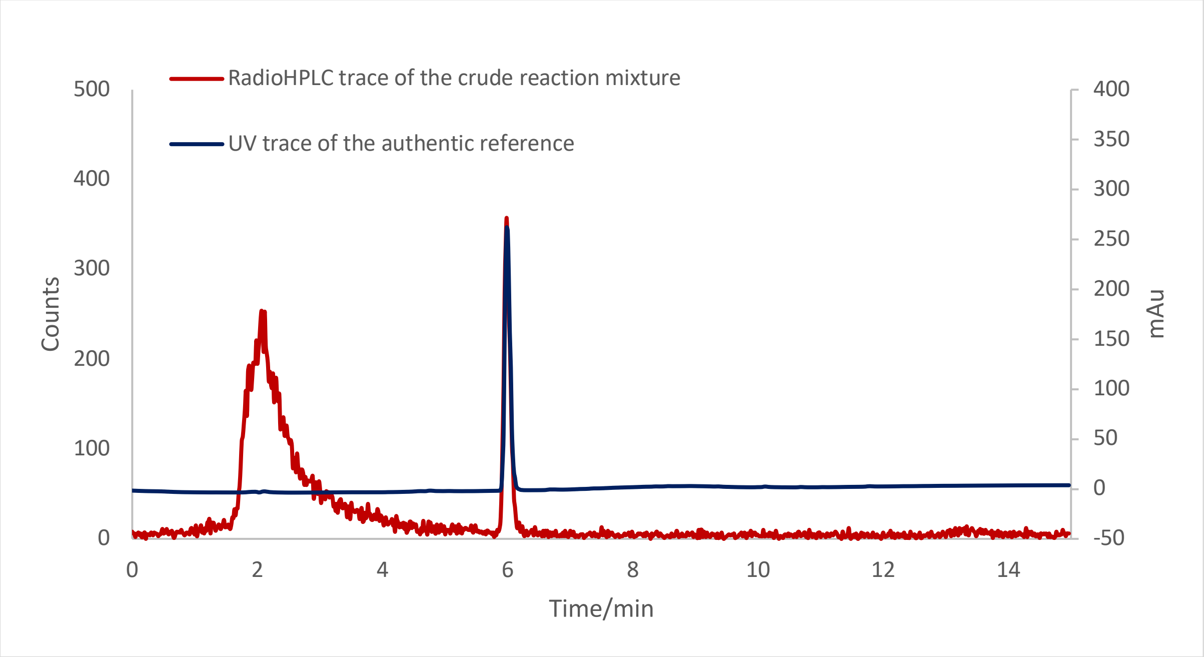

Run	RCY of [^{18}F]**20**	
1	11%	
2	18%	
	Average RCY	15% ± 4% (*n* = 2)

UV/radio-HPLC trace overlay for [^{18}F]5-fluoro-1*H*-indazole ([^{18}F]21)

Prepared following the general procedure and analyzed by (radio)HPLC using conditions B.

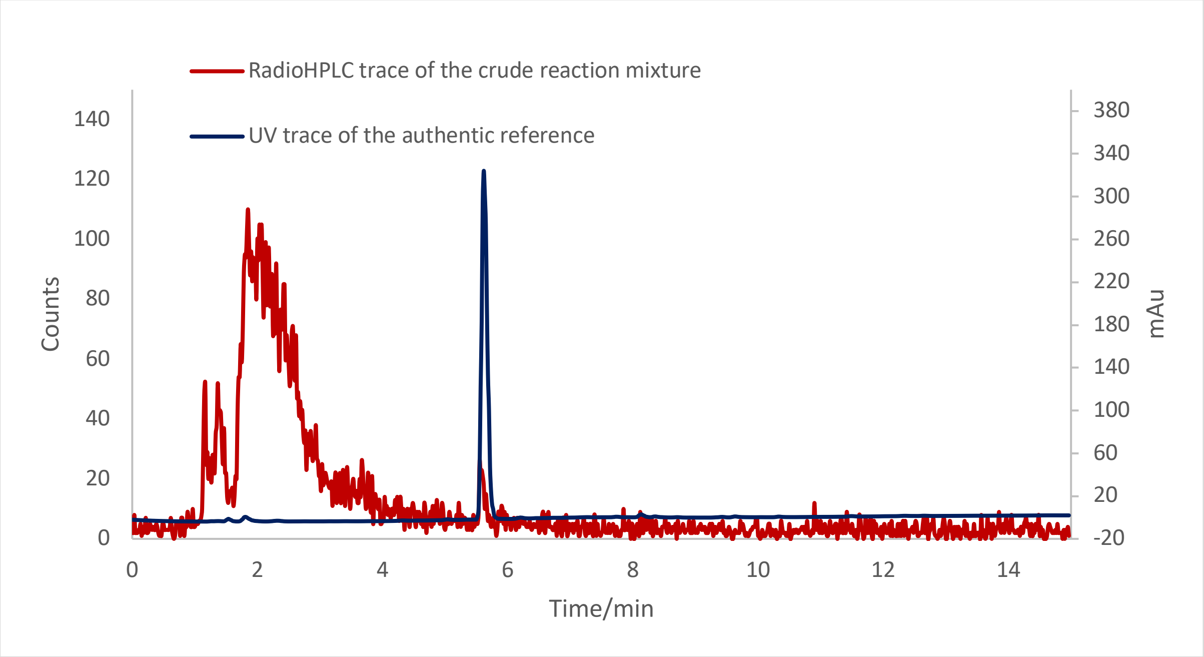

Run	RCY of [^{18}F]**21**	
1	2%	
2	3%	
	Average RCY	3% ± 1% (*n* = 2)

UV/radio-HPLC trace overlay for [^{18}F]3-fluoro-5-(pyridin-2-ylethynyl)benzonitrile ([^{18}F]24)

Prepared following the general procedure and analyzed by (radio)HPLC using conditions B.

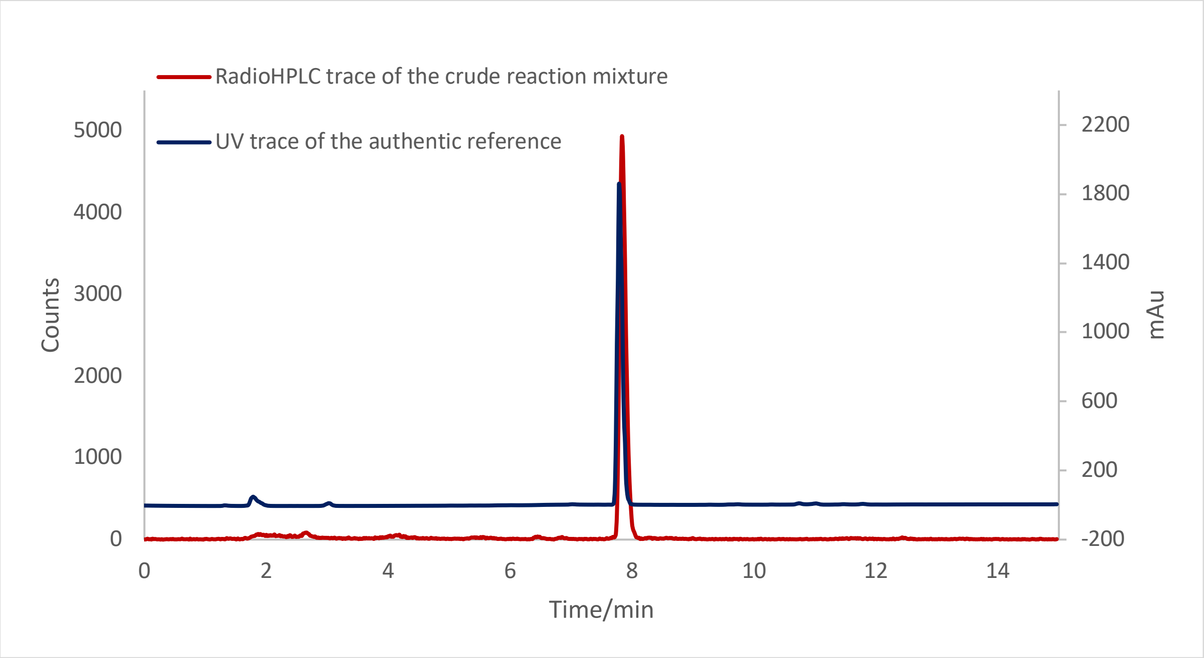

Run	RCY of [^{18}F]**24**	
1	84%	
2	81%	
	Average RCY	83% ± 2% (*n* = 2)

UV/radio-HPLC trace overlay for [^{18}F]FMZ ([^{18}F]26)

Prepared following the general procedure using 0.03 mmol of [$Cu(OTf)_2(py)_4$] instead of 0.02 mmol and analyzed by (radio)HPLC using conditions B.

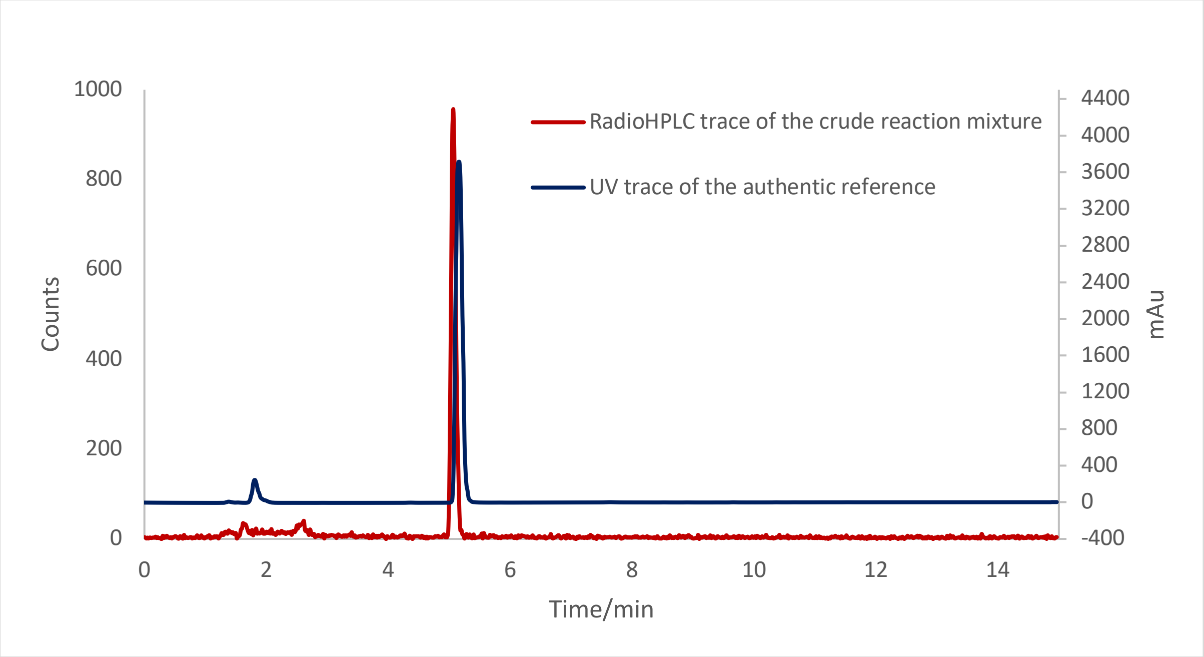

Run	RCY of [^{18}F]**26**	
1	91%	
2	81%	
	Average RCY	86% ± 5% (n = 2)

Computations

Computational methods

Geometry optimization, vibrational frequency and single point energy calculations were performed using *Gaussian 16, revision C.01*.[14] Geometry optimisation, frequency and single point energy calculations were performed at the M062X-D3/def2-TZVP level of theory.[15,16] Solvation in acetonitrile was modelled using the conductor-like polarizable continuum model (CPCM).[17,18] Ground state geometries were identified by the absence of imaginary frequency vibrational modes. 3D structures of molecules were generated by *CYLview*.[19] All bond lengths are in Angstroms (Å). Thermochemistry was evaluated at 298.15 K and a concentration of 0.1 mol/L using Paton's *GoodVibes* script.[20] Entropic contribution of low vibrational modes was corrected with a frequency cutoff value of 100 cm^{-1}.[21] Non-covalent interaction (NCI) was evaluated using *NCIPLOT-4.2*.[22] Graphical representation was generated by *VMD*.[23]
Conformational sampling of boronic esters was performed using a Global Optimizer Algorithm (GOAT) featured in *ORCA 6.0.0*,[24-26] based on the GFN2-XTB method.[27] Conformational sampling was run in acetonitrile using the extended conductor-like polarizable continuum model (CPCM-X).[28] A 25 kJ/mol energy window was applied during sampling.

*Conformers of model substrate BEpin **1a***

The 10 lowest-energy structures of **1a** (CONF1–10) were listed in order of increasing energy (Figure S22). Six conformers (CONF2, 4, 5, 6, 8, and 9) exhibited proximity between at least one methyl group and the boron atom, accounting for a combined Boltzmann population of 43%. Key C–H···B distances (2.62–2.99 Å) were summarized in Table S11. For visual representation, CONF2 and CONF5 were selected as examples.

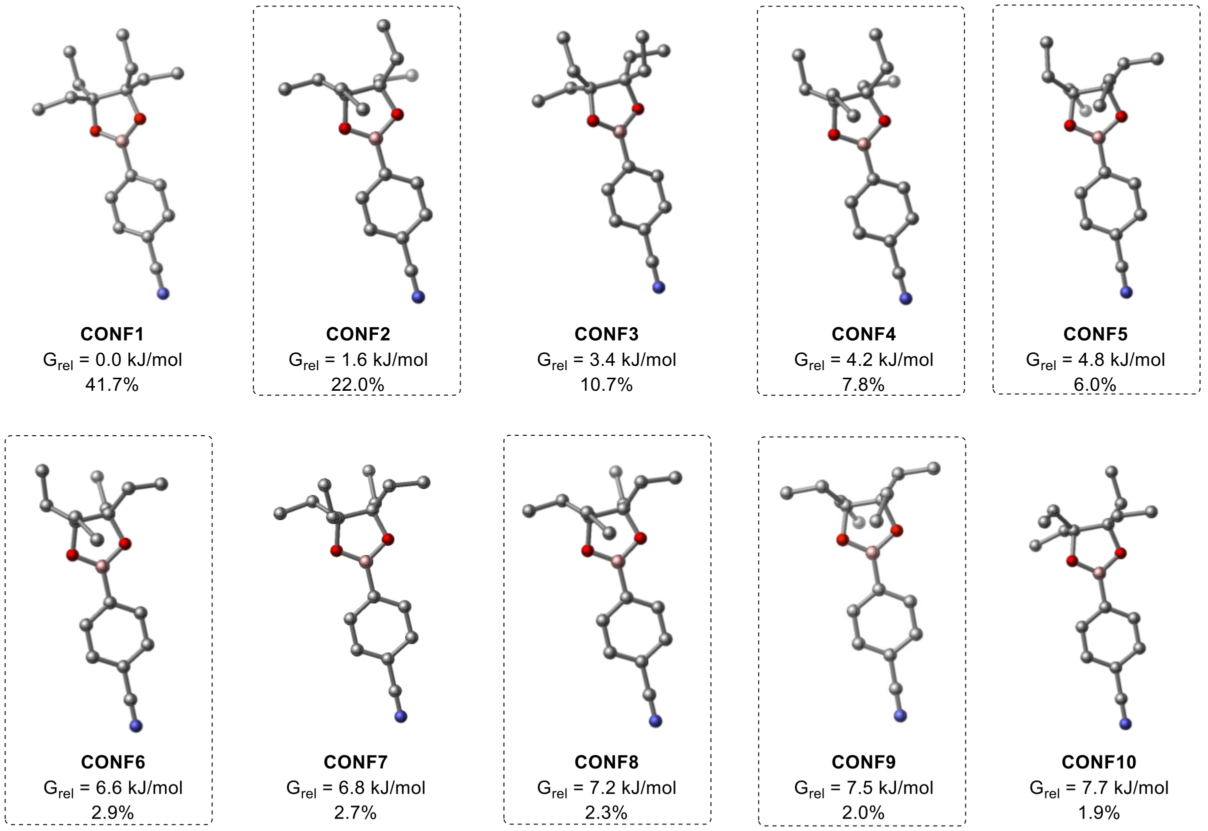

Figure S22. Conformers of model substrate **1a**. Boltzmann percentage populations are shown below each structure.

Table S11. Calculated distances from selected terminal C–H to boron atom.

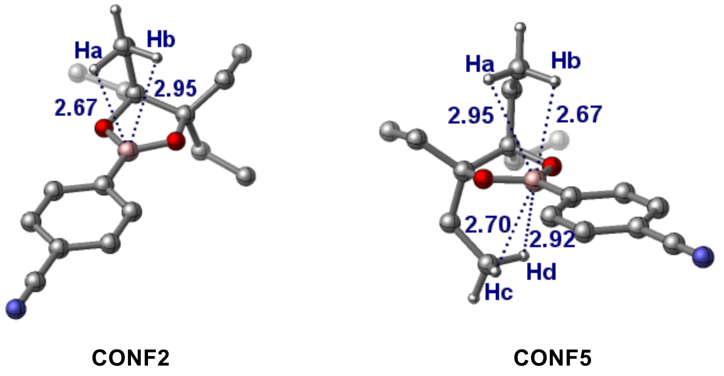

Structure	C–$H_{a/b}$···B / Å	C–$H_{c/d}$···B / Å
CONF2	2.67, 2.95	-
CONF4	2.80, 2.87	-
CONF5	2.67, 2.95	2.70, 2.92
CONF6	2.67, 2.94	-
CONF8	2.63, 2.98	-
CONF9	2.62, 2.99	2.62, 2.99

Non-covalent interaction (NCI) plot

A qualitative analysis of non-covalent interaction between the terminal C–H and boron is presented in Figure S23. Blue isosurface values, corresponding to large negative values of sign(λ_2)ρ, represent strong attractive interactions. Green isosurface values, corresponding to values of sign(λ_2)ρ close to 0, represent attractive, dispersion-dominated interactions. Red isosurface values, corresponding to large positive values of sign(λ_2)ρ, represent repulsive steric interactions. The NCI plot reveals the dispersion-dominated interactions between proximal protons and boron. These interactions are significant because accessing the anionic tetrahedral boronate intermediate is crucial to both boronic ester hydrolysis, and protodeborylation.[29] This additional dispersion interaction aligns with the experimentally observed stability of BEpin substrates.

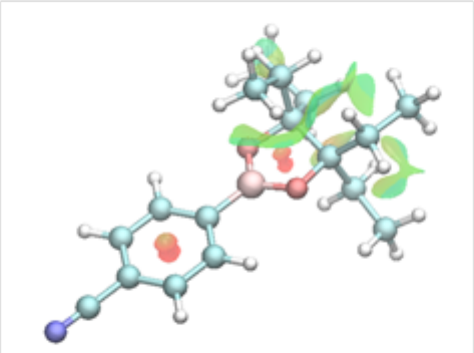

Figure S23. Non-covalent interaction plot for CONF2 of **1a**.

XYZ coordinates

1a CONF1
C -4.635801 -0.000073 -0.000016
C -3.948093 1.131043 -0.439275
C -2.563816 1.121051 -0.435294
C -1.853470 -0.000016 -0.000010
C -2.563772 -1.121112 0.435272
C -3.948049 -1.131161 0.439247
H -4.497579 1.999497 -0.775880
H -2.023869 1.996482 -0.773964
H -2.023790 -1.996520 0.773945
H -4.497501 -1.999638 0.775848
C -6.072062 -0.000105 -0.000020
N -7.220244 -0.000104 -0.000029
C 1.846224 -0.779577 0.139447
C 1.846192 0.779700 -0.139430
B -0.292053 0.000016 -0.000004
O 0.455447 1.027759 -0.487401
O 0.455484 -1.027694 0.487402
C 2.657642 1.271518 -1.337382
H 2.375527 0.693271 -2.216986
H 2.316601 2.289986 -1.530713
C 4.172301 1.275685 -1.177101
H 4.580101 0.269806 -1.084215
H 4.631759 1.736479 -2.051523
H 4.485080 1.846929 -0.301289
C 2.128163 -1.592486 -1.128729
H 3.126284 -1.354450 -1.499789
H 1.423986 -1.261467 -1.896668
C 1.997021 -3.099566 -0.942372
H 2.794965 -3.500066 -0.316190
H 1.042075 -3.353765 -0.481552
H 2.052000 -3.602376 -1.907672
C 2.657675 -1.271364 1.337411
H 2.375723 -0.692935 2.216949
H 2.316461 -2.289741 1.530917
C 4.172322 -1.275853 1.177024
H 4.484925 -1.847280 0.301268
H 4.580321 -0.270070 1.083973
H 4.631749 -1.736623 2.051474
C 2.128070 1.592622 1.128751
H 1.424071 1.261378 1.896757
H 3.126298 1.354836 1.499683
C 1.996491 3.099676 0.942483
H 1.041414 3.353634 0.481801
H 2.051458 3.602457 1.907799
H 2.794241 3.500424 0.316213

1a CONF2
C -4.494991 -0.064855 -0.036728
C -3.789865 -1.216524 0.312364
C -2.405863 -1.187738 0.301881
C -1.713126 -0.027575 -0.051946
C -2.440682 1.113835 -0.396961
C -3.824940 1.105566 -0.392682
H -4.326050 -2.114956 0.585930
H -1.852306 -2.078590 0.571404
H -1.914009 2.019767 -0.670200
H -4.387915 1.989538 -0.659437
C -5.931083 -0.084212 -0.028897
N -7.079149 -0.099620 -0.022673
C 1.991948 -0.742058 0.029603
C 1.954805 0.828086 -0.093152
B -0.152322 -0.003445 -0.055127
O 0.577273 1.065839 -0.487047
O 0.613327 -1.044895 0.380405
C 2.862937 1.384709 -1.190528
H 3.890379 1.072819 -0.987620
H 2.573370 0.917724 -2.130880
C 2.805810 2.899212 -1.369450
H 3.286425 3.176933 -2.307399
H 1.772378 3.246805 -1.405520
H 3.318869 3.431602 -0.569782
C 2.250662 -1.429159 -1.318944
H 3.240371 -1.155106 -1.685689
H 1.530173 -1.032056 -2.038978
C 2.128095 -2.947710 -1.266769
H 2.944310 -3.399135 -0.701602
H 1.187072 -3.248723 -0.805698

H 2.158868 -3.360426 -2.274968
C 2.889164 -1.307038 1.127472
H 2.664605 -0.817096 2.072541
H 2.610219 -2.354193 1.257258
C 4.384519 -1.211121 0.843110
H 4.710090 -0.178137 0.712855
H 4.947889 -1.628505 1.677367
H 4.662588 -1.765485 -0.053914
C 2.174531 1.560786 1.238938
H 3.175656 1.320773 1.605075
H 2.177422 2.628732 1.022109
C 1.131186 1.327852 2.330624
H 0.147883 1.671166 2.003418
H 1.037745 0.284935 2.627786
H 1.396180 1.906279 3.215823

1a CONF3
C 4.616807 -0.051428 -0.099853
C 3.922890 -1.193248 0.300292
C 2.539067 -1.166349 0.323729
C 1.835291 -0.017634 -0.045307
C 2.551778 1.113939 -0.441846
C 3.935745 1.107233 -0.472784
H 4.467334 -2.082904 0.585909
H 1.994404 -2.050024 0.631934
H 2.016870 2.010814 -0.728543
H 4.490087 1.983578 -0.779710
C 6.052689 -0.069033 -0.128270
N 7.200550 -0.083127 -0.150939
C -1.840411 0.822174 -0.018688
C -1.867188 -0.749839 0.098798
B 0.274740 0.002914 -0.013050
O -0.480182 -1.033247 0.448214
O -0.469963 1.062122 -0.440720
C -2.083162 -1.480279 -1.237058
H -1.474794 -0.986720 -2.000370
H -1.642041 -2.468821 -1.098350
C -3.514189 -1.653474 -1.740115
H -3.518716 -2.359503 -2.570972
H -4.171154 -2.055168 -0.967848
H -3.954268 -0.726471 -2.100699
C -2.743853 1.446061 -1.075283
H -2.607141 0.917749 -2.018499
H -2.372347 2.458092 -1.246780
C -4.219720 1.511775 -0.694515
H -4.608501 0.543317 -0.379047
H -4.387542 2.216568 0.120404
H -4.813081 1.843321 -1.546749
C -2.005989 1.503995 1.345241
H -3.003745 1.292556 1.733643
H -1.296806 1.045582 2.039687
C -1.773573 3.010108 1.311054
H -2.550338 3.528566 0.747998
H -1.777557 3.412394 2.323915
H -0.810800 3.244741 0.856797
C -2.745550 -1.305379 1.215418
H -2.436901 -0.848000 2.154624
H -3.780088 -0.999421 1.043573
C -2.663108 -2.821763 1.363782
H -1.626855 -3.139379 1.482326
H -3.216924 -3.139969 2.246791
H -3.080639 -3.345729 0.504264

1a CONF4
C 4.584084 0.055591 -0.057740
C 3.929600 -1.073398 -0.550126
C 2.545534 -1.097977 -0.560933
C 1.802988 -0.013407 -0.088295
C 2.480295 1.106076 0.400477
C 3.863733 1.150436 0.419593
H 4.504213 -1.913520 -0.915477
H 2.031027 -1.972330 -0.939692
H 1.914726 1.952549 0.769612
H 4.387803 2.017603 0.797513
C 6.019790 0.090870 -0.040841
N 7.167546 0.118966 -0.027218
C -1.919838 0.634555 0.104393
C -1.863534 -0.904160 -0.260244
B 0.242458 -0.055069 -0.098237
O -0.474347 -1.074963 -0.647005
O -0.534670 0.919693 0.448849
C -2.677696 -1.356288 -1.474917
H -2.419224 -0.732511 -2.330481
H -2.311013 -2.358537 -1.707821
C -4.191105 -1.412600 -1.308817
H -4.482346 -2.030218 -0.458126
H -4.629397 -0.424503 -1.174978
H -4.638497 -1.850892 -2.200913
C -2.244670 1.503240 -1.117090
H -3.236745 1.250090 -1.493603
H -1.535854 1.239562 -1.906379
C -2.164731 3.001696 -0.849689
H -2.968847 3.339077 -0.194855
H -1.214005 3.264268 -0.384977
H -2.248424 3.553867 -1.785530
C -2.745075 1.030261 1.328872
H -2.453981 0.408611 2.174239
H -2.434173 2.044653 1.584719
C -4.259566 0.999228 1.166431
H -4.636795 -0.011794 1.016956
H -4.730996 1.395292 2.065830
H -4.591010 1.609472 0.324728
C -2.124180 -1.840875 0.930746
H -3.085929 -1.585458 1.377814
H -2.235586 -2.850936 0.527536
C -1.038364 -1.870870 2.004057
H -0.835235 -0.887295 2.427477
H -1.342827 -2.527238 2.819222
H -0.103448 -2.259343 1.598879

1a CONF5
C 4.445762 -0.010712 0.025131
C 3.777342 -1.198232 0.322329
C 2.393053 -1.209836 0.321056
C 1.664321 -0.055021 0.027278
C 2.355458 1.122469 -0.267500
C 3.739383 1.154907 -0.270909
H 4.341613 -2.092415 0.549236
H 1.867487 -2.128637 0.549701
H 1.800446 2.023966 -0.495497
H 4.274497 2.066608 -0.498727
C 5.881833 0.012250 0.023845
N 7.029858 0.030616 0.022800
C -2.032190 0.669952 -0.010585
C -2.007057 -0.901894 0.058038
B 0.103765 -0.079650 0.027022
O -0.627199 -1.161517 0.425530
O -0.660044 0.978552 -0.372602
C -2.250200 -1.606821 -1.289052
H -2.323160 -2.675567 -1.069502
H -3.225771 -1.309314 -1.674498
C -1.183302 -1.412473 -2.363295
H -1.049462 -0.368472 -2.644577
H -0.217751 -1.796165 -2.029949
H -1.461675 -1.966286 -3.260016
C -2.961915 1.226090 -1.089653
H -3.980510 0.896891 -0.869981
H -2.690110 0.775538 -2.042301
C -2.929439 2.743634 -1.250084
H -3.429122 3.026259 -2.176508
H -3.436741 3.259087 -0.435949
H -1.901711 3.105971 -1.298403
C -2.297481 1.347428 1.344130
H -2.344567 2.420752 1.160794
H -3.290211 1.053208 1.691972
C -1.256596 1.125215 2.440190
H -0.285136 1.519324 2.135907
H -1.555634 1.664397 3.339267
H -1.122901 0.078461 2.706756
C -2.899468 -1.533120 1.126468
H -2.599022 -2.581199 1.195937
H -2.685274 -1.087427 2.095793
C -4.398110 -1.457902 0.849991
H -4.750518 -0.428278 0.778043
H -4.666963 -1.971078 -0.073054
H -4.946047 -1.933565 1.663152

1a CONF6
C 4.569864 0.072111 -0.166499
C 3.918495 -1.121176 -0.478203
C 2.534997 -1.159788 -0.450415
C 1.789989 -0.026131 -0.117360
C 2.464316 1.157296 0.192066
C 3.847194 1.216420 0.170526
H 4.495036 -1.998931 -0.736244
H 2.022931 -2.083648 -0.689016
H 1.897225 2.042407 0.451940
H 4.369081 2.132788 0.409990
C 6.005004 0.123332 -0.192603
N 7.152248 0.164363 -0.213589
C -1.856486 -0.963628 -0.034288
C -1.930824 0.609077 0.017585
B 0.230466 -0.083634 -0.084211
O -0.549413 0.952733 0.333210
O -0.487014 -1.181096 -0.462505
C -2.828131 1.181099 1.110084
H -3.854518 0.852920 0.930663
H -2.532339 0.756039 2.067659
C -2.774319 2.702300 1.213992
H -3.346821 3.037486 2.078564
H -1.745545 3.041581 1.337252
H -3.188075 3.191942 0.332654
C -2.022589 -1.650732 1.331622
H -2.060326 -2.725831 1.134447
H -2.998126 -1.383372 1.740824
C -0.931469 -1.393970 2.367323
H -0.840190 -0.342621 2.637151
H -1.151521 -1.954189 3.276153
H 0.040421 -1.732897 2.004289

C -2.751467 -1.651531 -1.061300
H -2.636283 -1.160240 -2.027099
H -2.348685 -2.659532 -1.184520
C -4.224087 -1.751631 -0.672589
H -4.806897 -2.129193 -1.512966
H -4.366881 -2.436077 0.163276
H -4.644431 -0.786774 -0.388011
C -2.180977 1.275971 -1.347074
H -1.764025 2.280292 -1.253845
H -1.565728 0.766682 -2.094195
C -3.618914 1.392764 -1.845160
H -4.045137 0.438324 -2.145608
H -4.276322 1.829153 -1.092342
H -3.643052 2.047537 -2.716719

1a CONF7
C 4.612169 -0.000003 -0.000000
C 3.924550 -1.195256 -0.209224
C 2.540278 -1.184695 -0.207079
C 1.829930 -0.000002 0.000006
C 2.540279 1.184690 0.207088
C 3.924551 1.195250 0.209227
H 4.474139 -2.112727 -0.369360
H 2.000420 -2.109599 -0.367219
H 2.000423 2.109594 0.367231
H 4.474142 2.112721 0.369361
C 6.048405 -0.000003 -0.000004
N 7.196583 -0.000004 -0.000009
C -1.860816 -0.787350 0.062312
C -1.860813 0.787353 -0.062311
B 0.268727 -0.000002 0.000009
O -0.481422 1.102214 0.280681
O -0.481425 -1.102213 -0.280676
C -2.764853 1.530293 0.913624
H -3.804005 1.280235 0.687531
H -2.563023 1.174684 1.923657
C -2.575027 3.044433 0.886086
H -2.865054 3.481406 -0.069183
H -3.184353 3.509303 1.660924
H -1.533157 3.303380 1.073372
C -2.039718 -1.294598 1.503094
H -1.460566 -0.650791 2.170206
H -1.554864 -2.271934 1.538375
C -3.470704 -1.441211 2.013016
H -4.019437 -2.194279 1.447399
H -3.455468 -1.761789 3.054988
H -4.036840 -0.513603 1.961966
C -2.764844 -1.530274 -0.913644
H -3.803996 -1.280178 -0.687592
H -2.562964 -1.174684 -1.923674
C -2.575065 -3.044418 -0.886073
H -2.865123 -3.481363 0.069201
H -3.184390 -3.509286 -1.660912
H -1.533199 -3.303401 -1.073333
C -2.039710 1.294580 -1.503100
H -1.554834 2.271905 -1.538399
H -1.460576 0.650746 -2.170201
C -3.470694 1.441212 -2.013019
H -4.036840 0.513609 -1.961980
H -4.019421 2.194280 -1.447394
H -3.455452 1.761803 -3.054987

1a CONF8
C -4.481001 -0.098771 -0.162975
C -3.764666 -1.283440 0.006693
C -2.381519 -1.235198 0.037953
C -1.700684 -0.023126 -0.097806
C -2.439467 1.150398 -0.265657
C -3.823072 1.123466 -0.299697
H -4.291449 -2.222075 0.111388
H -1.819267 -2.151355 0.168775
H -1.922212 2.096183 -0.368800
H -4.394726 2.032232 -0.429292
C -5.916311 -0.137962 -0.197196
N -7.063723 -0.169379 -0.224654
C 1.945608 0.874699 0.125315
C 2.014103 -0.688048 -0.053075
B -0.140924 0.022597 -0.053894
O 0.633717 -1.066348 0.216698
O 0.580011 1.161230 -0.273166
C 2.937286 -1.402529 0.926580
H 3.961542 -1.076196 0.724456
H 2.708576 -1.086323 1.941603
C 2.839698 -2.923811 0.861816
H 1.812008 -3.245621 1.031943
H 3.162095 -3.322497 -0.099588
H 3.466023 -3.371383 1.633240
C 2.100231 1.344522 1.580402
H 2.057334 2.433521 1.566703
H 3.104975 1.082525 1.920375
C 1.049354 0.866412 2.580235
H 1.011235 -0.216073 2.687289
H 1.260524 1.293953 3.560597
H 0.053917 1.208022 2.288836
C 2.889686 1.651479 -0.788939
H 3.916036 1.378219 -0.530497
H 2.718448 1.325403 -1.814131
C 2.732020 3.169141 -0.735569
H 1.681576 3.452297 -0.811483
H 3.137626 3.597181 0.179887
H 3.264218 3.623283 -1.571229
C 2.262171 -1.151048 -1.501169
H 1.813045 -2.143722 -1.569777
H 1.677084 -0.514804 -2.170538
C 3.707735 -1.241095 -1.983057
H 3.721995 -1.582776 -3.018469
H 4.231732 -0.288864 -1.944594
H 4.281485 -1.958047 -1.396035

1a CONF9
C 4.364766 -0.000019 -0.000005
C 3.677209 1.208556 -0.110249
C 2.292939 1.197863 -0.109625
C 1.582803 -0.000013 0.000007
C 2.292935 -1.197892 0.109632
C 3.677204 -1.208591 0.110244
H 4.226778 2.136131 -0.194426
H 1.752503 2.132518 -0.193747
H 1.752495 -2.132544 0.193758
H 4.226771 -2.136169 0.194415
C 5.800961 -0.000021 -0.000012
N 6.949135 -0.000020 -0.000015
C -2.095530 -0.777118 -0.106286
C -2.095519 0.777122 0.106324
B 0.021913 -0.000011 0.000015
O -0.725262 1.121408 -0.227520
O -0.725268 -1.121424 0.227545
C -2.316650 1.221443 1.562293
H -2.294900 2.311333 1.559807
H -3.327383 0.936594 1.863356
C -1.293509 0.757747 2.597350
H -1.552106 1.172204 3.571996
H -1.234043 -0.324245 2.698070
H -0.295706 1.124741 2.347937
C -3.065135 -1.509132 0.820210
H -4.071095 -1.134780 0.607213
H -2.844435 -1.234225 1.848808
C -3.043282 -3.031466 0.713041
H -3.512579 -3.390991 -0.201416
H -2.019123 -3.406489 0.742890
H -3.584961 -3.467869 1.551993
C -2.316662 -1.221416 -1.562252
H -2.294884 -2.311307 -1.559785
H -3.327408 -0.936588 -1.863299
C -1.293543 -0.757662 -2.597303
H -1.552144 -1.172084 -3.571963
H -1.234097 0.324336 -2.697975
H -0.295730 -1.124648 -2.347916
C -3.065128 1.509128 -0.820178
H -2.844542 1.234090 -1.848766
H -4.071105 1.134892 -0.607060
C -3.043116 3.031475 -0.713190
H -2.018919 3.406387 -0.743135
H -3.584790 3.467832 -1.552169
H -3.512334 3.391165 0.201241

1a CONF10
C -4.500732 -0.107684 -0.017459
C -3.828205 1.045392 0.387020
C -2.444172 1.060088 0.364446
C -1.719501 -0.057827 -0.055375
C -2.414685 -1.200858 -0.456742
C -3.798458 -1.235757 -0.441327
H -4.389068 1.911156 0.711585
H -1.915206 1.952412 0.675601
H -1.863312 -2.073607 -0.783724
H -4.336543 -2.121153 -0.751167
C -5.936675 -0.133780 0.002752
N -7.084542 -0.154595 0.018956
C 1.941457 0.810449 -0.030998
C 1.982103 -0.751136 -0.200878
B -0.159390 -0.026981 -0.076103
O 0.607118 -1.038154 -0.579573
O 0.572988 1.017682 0.407655
C 2.218427 -1.480270 1.129709
H 3.176486 -1.179896 1.555486
H 1.447486 -1.145261 1.826701
C 2.164246 -3.000100 1.020281
H 3.025385 -3.398259 0.482652
H 2.162023 -3.446626 2.014422
H 1.259996 -3.322159 0.502578
C 2.935372 1.389958 0.989256
H 3.264612 2.366672 0.633851
H 3.829515 0.764759 0.988853
C 2.416719 1.559041 2.413316
H 1.562981 2.234928 2.433375
H 2.107266 0.616784 2.861115
H 3.201868 1.984792 3.038749
C 2.074476 1.529487 -1.382014

H 3.116956 1.471473 -1.704980
H 1.480032 0.992046 -2.125012
C 1.612760 2.982571 -1.357805
H 0.555572 3.046715 -1.102844
H 2.169359 3.585004 -0.639603
H 1.751505 3.432023 -2.341026
C 2.883353 -1.274931 -1.313324
H 2.643208 -2.330960 -1.446340
H 2.617978 -0.787728 -2.250709
C 4.377305 -1.120597 -1.049222
H 4.670828 -0.073246 -0.967636
H 4.678736 -1.625566 -0.130041
H 4.947484 -1.557731 -1.868535

References

1. Preshlock, S.; Calderwood, S.; Verhoog, S.; Tredwell, M.; Huiban, M.; Hienzsch, A.; Gruber, S.; Wilson, T. C.; Taylor, N. J.; Cailly, T.; Schedler, M.; Collier, T. L.; Passchier, J.; Smits, R.; Mollitor, J.; Hoepping, A.; Mueller, M.; Genicot, C.; Mercier, J.; Gouverneur, V. Enhanced copper-mediated ^{18}F-fluorination of aryl boronic esters provides eight radiotracers for PET applications. *Chem. Commun.* **2016**, *52*, 8361–8364.
2. Wu, Z.; Vlaming, R.; Donohoe, M.; Pratt, D. A. Interrupted Homolytic Substitution Enables Organoboron Compounds to Inhibit Radical Chain Reactions Rather than Initiate Them. *J. Am. Chem. Soc.* **2024**, *146*, 1153–1166.
3. Xu, J.-X.; Zhao, F.; Yuan, Y.; Wu, X.-F. Ruthenium-Catalyzed Carbonylative Coupling of Anilines with Organoboranes by the Cleavage of Neutral Aryl C–N Bond. *Org. Lett.* **2020**, *22*, 2756–2760.
4. Ahn, S.-J.; Lee, C.-Y.; Kim, N.-K.; Cheon, C.-H. Metal-Free Protodeboronation of Electron-Rich Arene Boronic Acids and Its Application to *ortho*-Functionalization of Electron-Rich Arenes Using a Boronic Acid as a Blocking Group. *J. Org. Chem*. **2014**, *79*, 7277–7285.
5. Oka, N.; Yamada, T.; Sajiki, H.; Akai, S.; Ikawa; T. Aryl Boronic Esters Are Stable on Silica Gel and Reactive under Suzuki–Miyaura Coupling Conditions. *Org. Lett.* **2022**, *24*, 3510–3514.
6. Liu, X.; Wu, M.; Zeng, R.; Gang, G.; Li, Q.; Li, F.; Yuan, A.; Shi, C. Iridium(III) Complex Radical and Corresponding Ligand Radical Functionalized by a Tris(2,4,6-trichlorophenyl)methyl Unit: Synthesis, Structure, and Photophysical Properties. *Inorg. Chem*. **2022**, *61*, 20942–20948.
7. Nelson, C. B.; L'Heureux, S. J.; Wong, M. J.; Kuhn, S. L.; Ghiglietti, E.; Lipshutz, B. H. Environmentally friendly Miyaura Borylations allowing for green, 1-pot borylation/Suzuki–Miyaura couplings. *Green Chem*. **2024**, *26*, 10115–10122.
8. Taylor, N. J.; Emer, E.; Preshlock, S.; Schedler, M.; Tredwell, M.; Verhoog, S.; Mercier, J.; Genicot, C.; Gouverneur, V. Derisking the Cu-Mediated ^{18}F-Fluorination of Heterocyclic Positron Emission Tomography Radioligands. *J. Am. Chem. Soc*. **2017**, *139*, 8267–8276.
9. Feofanov, M.; Akhmetov, V.; Takayama, R.; Amsharov, K. Y. Facile Synthesis of Thienoacenes via Transition-Metal-Free Ladderization. *J. Org. Chem*. **2021**, *86*, 14759–14766.
10. Greulich, T. W.; Daniliuc, C. G.; Studer, A. N-Aminopyridinium Salts as Precursors for N-Centered Radicals – Direct Amidation of Arenes and Heteroarenes. *Org. Lett*. **2015**, *17*, 254–257.
11. Mills, L. R.; Patel, P.; Rousseaux, S. A. L. Decyanation–(hetero)arylation of malononitriles to access α-(hetero)arylnitriles. *Org. Biomol. Chem*. **2022**, *20*, 5933–5937.
12. Moon, B. S.; Kil, H. S.; Park, J. H.; Kim, J. S.; Park, J.; Chi, D. Y.; Lee, B. C.; Kim, S. E. Facile aromatic radiofluorination of [^{18}F]flumazenil from diaryliodonium salts with evaluation of their stability and selectivity. *Org. Biomol. Chem*. **2011**, *9*, 8346–8355.

13. Gendron, T.; Destro, G.; Straathof, N. J. W.; Sap, J. B. I.; Guibbal, F.; Vriamont, C.; Caygill, C.; Atack, J. R.; Watkins, A. J.; Marshall, C.; Hueting, R.; Warnier, C.; Gouverneur, V.; Tredwell, M. Multi-patient dose synthesis of [^{18}F]Flumazenil via a copper-mediated ^{18}F-fluorination. *EJNMMI Radiopharm. Chem*. **2022**, *7*, 5.
14. Gaussian 16, Revision C.01; Frisch, M. J.; Trucks, G. W.; Schlegel, H. B.; Scuseria, G. E.; Robb, M. A.; Cheeseman, J. R.; Scalmani, G.; Barone, V.; Petersson, G. A.; Nakatsuji, H.; Li, X.; Caricato, M.; Marenich, A. V.; Bloino, J.; Janesko, B. G.; Gomperts, R.; Mennucci, B.; Hratchian, H. P.; Ortiz, J. V.; Izmaylov, A. F.; Sonnenberg, J. L.; Williams-Young, D.; Ding, F.; Lipparini, F.; Egidi, F.; Goings, J.; Peng, B.; Petrone, A.; Henderson, T.; Ranasinghe, D.; Zakrzewski, V. G.; Gao, J.; Rega, N.; Zheng, G.; Liang, W.; Hada, M.; Ehara, M.; Toyota, K.; Fukuda, R.; Hasegawa, J.; Ishida, M.; Nakajima, T.; Honda, Y.; Kitao, O.; Nakai, H.; Vreven, T.; Throssell, K.; Montgomery, J. A., Jr.; Peralta, J. E.; Ogliaro, F.; Bearpark, M. J.; Heyd, J. J.; Brothers, E. N.; Kudin, K. N.; Staroverov, V. N.; Keith, T. A.; Kobayashi, R.; Normand, J.; Raghavachari, K.; Rendell, A. P.; Burant, J. C.; Iyengar, S. S.; Tomasi, J.; Cossi, M.; Millam, J. M.; Klene, M.; Adamo, C.; Cammi, R.; Ochterski, J. W.; Martin, R. L.; Morokuma, K.; Farkas, O.; Foresman, J. B.; Fox, D. J. Gaussian, Inc., Wallingford CT, **2016**.
15. Zhao, Y.; Truhlar, D. G. The M06 suite of density functionals for main group thermochemistry, thermochemical kinetics, noncovalent interactions, excited states, and transition elements: two new functionals and systematic testing of four M06-class functionals and 12 other functionals. *Theor. Chem. Acc.* **2008**, *120*, 215–241.
16. Weigend, F.; Ahlrichs, R. Balanced basis sets of split valence, triple zeta valence and quadruple zeta valence quality for H to Rn: Design and assessment of accuracy. *Phys. Chem. Chem. Phys.* **2005**, *7*, 3297–3305.
17. Cossi, M.; Rega, N.; Scalmani, G.; Barone, V. Energies, structures, and electronic properties of molecules in solution with the C-PCM solvation model. *J. Comput. Chem.* **2003**, *24*, 669–681.
18. Barone, V.; Cossi, M. Quantum Calculation of Molecular Energies and Energy Gradients in Solution by a Conductor Solvent Model. *J. Phys. Chem. A* **1998**, *102*, 1995–2001.
19. CYLview20; Legault, C. Y., Université de Sherbrooke, **2020**.
20. Luchini, G.; Alegre-Requena, J.; Funes-Ardoiz, I.; Paton, R. GoodVibes: automated thermochemistry for heterogeneous computational chemistry data. *F1000Research* **2020**, *9*, 291.
21. Grimme, S. Supramolecular Binding Thermodynamics by Dispersion-Corrected Density Functional Theory. *Chem. Eur. J.* **2012**, *18*, 9955–9964.
22. Boto, R. A.; Peccati, F.; Laplaza, R.; Quan, C.; Carbone, A.; Piquemal, J.-P.; Maday, Y.; Contreras-García, J. NCIPLOT4: Fast, Robust, and Quantitative Analysis of Noncovalent Interactions. *J. Chem. Theory Comput.* **2020**, *16*, 4150–4158.
23. Humphrey, W.; Dalke, A.; Schulten, K. VMD: Visual molecular dynamics. *J. Mol. Graph.* **1996**, *14*, 33–38.
24. de Souza, B. GOAT: A Global Optimization Algorithm for Molecules and Atomic Clusters. *Angew. Chem., Int. Ed.* **2025**, *64*, e202500393.
25. Neese, F.; Wennmohs, F.; Becker, U.; Riplinger, C. The ORCA quantum chemistry program package. *J. Chem. Phys.* **2020**, *152*, 224108.
26. Neese, F. Software update: The ORCA program system—Version 5.0. *WIREs Comput. Mol. Sci.* **2022**, *12*, e1606.
27. Bannwarth, C.; Ehlert, S.; Grimme, S. GFN2-xTB—An Accurate and Broadly Parametrized Self-Consistent Tight-Binding Quantum Chemical Method with Multipole Electrostatics

and Density-Dependent Dispersion Contributions. *J. Chem. Theory Comput.* **2019**, *15*, 1652–1671.
28. Stahn, M.; Ehlert, S.; Grimme, S. Extended Conductor-like Polarizable Continuum Solvation Model (CPCM-X) for Semiempirical Methods. *J. Phys. Chem. A* **2023**, *127*, 7036–7043.
29. Hayes, H. L. D.; Wei, R.; Assante, M.; Geogheghan, K. J.; Jin, N.; Tomasi, S.; Noonan, G.; Leach, A. G.; Lloyd-Jones, G. C. Protodeboronation of (Hetero)Arylboronic Esters: Direct versus Prehydrolytic Pathways and Self-/Auto-Catalysis. *J. Am. Chem. Soc.* **2021**, *143*, 14814–14826.

NMR spectra for novel compounds

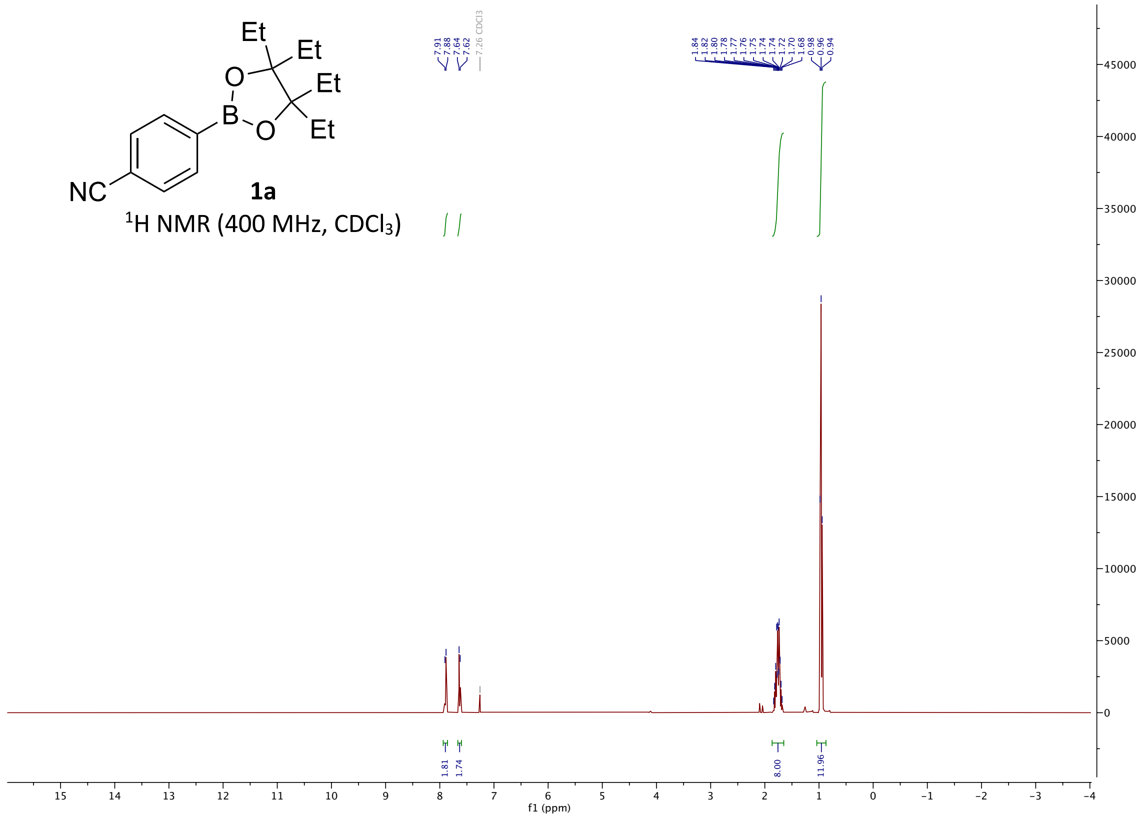

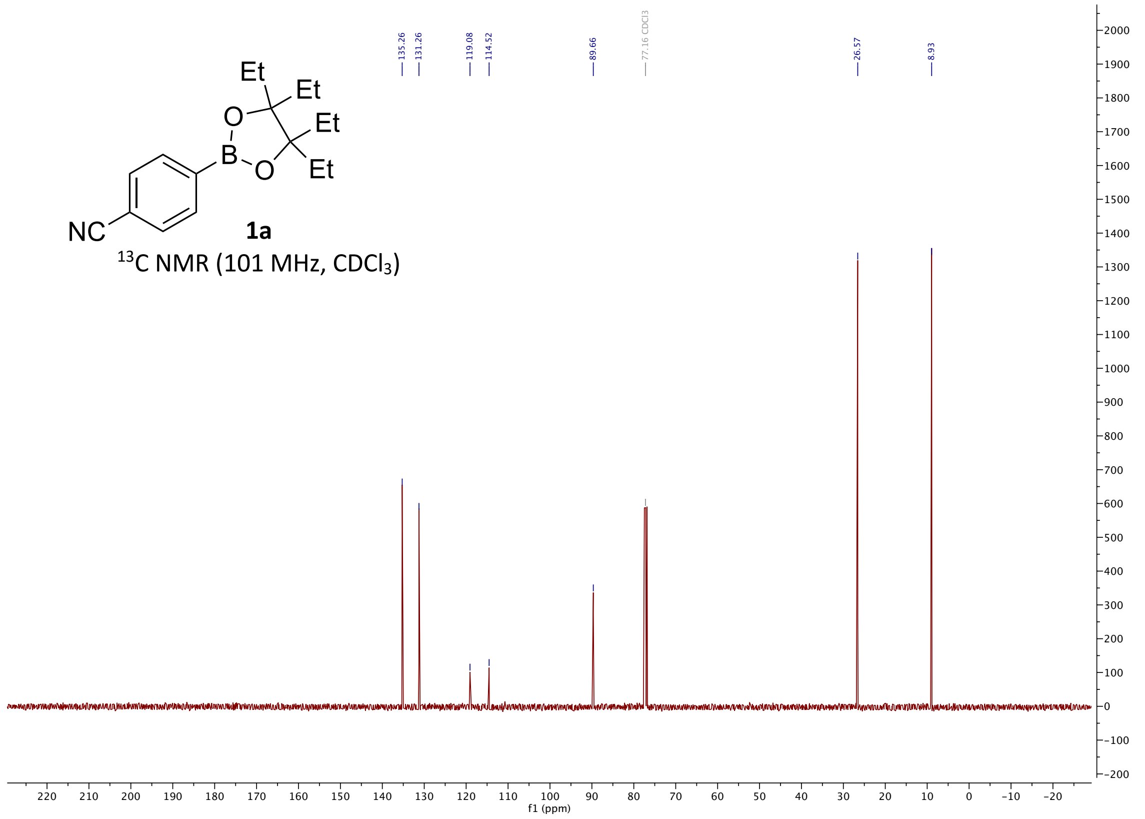

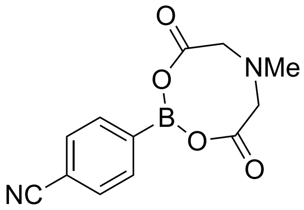

1e

^1H NMR (400 MHz, DMSO-d_6)

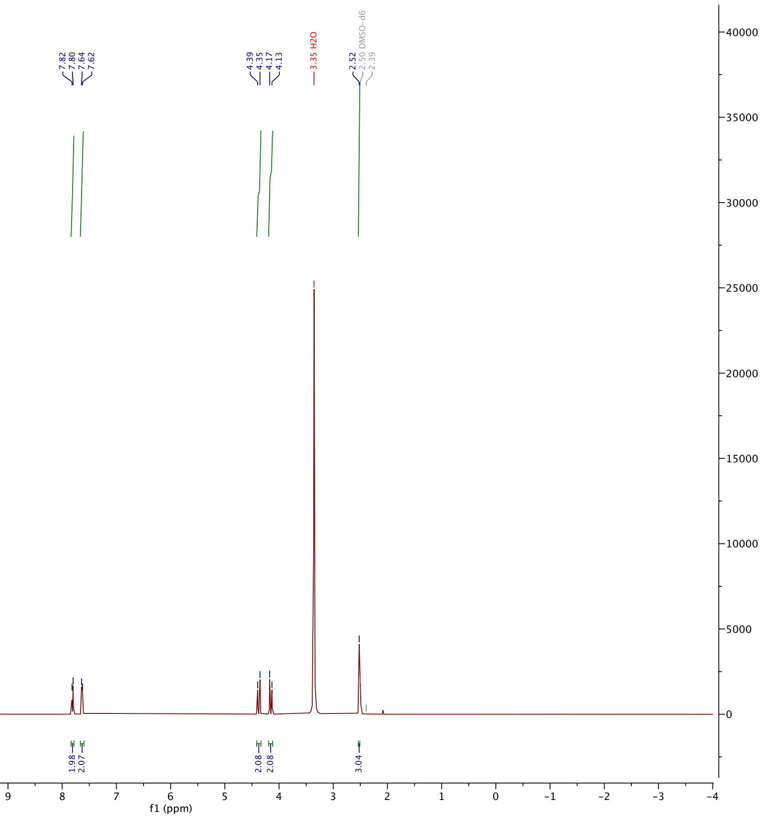

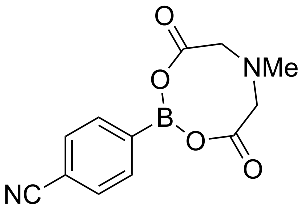

1e

^{13}C NMR (101 MHz, DMSO-d_6)

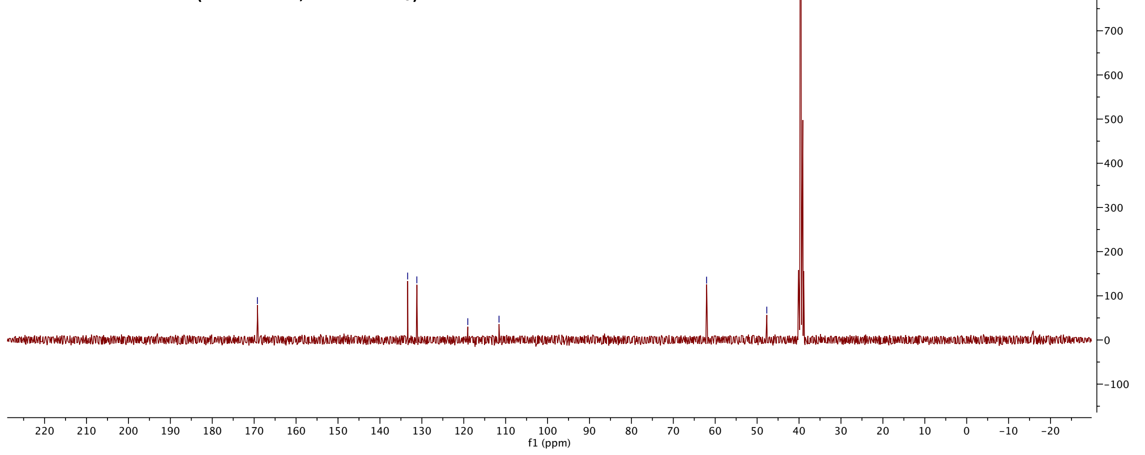

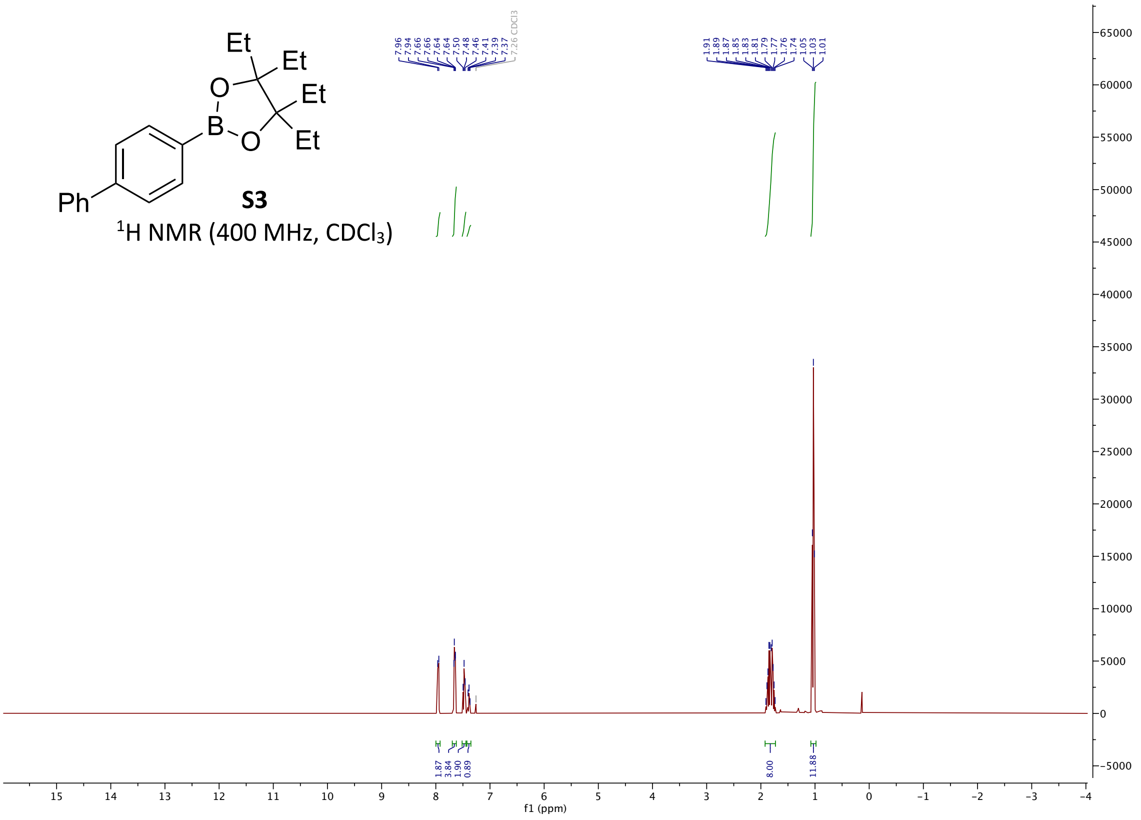

Et
Et
O
Et
B
O
Et
Ph
S3
^{1}H NMR (400 MHz, CDCl$_3$)
f1 (ppm)

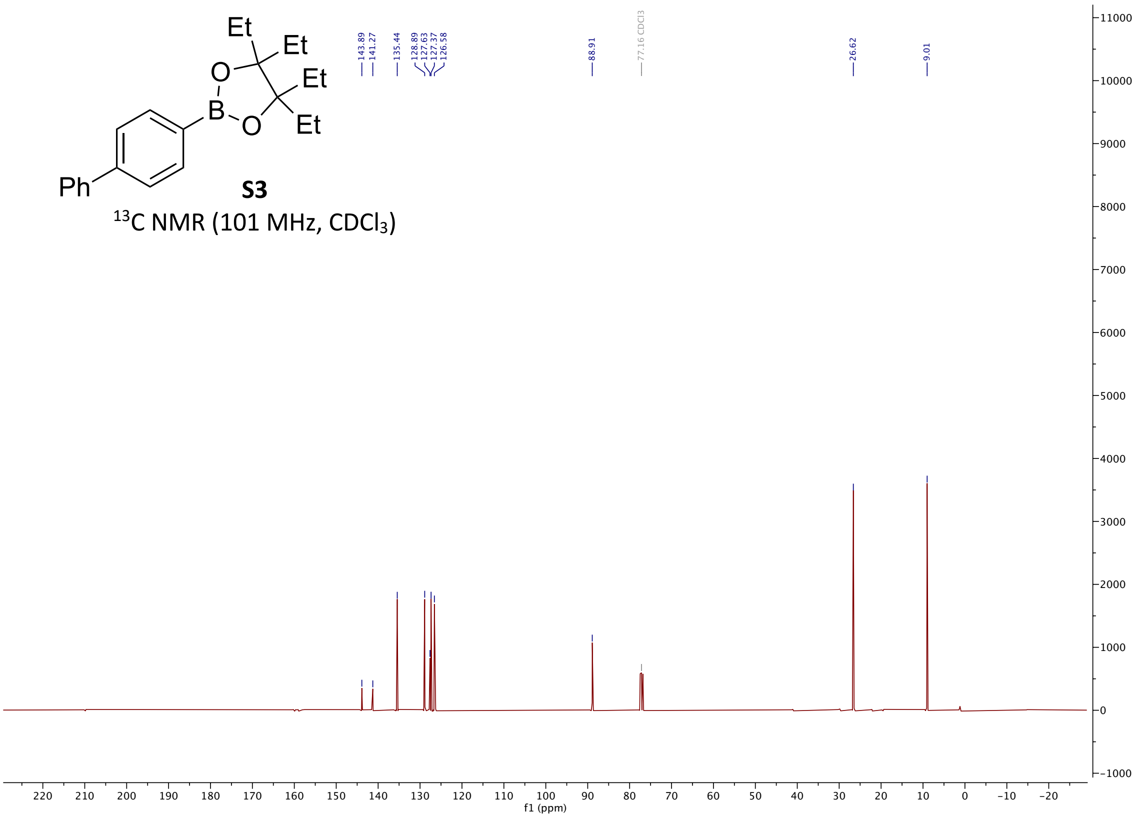

Et
Et
O
Et
B
O
Et
Ph
S3
^{13}C NMR (101 MHz, CDCl$_3$)
143.89
141.27
135.44
128.89
127.63
127.37
126.58
88.91
77.16 CDCl3
26.62
9.01
f1 (ppm)

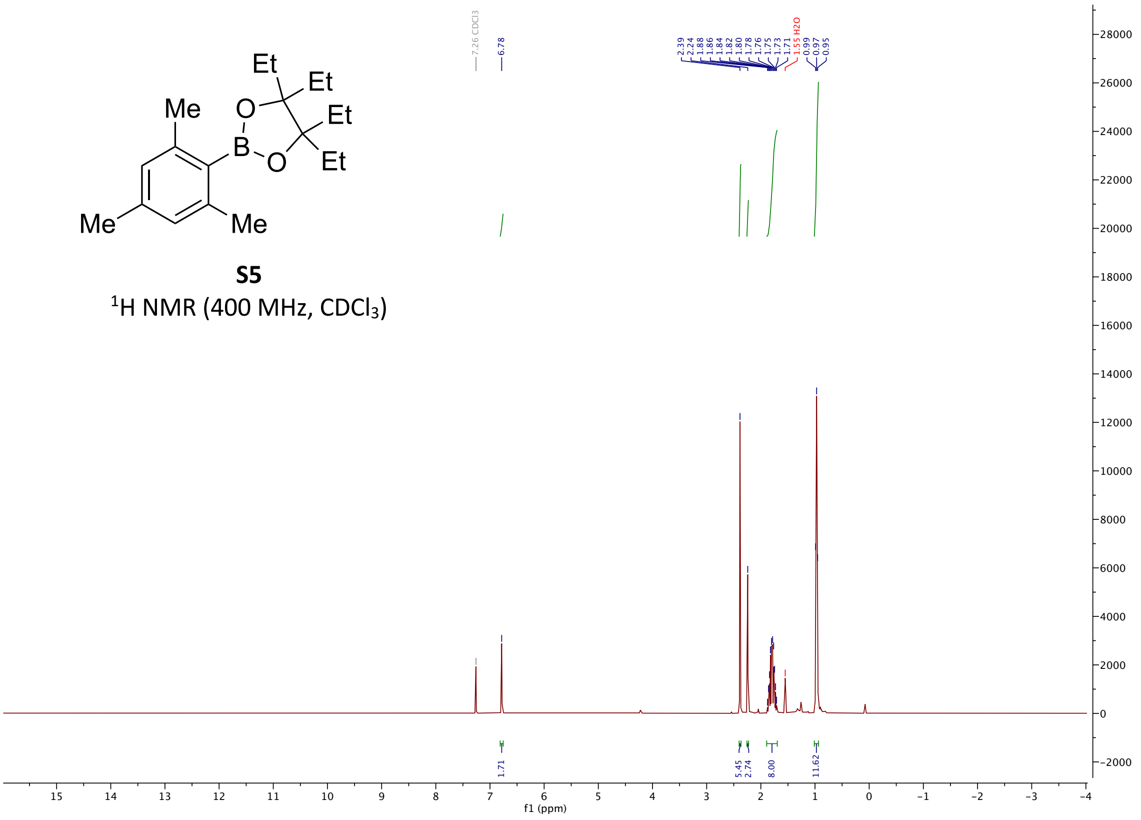
S5
^{1}H NMR (400 MHz, CDCl$_3$)
7.26 CDCl3
6.78
2.39
2.24
1.88
1.86
1.84
1.82
1.80
1.78
1.76
1.75
1.73
1.71
1.55 H2O
0.99
0.97
0.95
1.71
5.45
2.74
8.00
11.62
f1 (ppm)

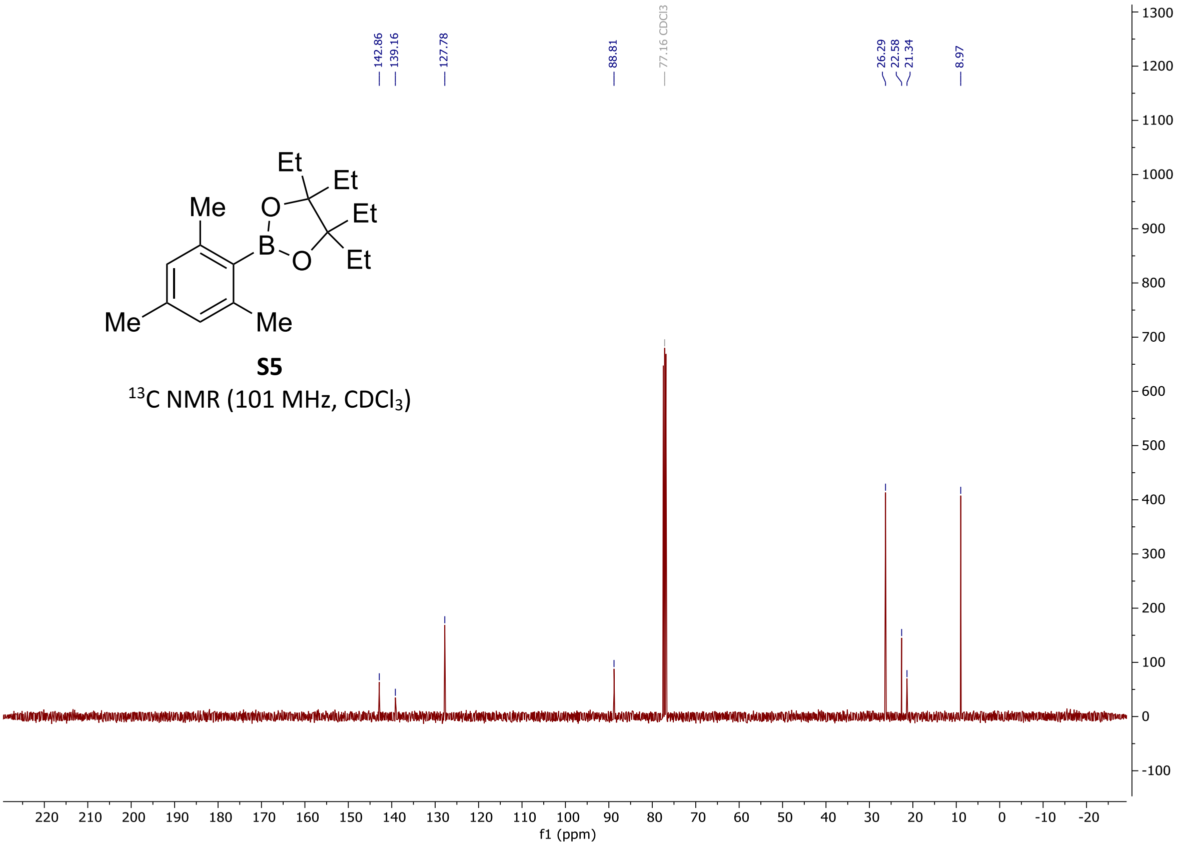
S5
^{13}C NMR (101 MHz, CDCl$_3$)
142.86
139.16
127.78
88.81
77.16 CDCl3
26.29
22.58
21.34
8.97
f1 (ppm)

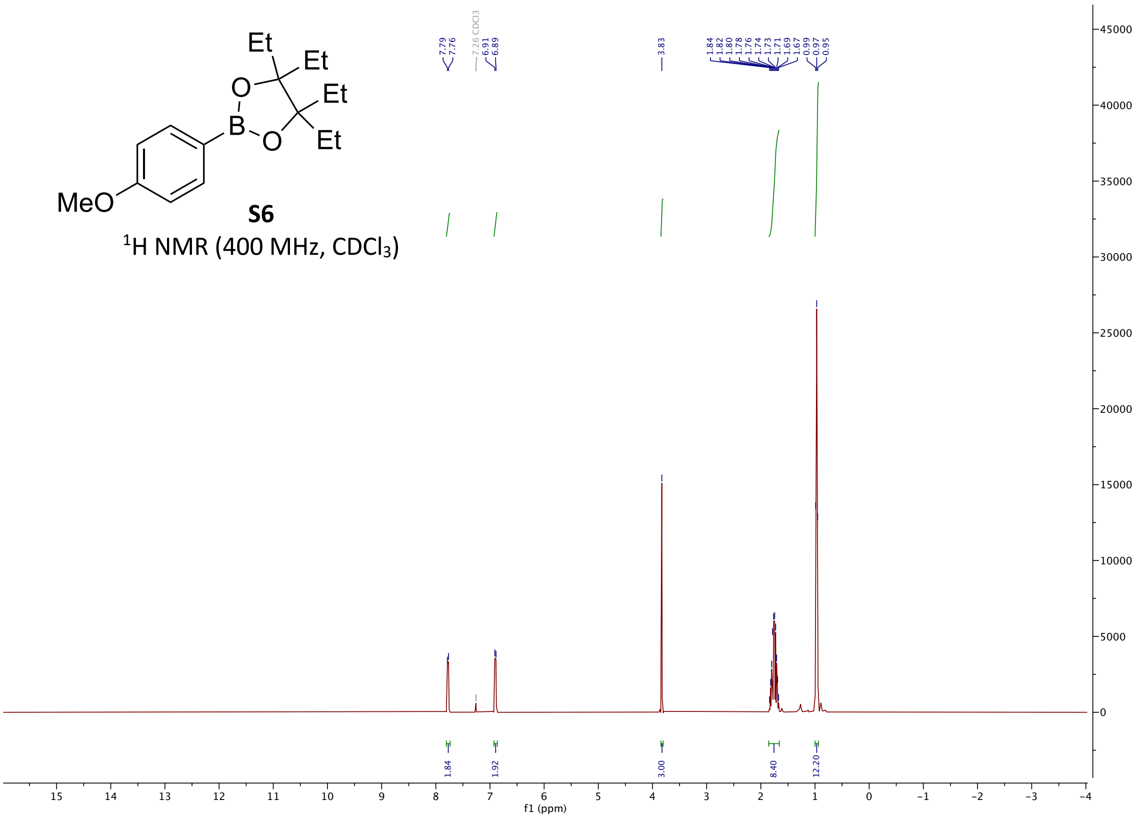

S6

^{1}H NMR (400 MHz, $CDCl_3$)

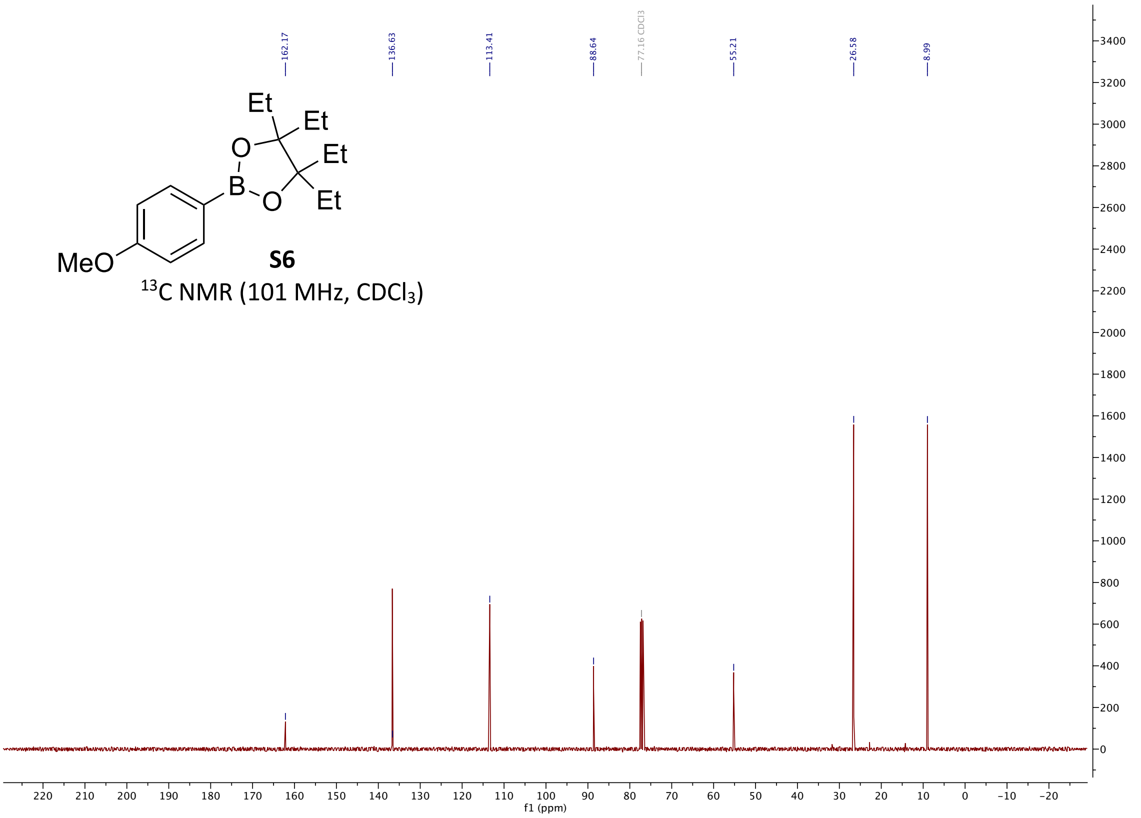

S6

^{13}C NMR (101 MHz, $CDCl_3$)

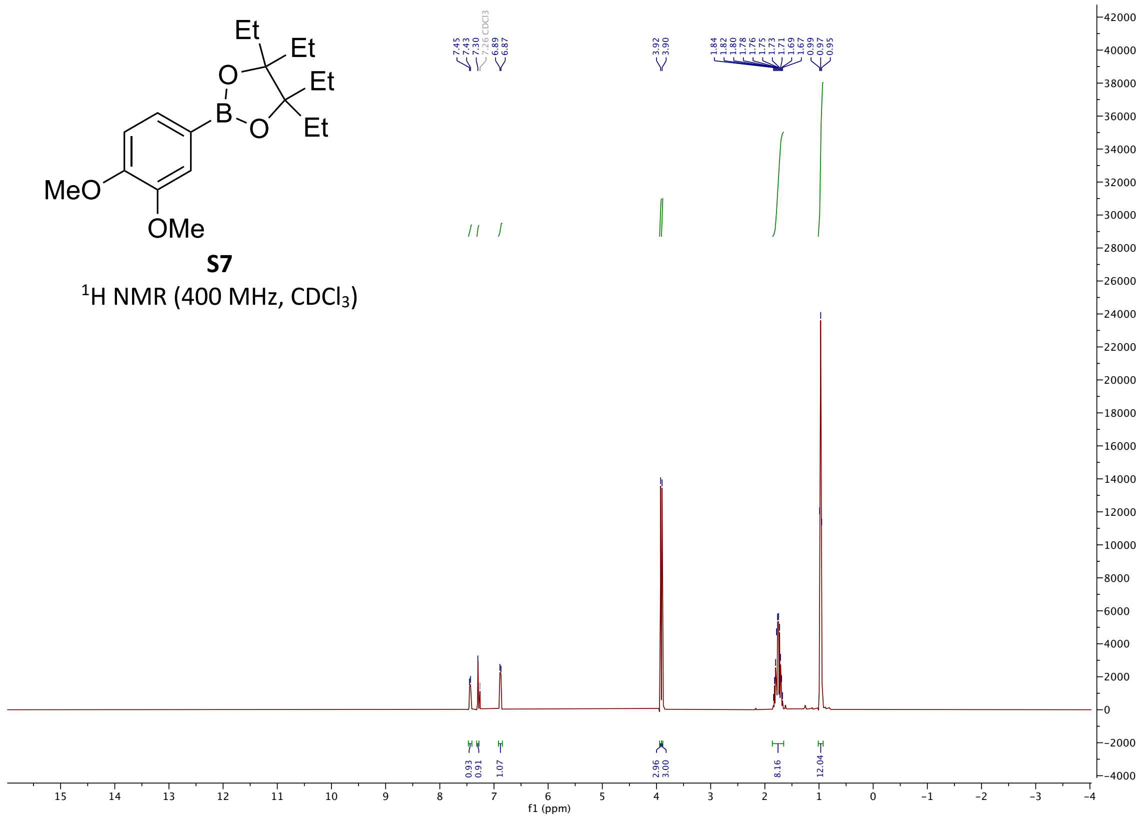
S7
^{1}H NMR (400 MHz, $CDCl_3$)

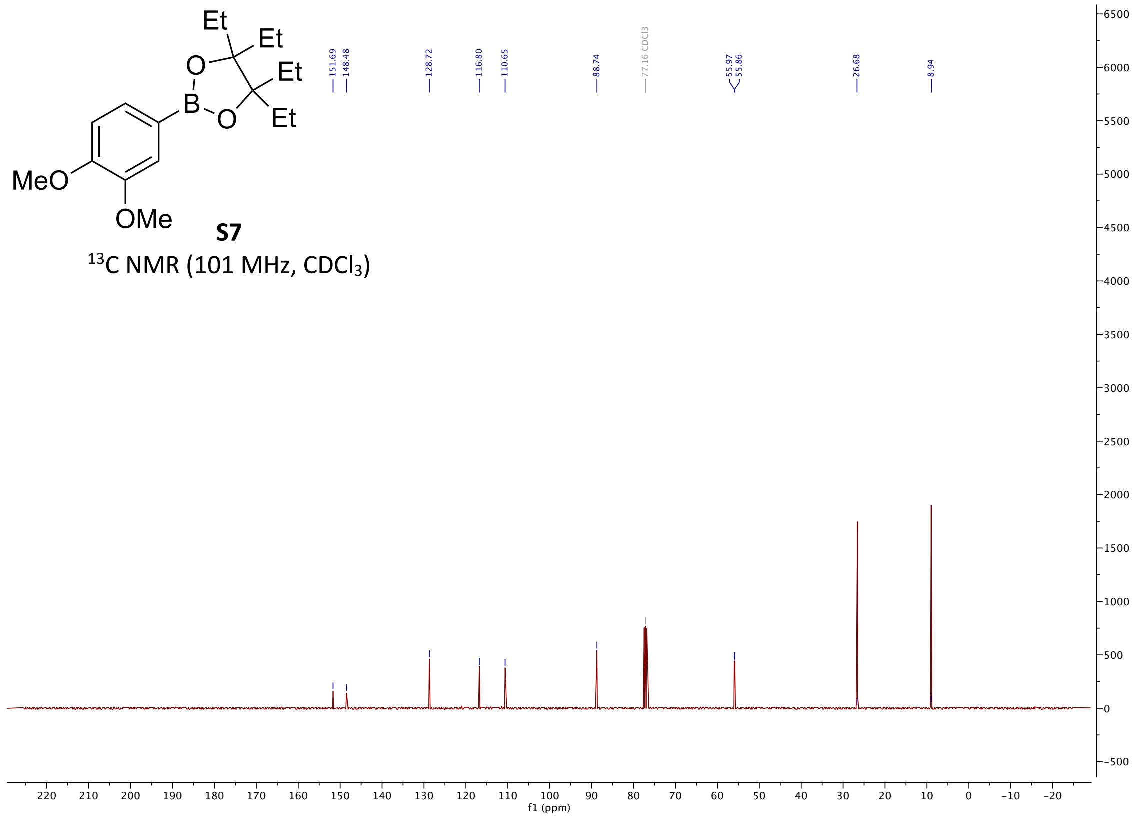
S7
^{13}C NMR (101 MHz, $CDCl_3$)

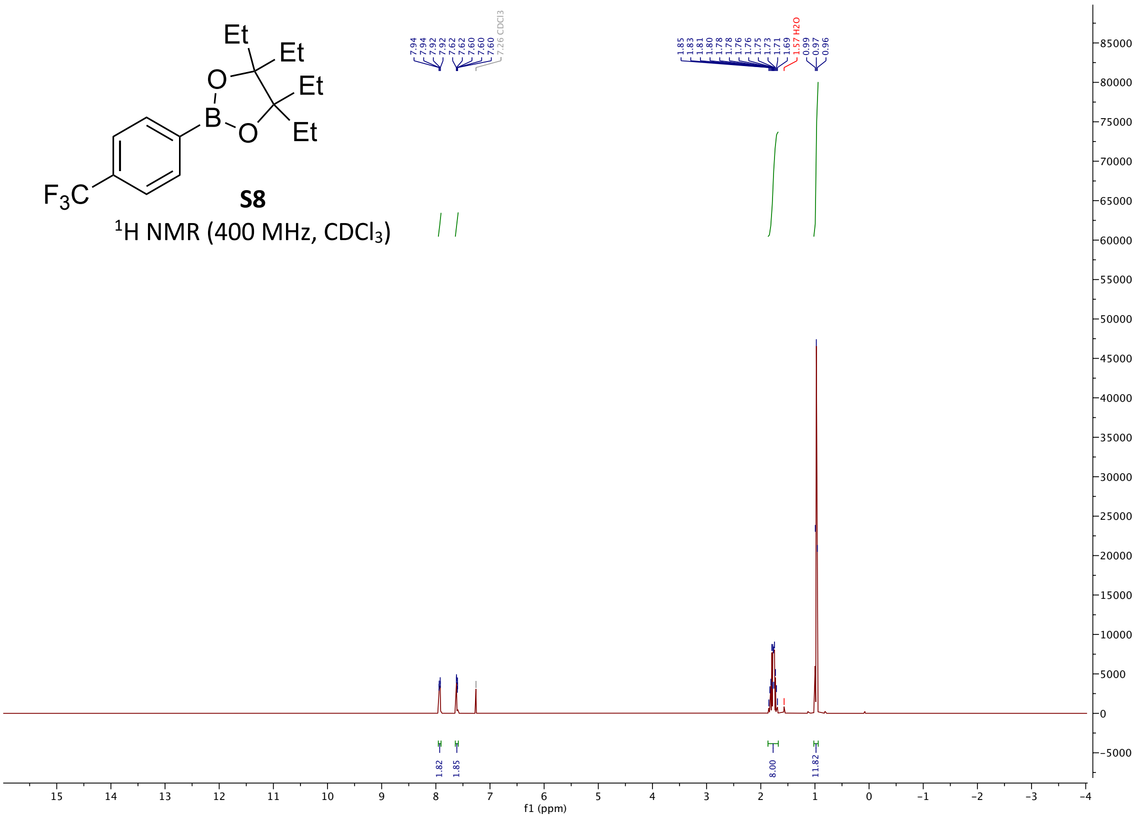
S8
^{1}H NMR (400 MHz, CDCl$_3$)

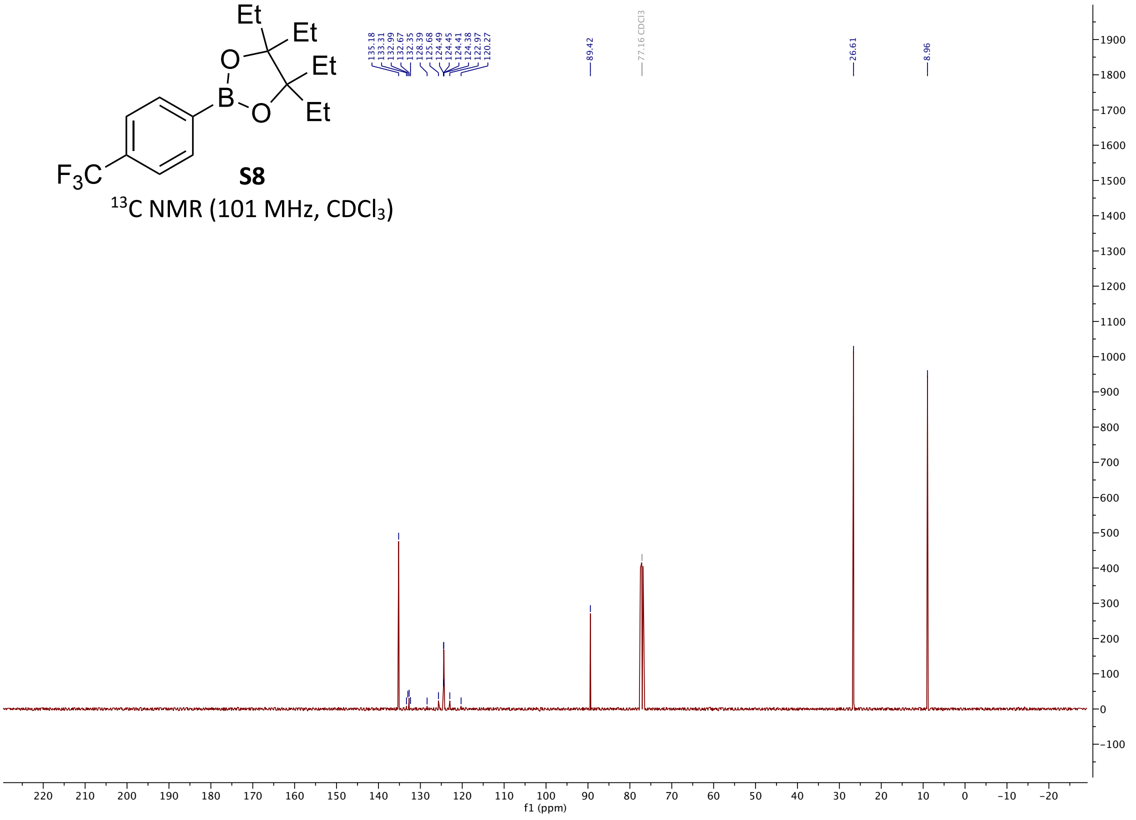
S8
^{13}C NMR (101 MHz, CDCl$_3$)

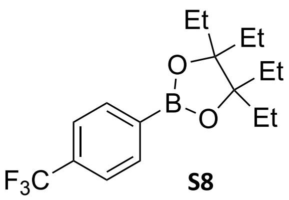

S8

^{19}F NMR (377 MHz, $CDCl_3$)

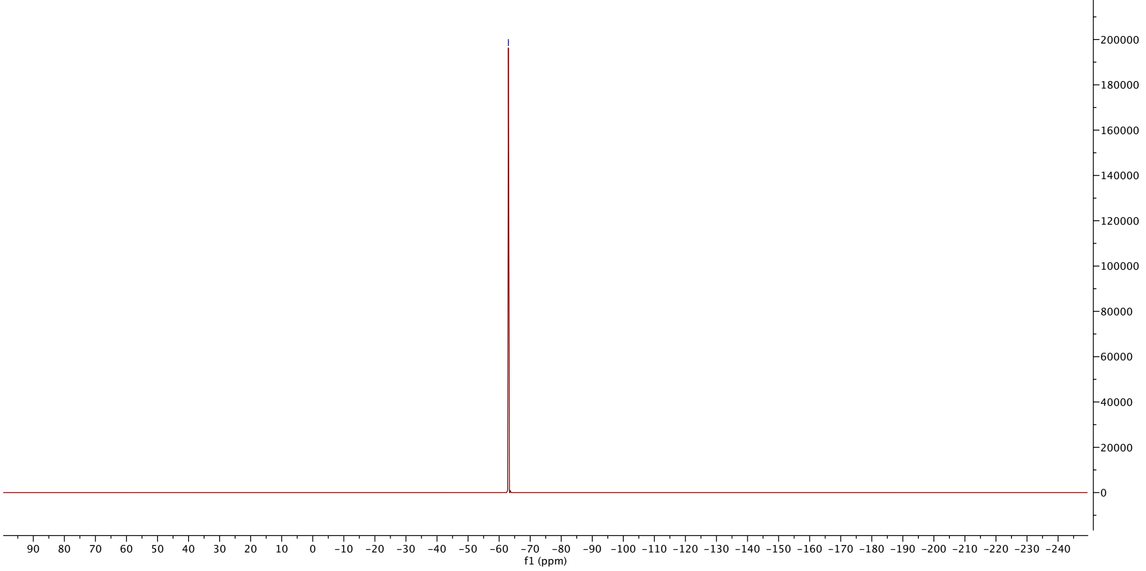

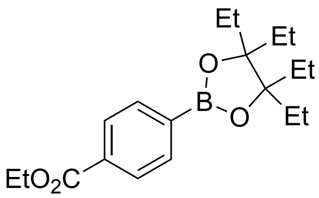

S9

^{1}H NMR (400 MHz, $CDCl_3$)

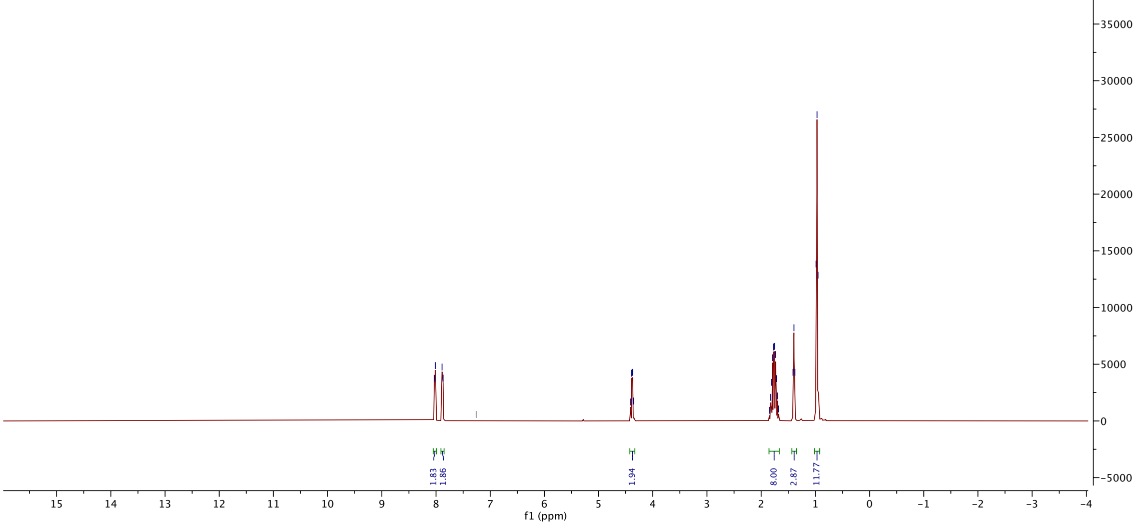

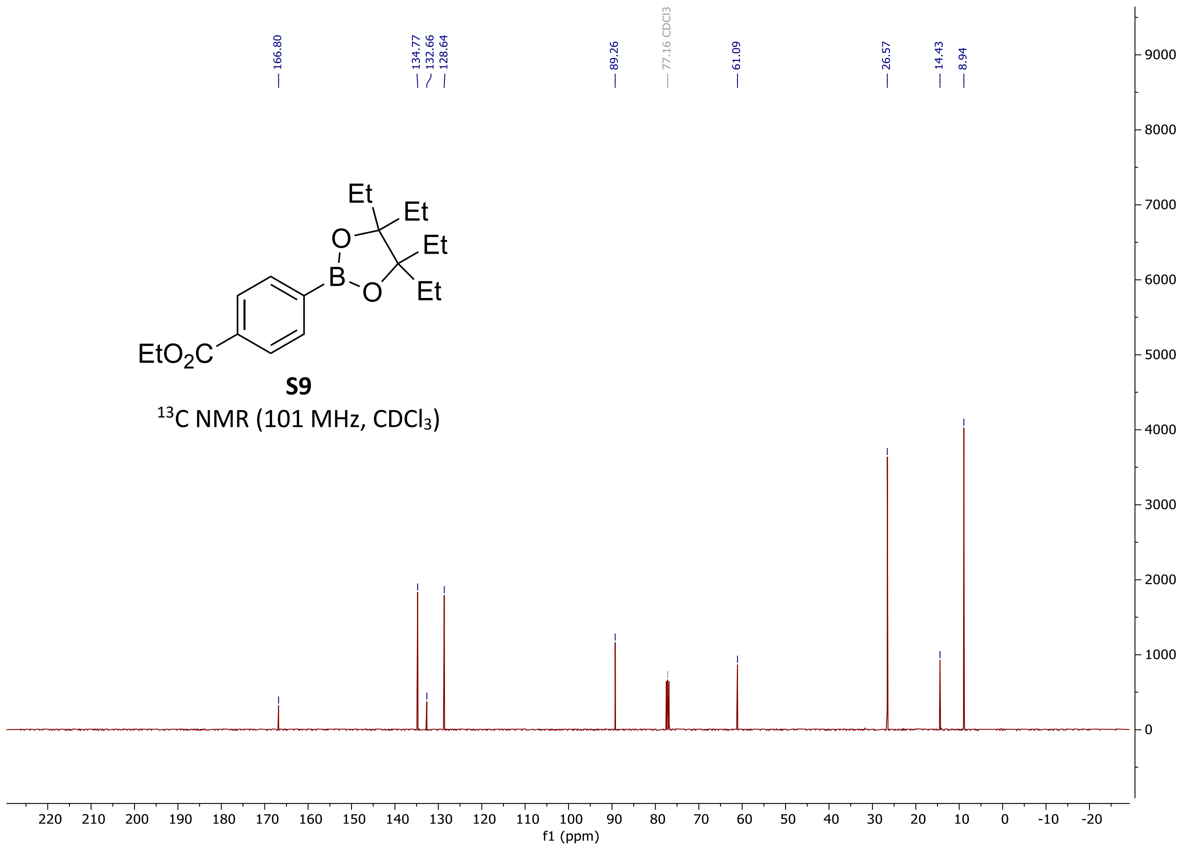
S9
^{13}C NMR (101 MHz, CDCl$_3$)

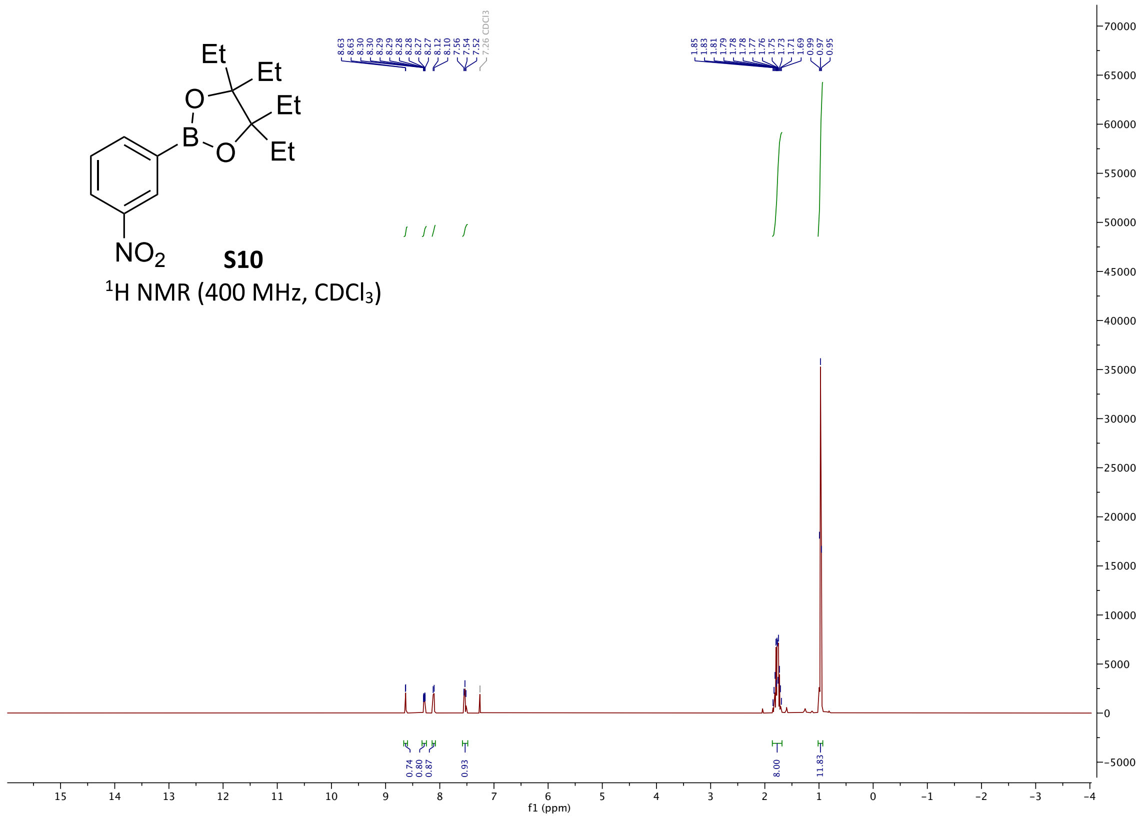
S10
^{1}H NMR (400 MHz, CDCl$_3$)

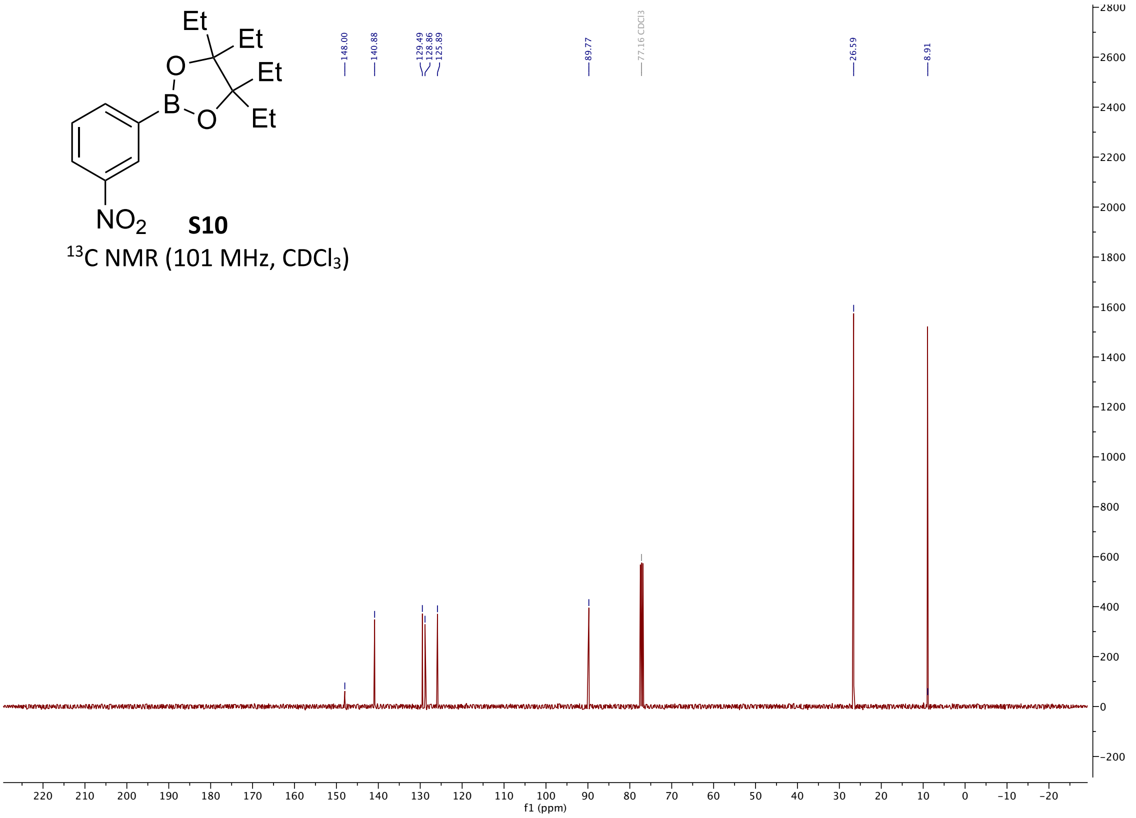
Et
Et
O
Et
B
O
Et
NO2
S10
13C NMR (101 MHz, CDCl3)
148.00
140.88
129.49
128.86
125.89
89.77
77.16 CDCl3
26.59
8.91
f1 (ppm)

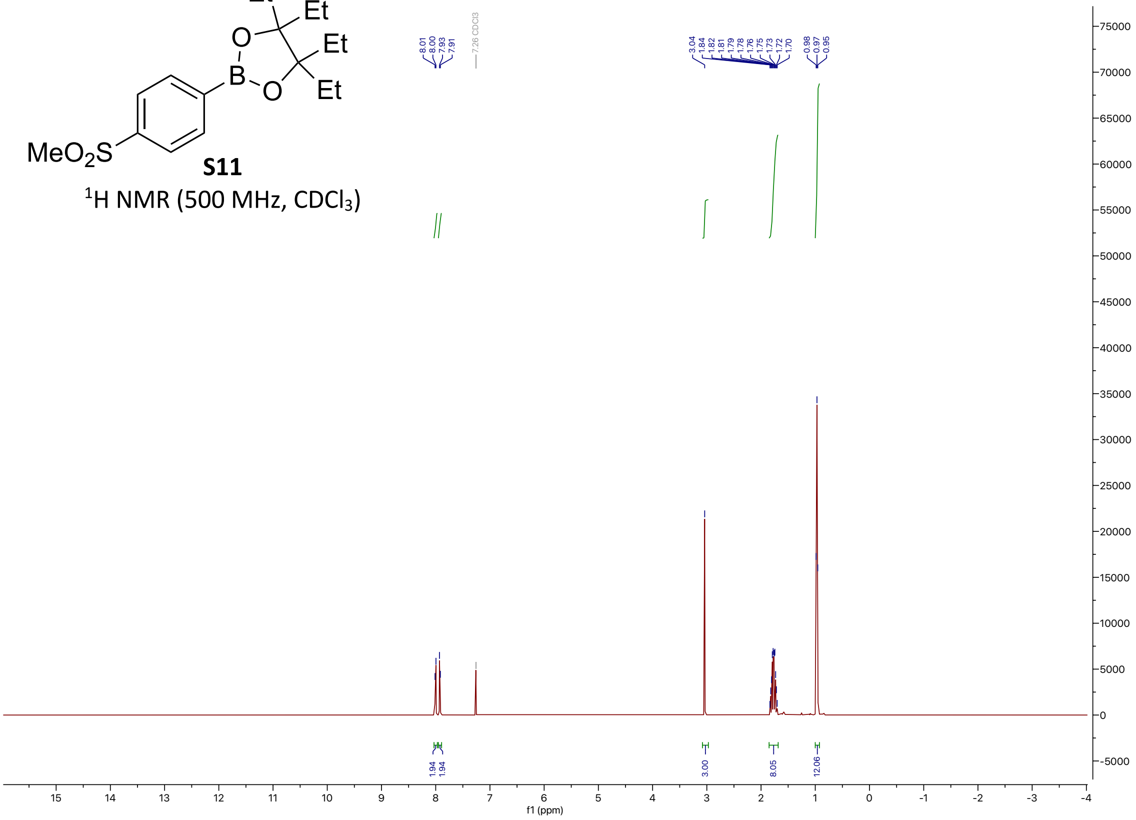
Et
Et
O
Et
B
O
Et
MeO2S
S11
1H NMR (500 MHz, CDCl3)
8.01
8.00
7.93
7.91
7.26 CDCl3
3.04
1.84
1.82
1.81
1.79
1.78
1.76
1.75
1.73
1.72
1.70
0.98
0.97
0.95
1.94
1.94
3.00
8.05
12.06
f1 (ppm)

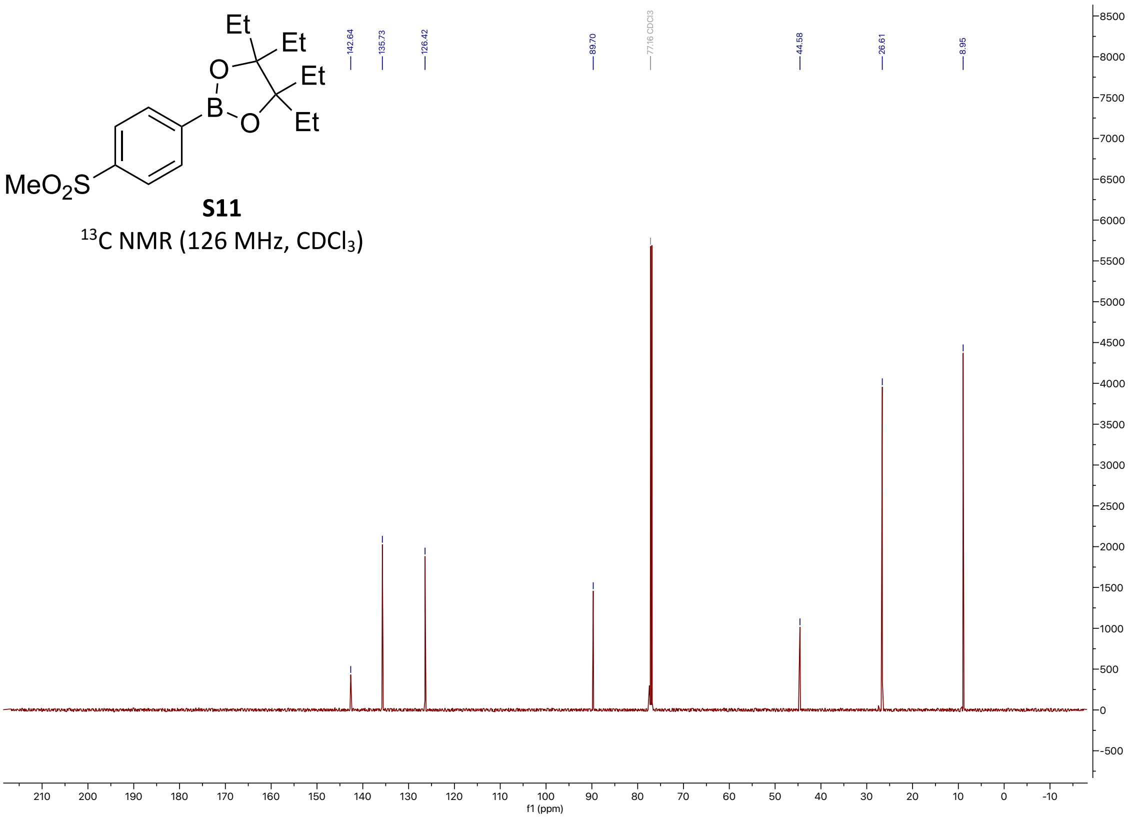

S11

^{13}C NMR (126 MHz, $CDCl_3$)

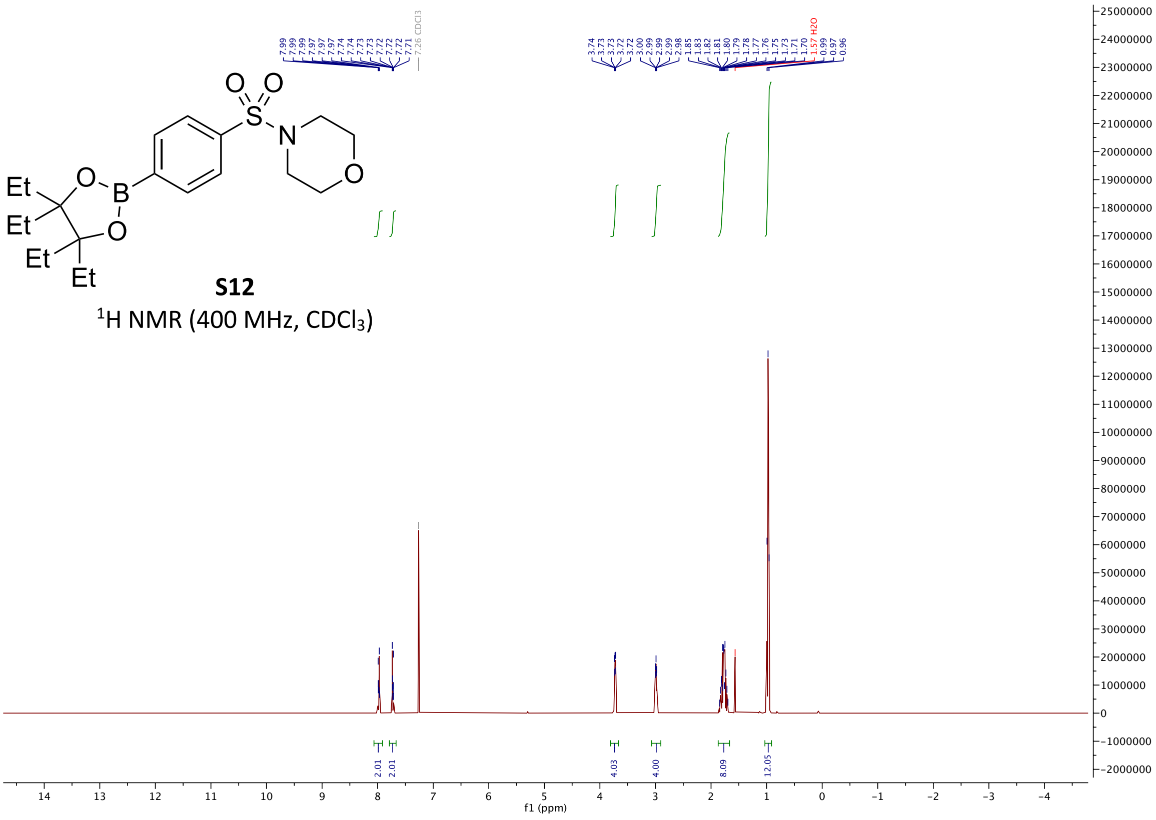

S12

^{1}H NMR (400 MHz, $CDCl_3$)

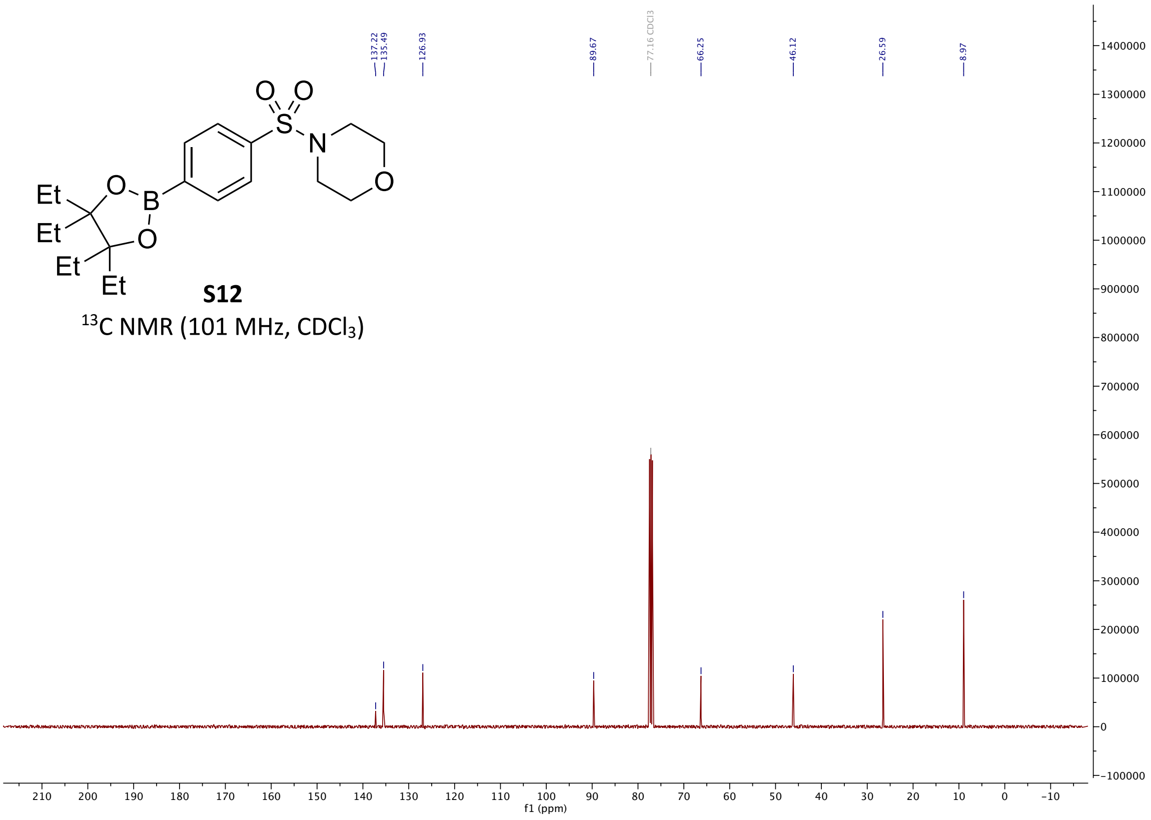

S12

^{13}C NMR (101 MHz, $CDCl_3$)

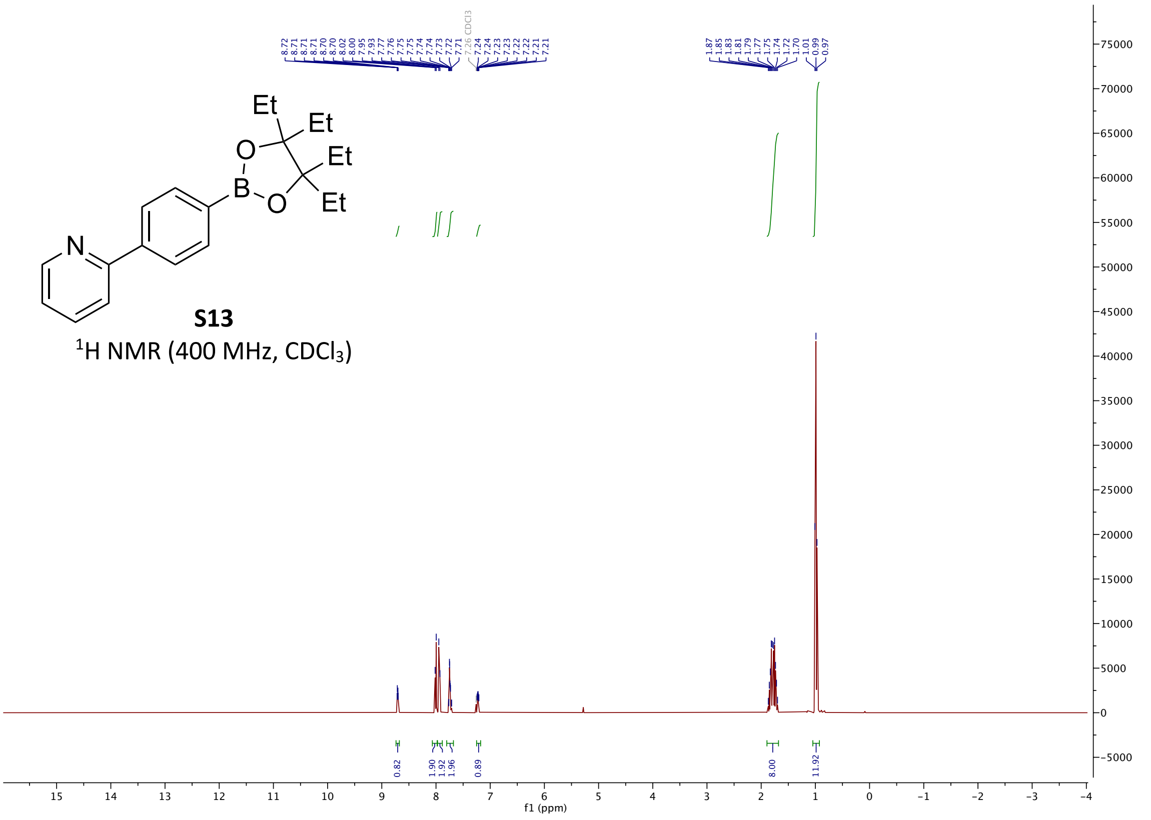

S13

^{1}H NMR (400 MHz, $CDCl_3$)

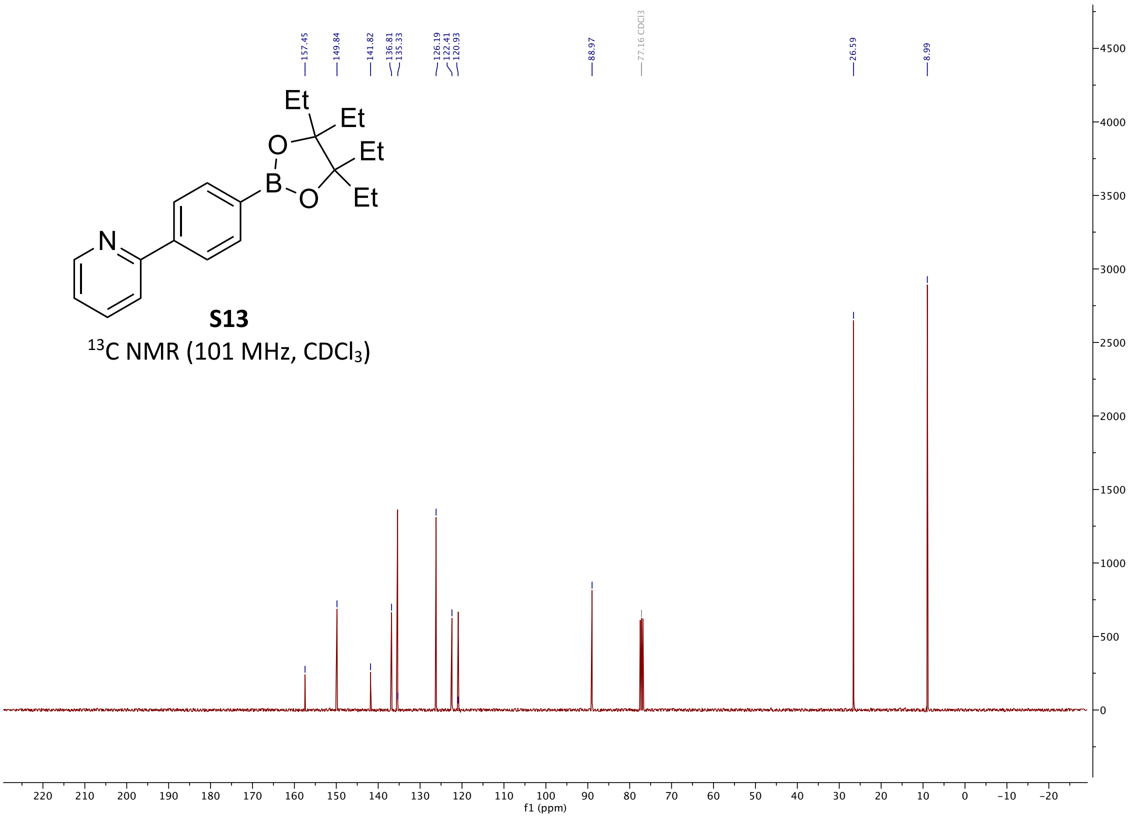

S13

^{13}C NMR (101 MHz, $CDCl_3$)

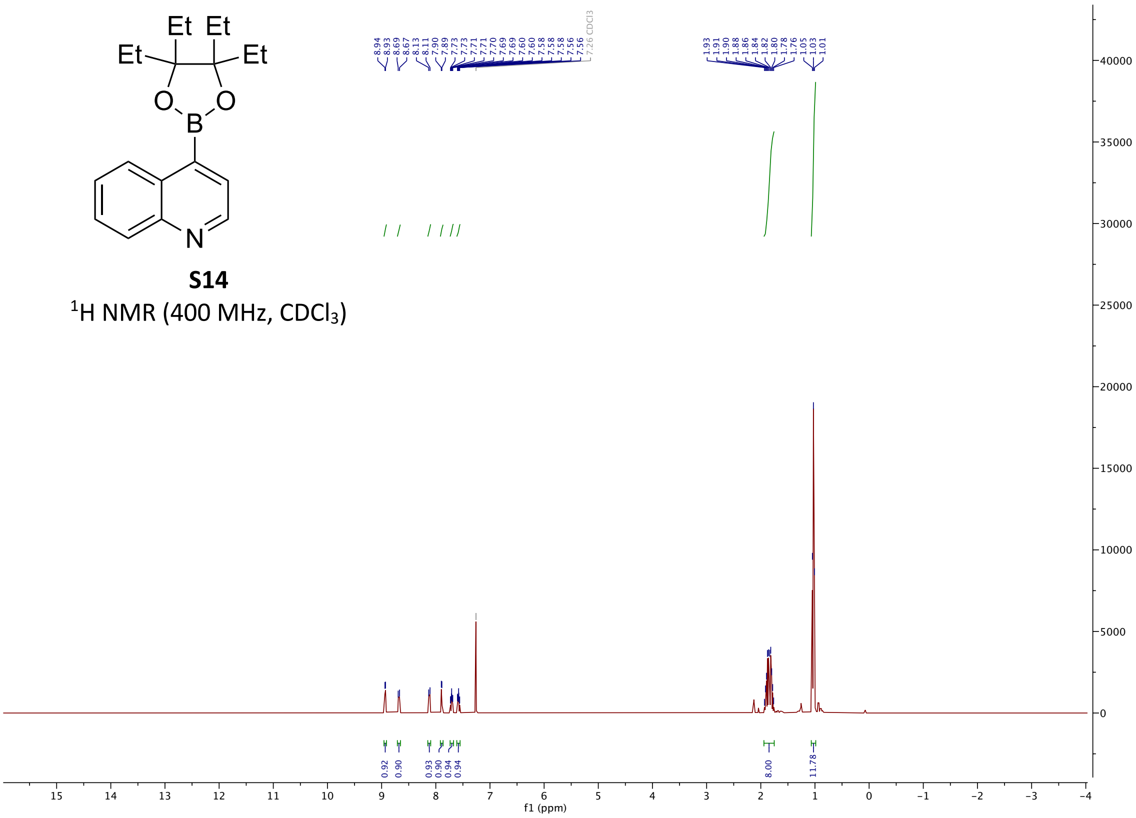

S14

^{1}H NMR (400 MHz, $CDCl_3$)

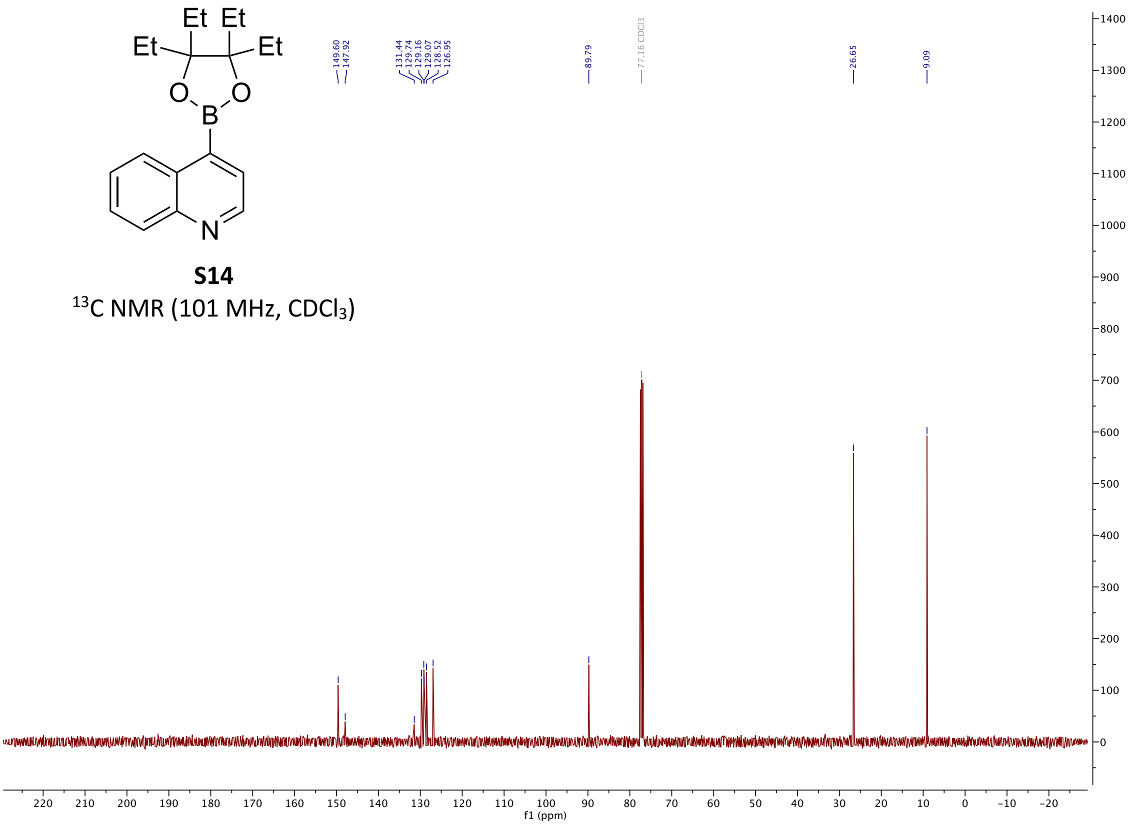

S14

^{13}C NMR (101 MHz, $CDCl_3$)

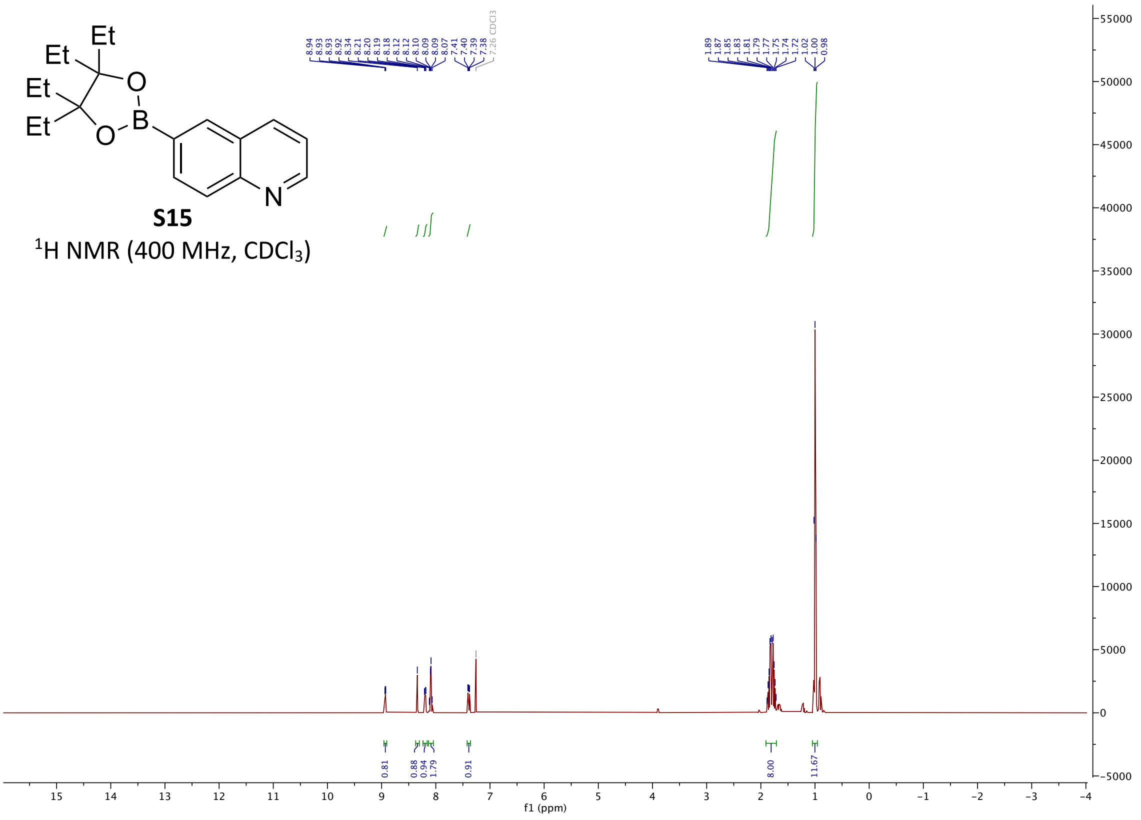

S15

^{1}H NMR (400 MHz, $CDCl_3$)

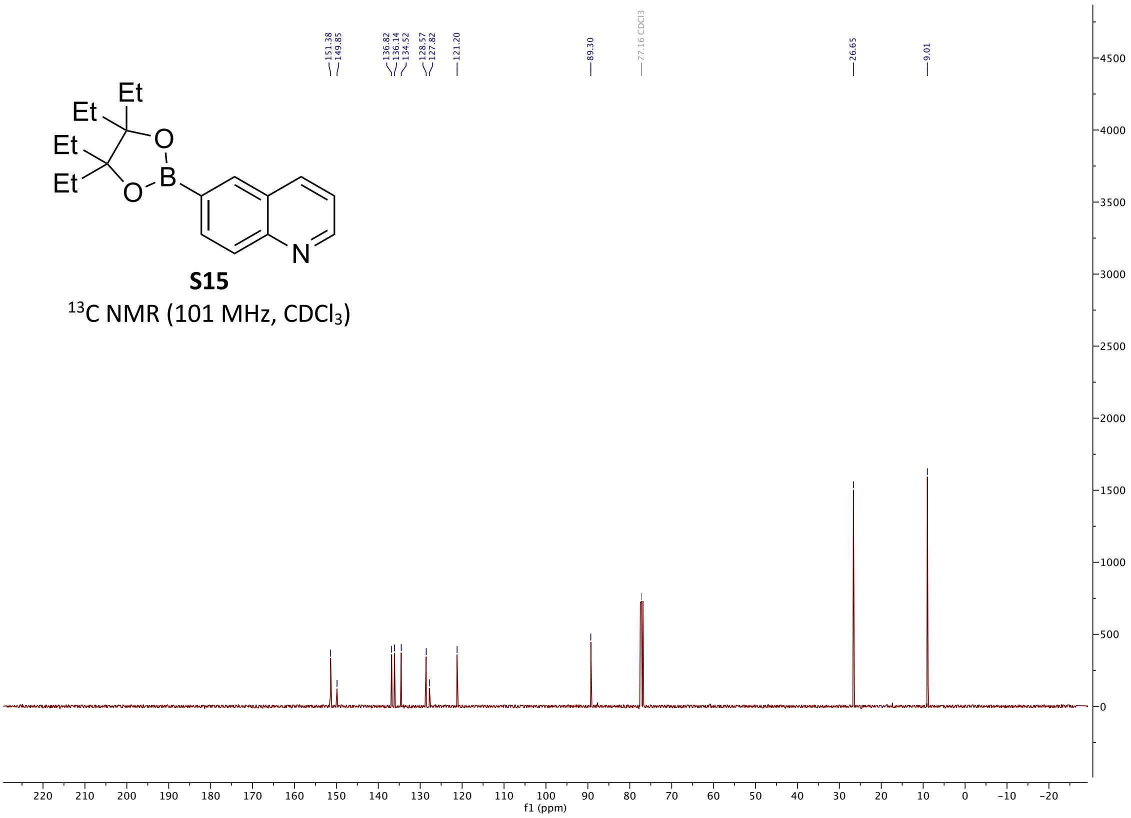

S15

^{13}C NMR (101 MHz, $CDCl_3$)

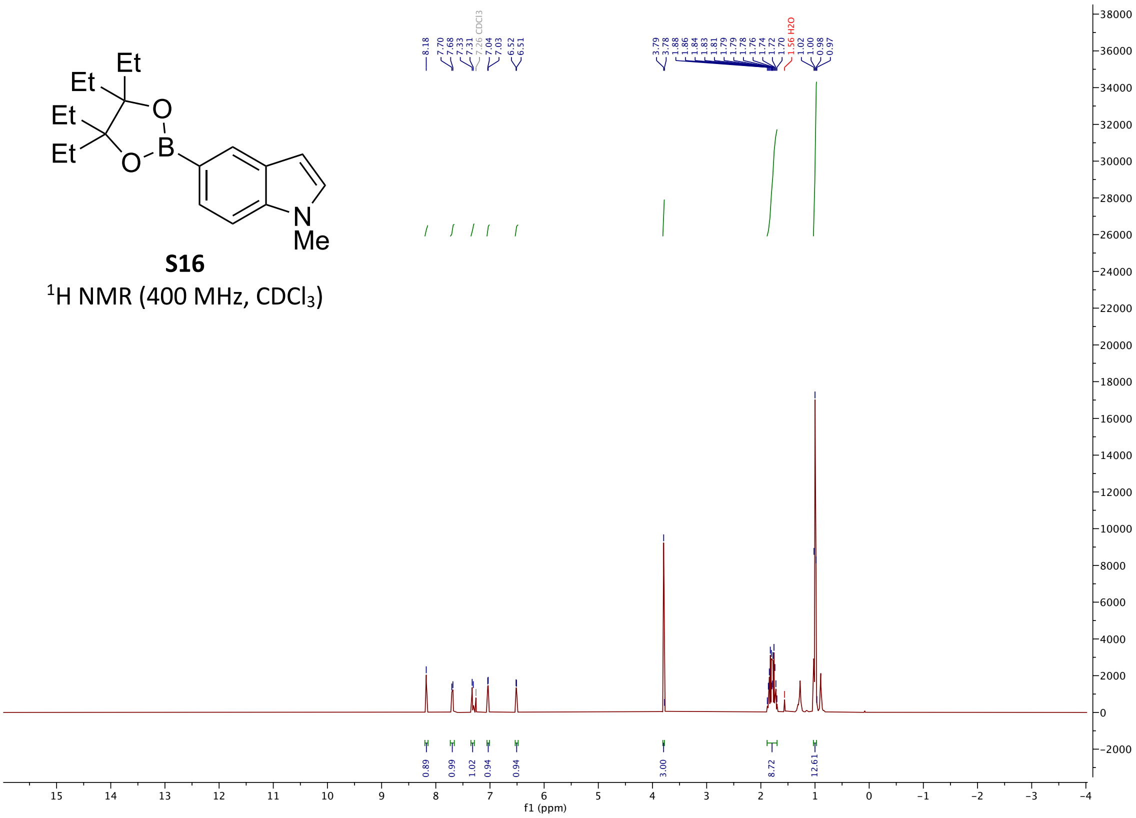

S16

^{1}H NMR (400 MHz, $CDCl_3$)

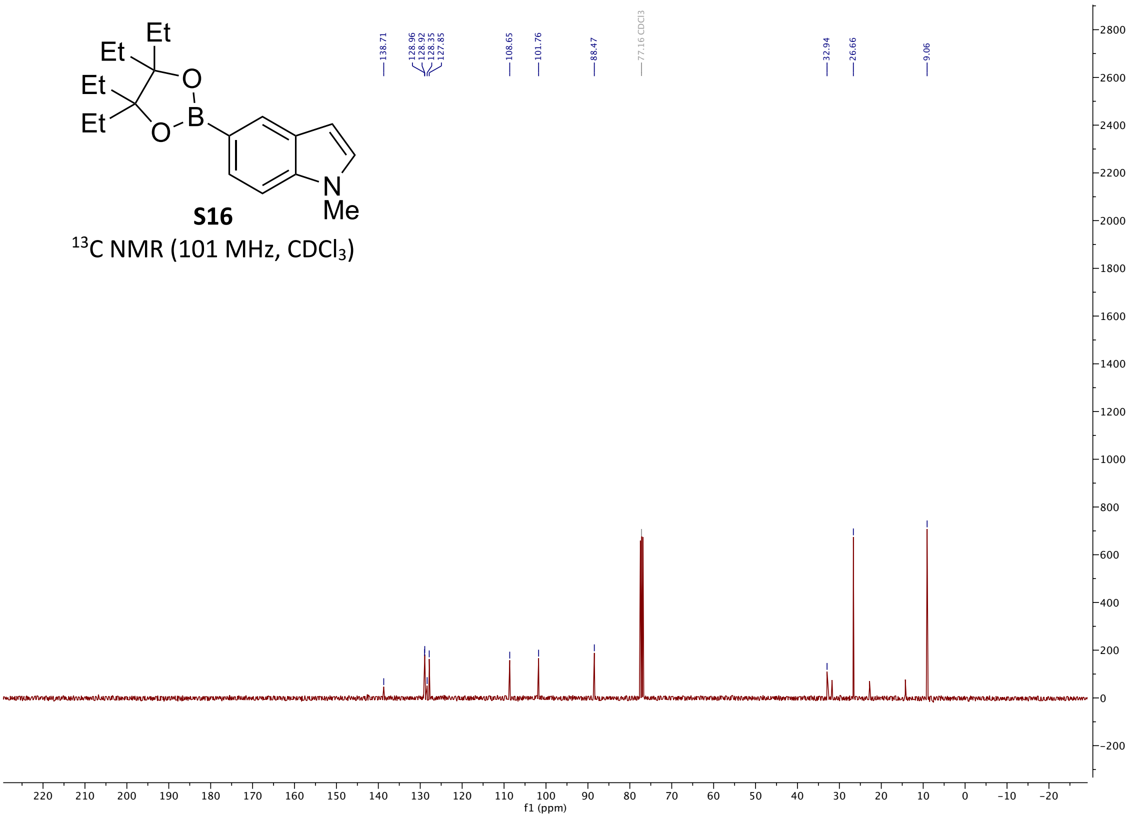

S16

^{13}C NMR (101 MHz, $CDCl_3$)

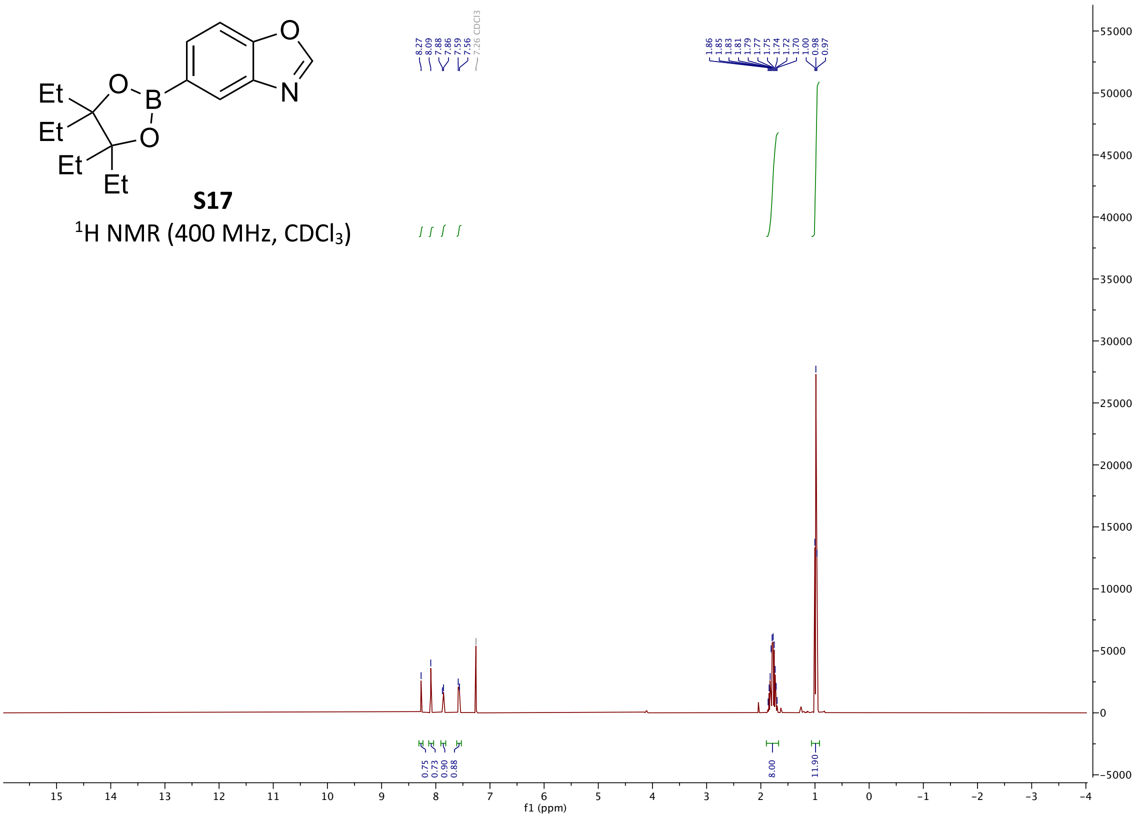

S17

^{1}H NMR (400 MHz, $CDCl_3$)

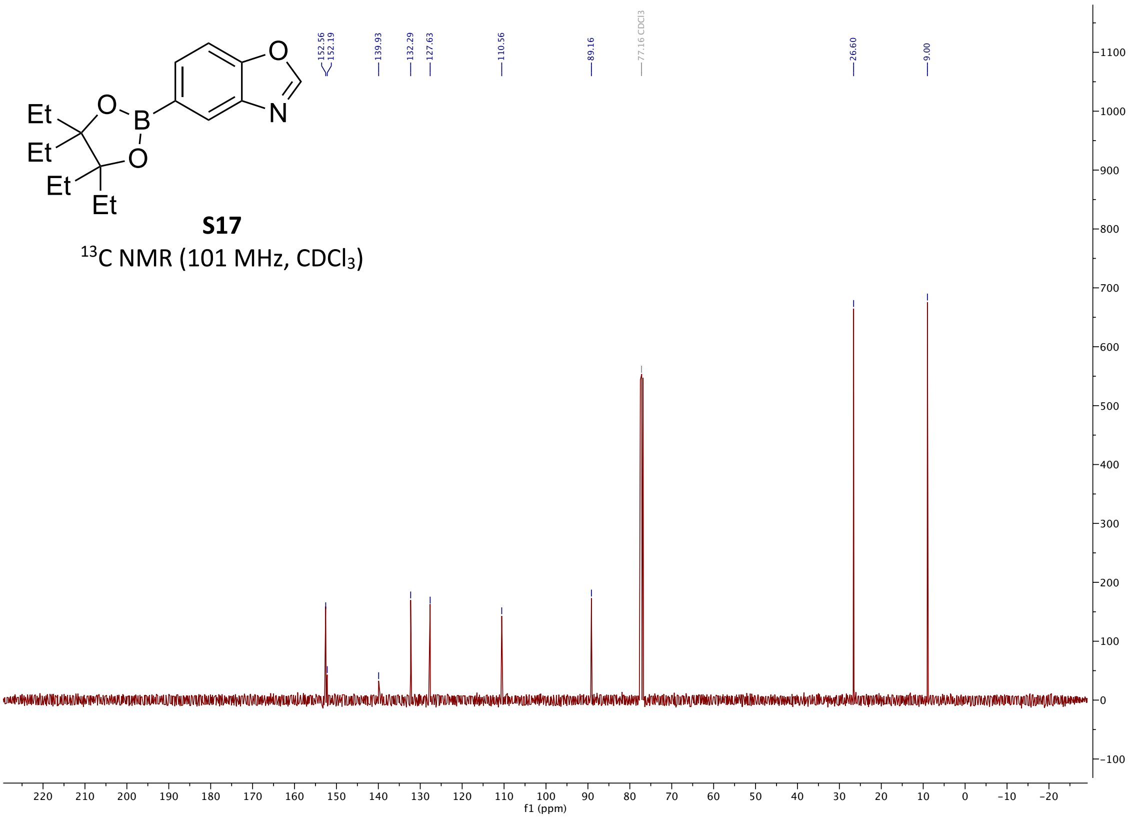

S17

^{13}C NMR (101 MHz, $CDCl_3$)

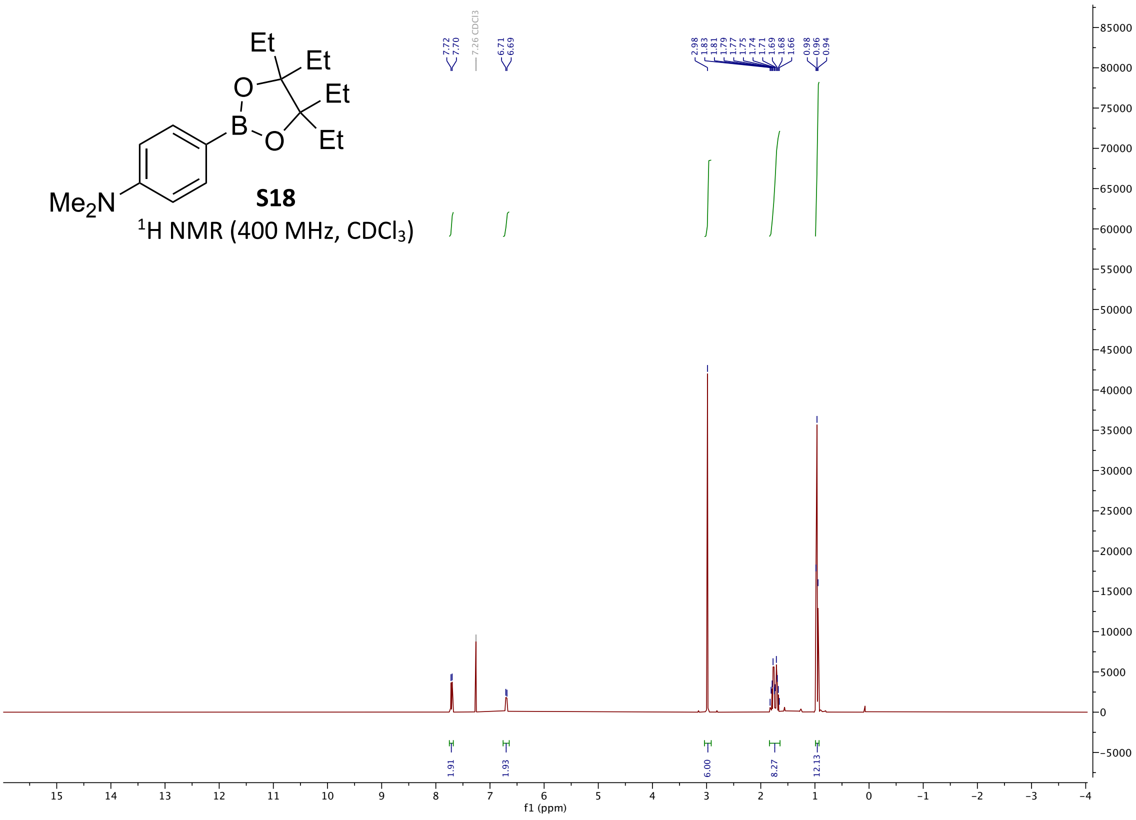

S18

^{1}H NMR (400 MHz, $CDCl_3$)

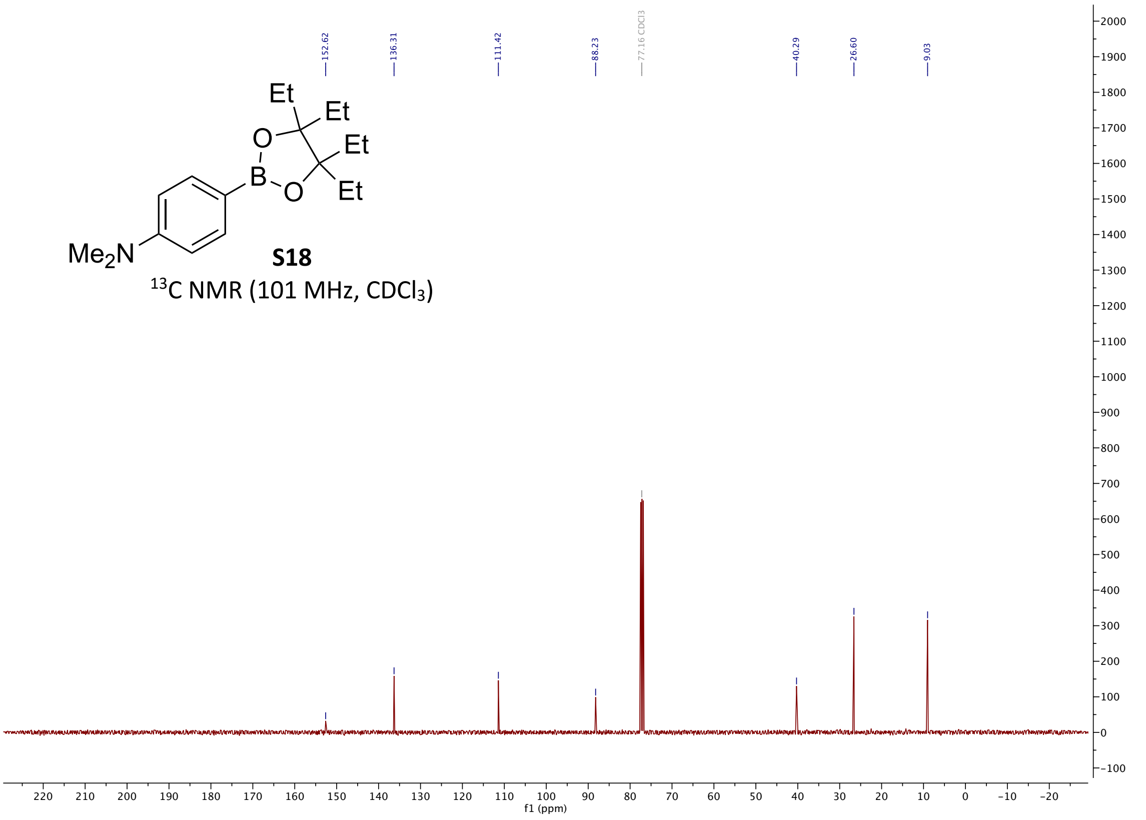

S18

^{13}C NMR (101 MHz, $CDCl_3$)

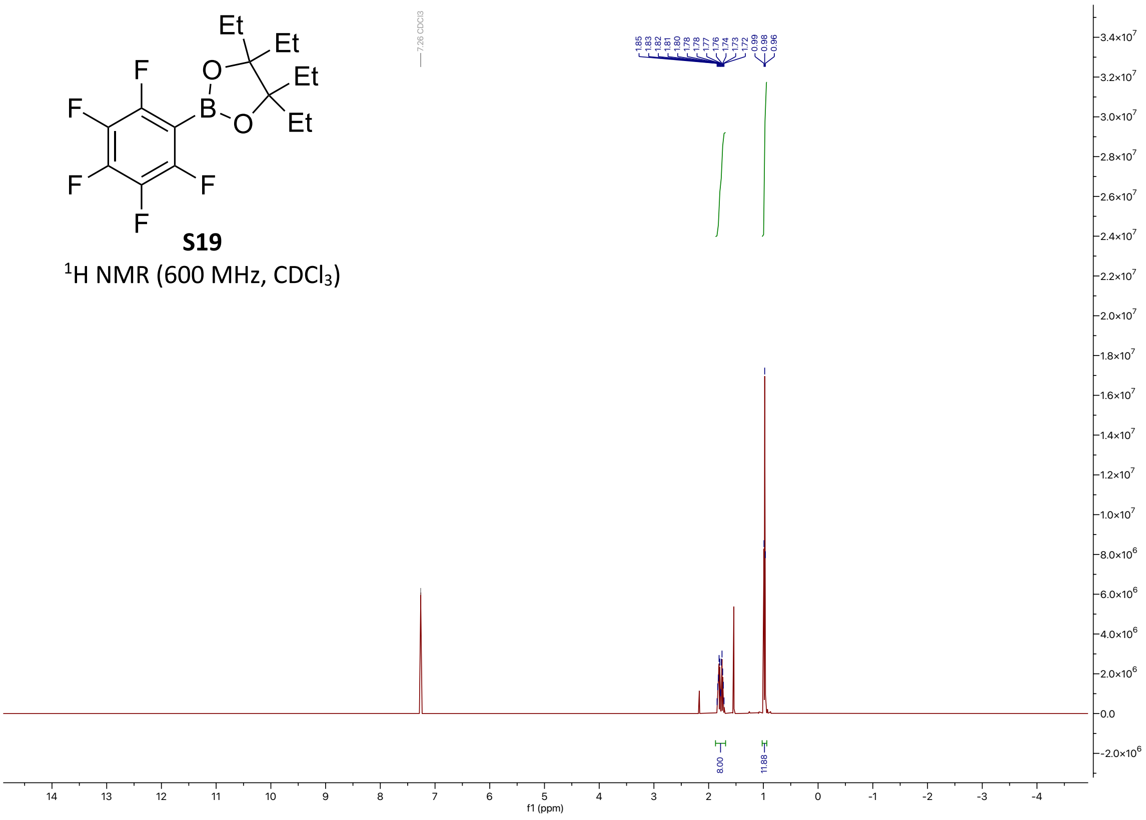

S19

^{1}H NMR (600 MHz, $CDCl_3$)

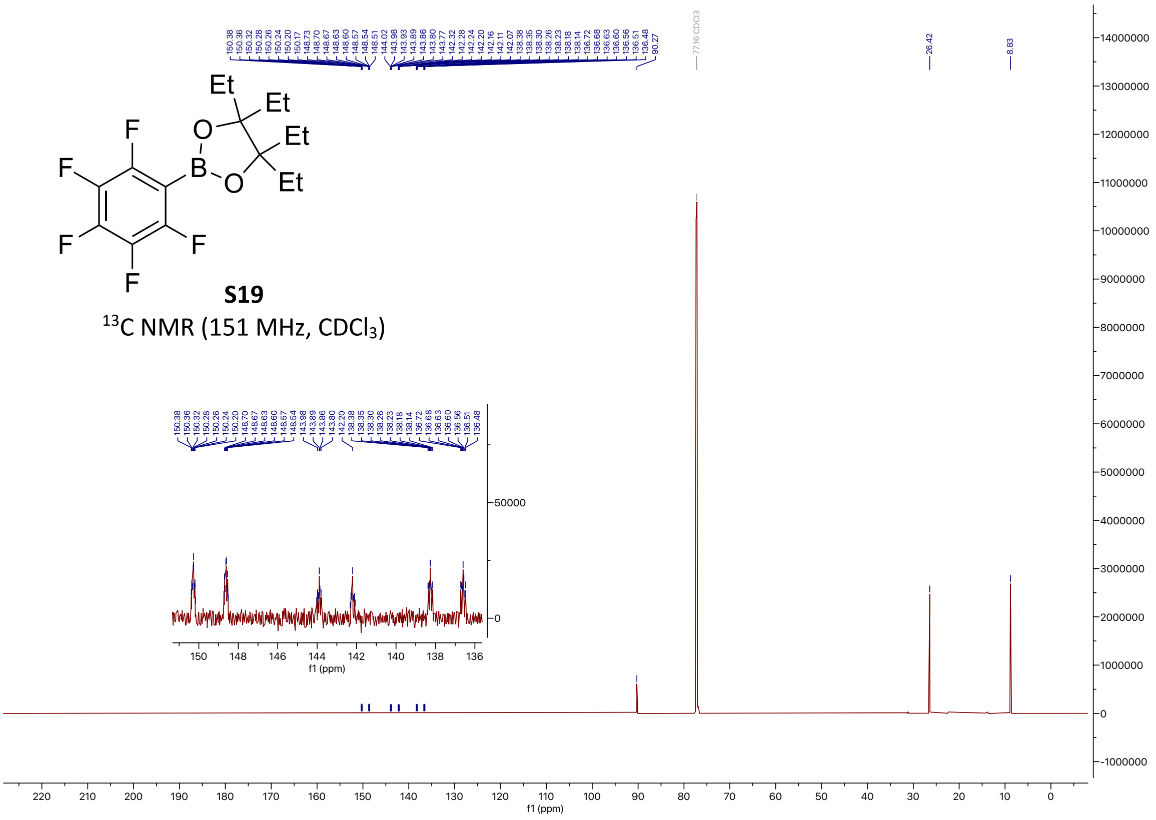
S19
^{13}C NMR (151 MHz, CDCl$_3$)
f1 (ppm)

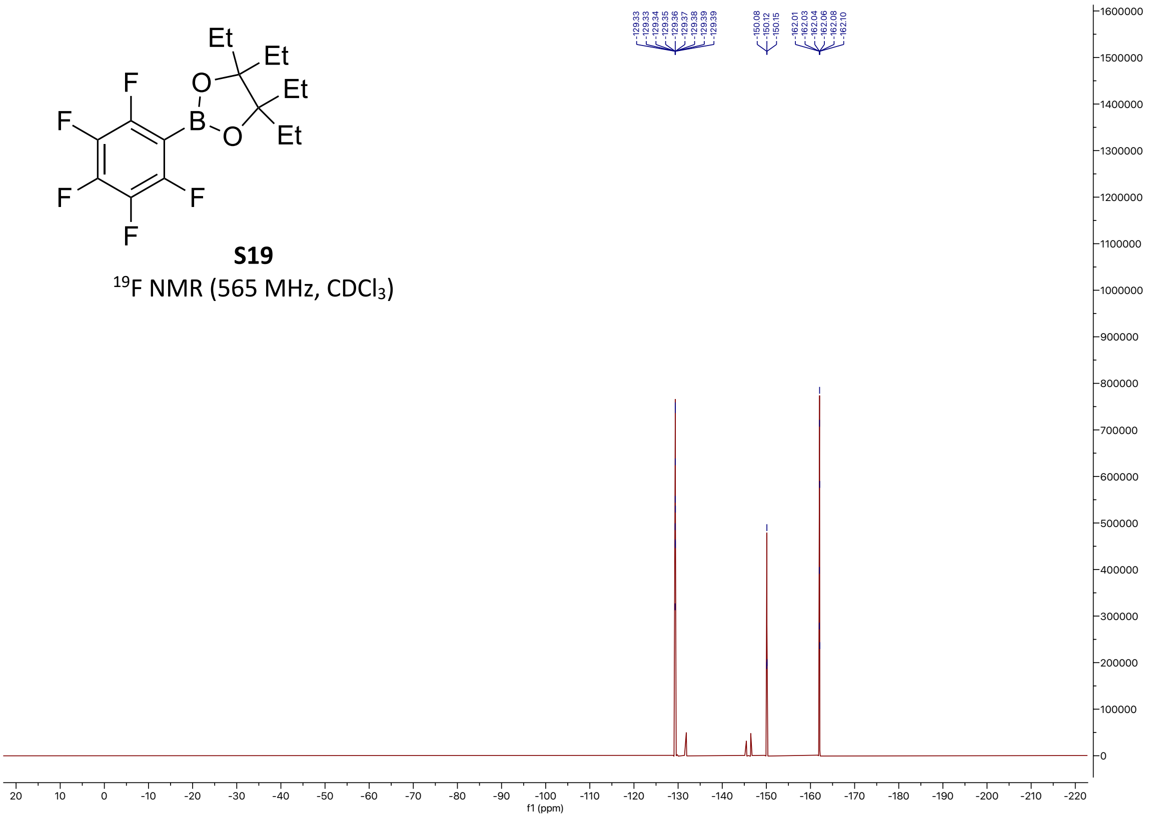
S19
^{19}F NMR (565 MHz, CDCl$_3$)
f1 (ppm)

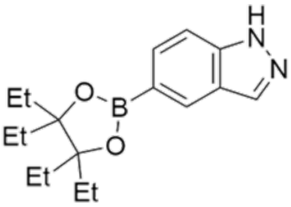

S21

^{1}H NMR (400 MHz, $CDCl_3$)

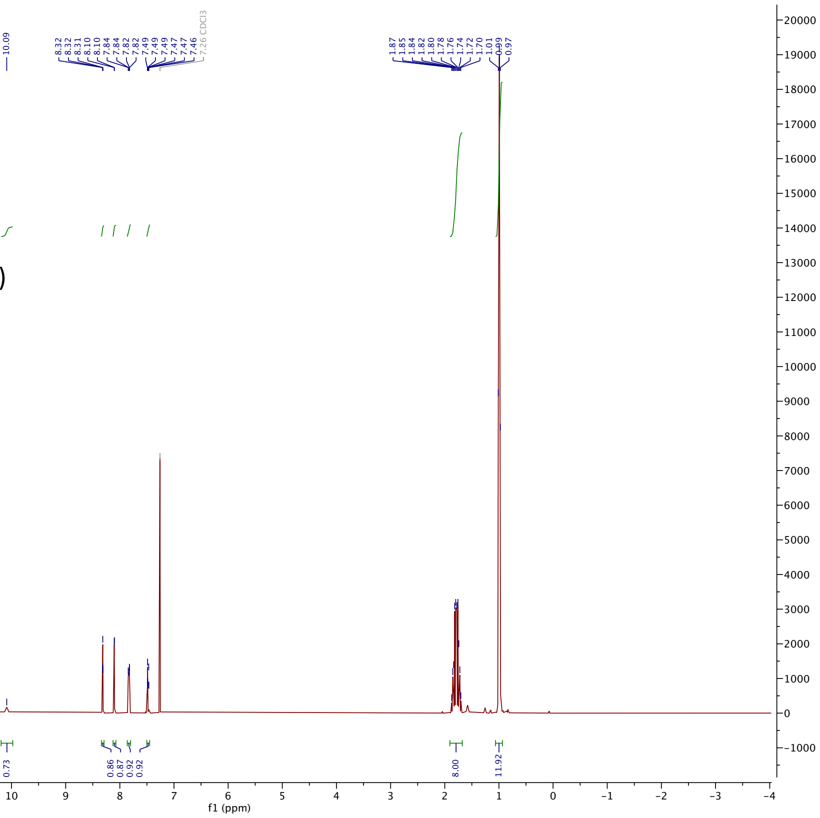

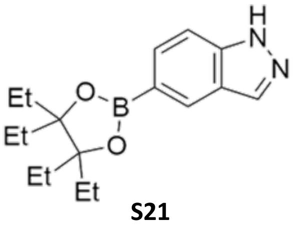

S21

^{13}C NMR (101 MHz, $CDCl_3$)

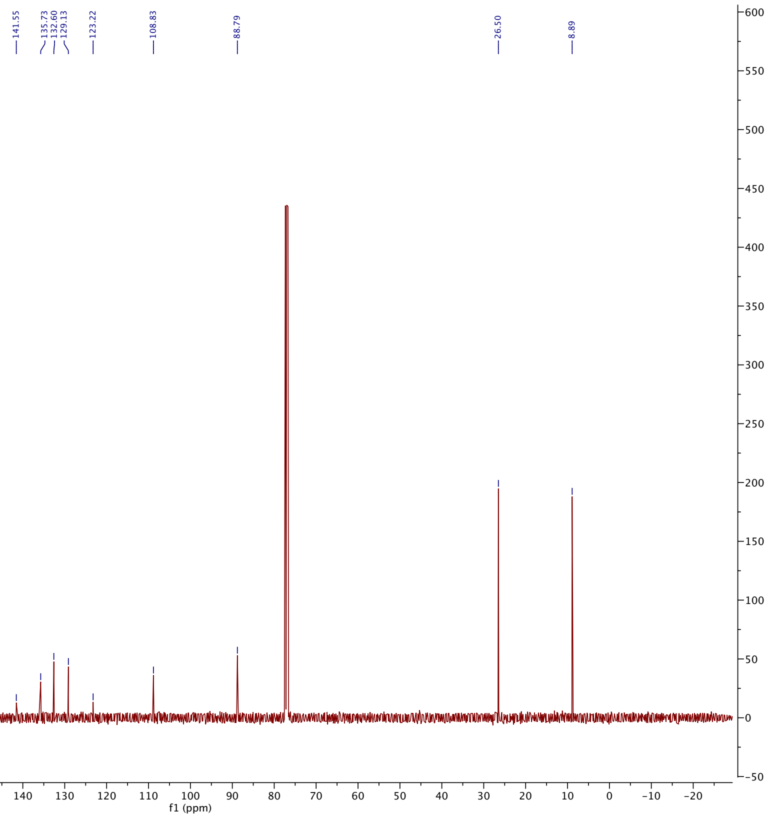

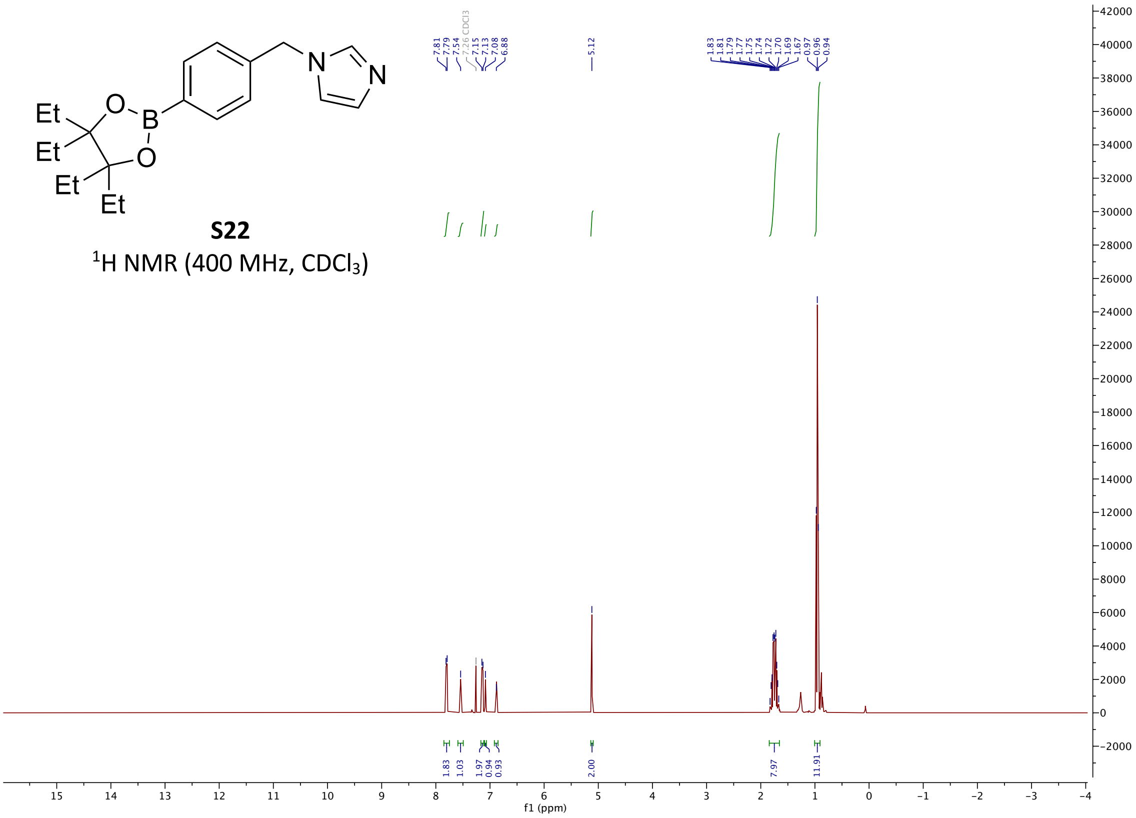

S22
^{1}H NMR (400 MHz, $CDCl_3$)

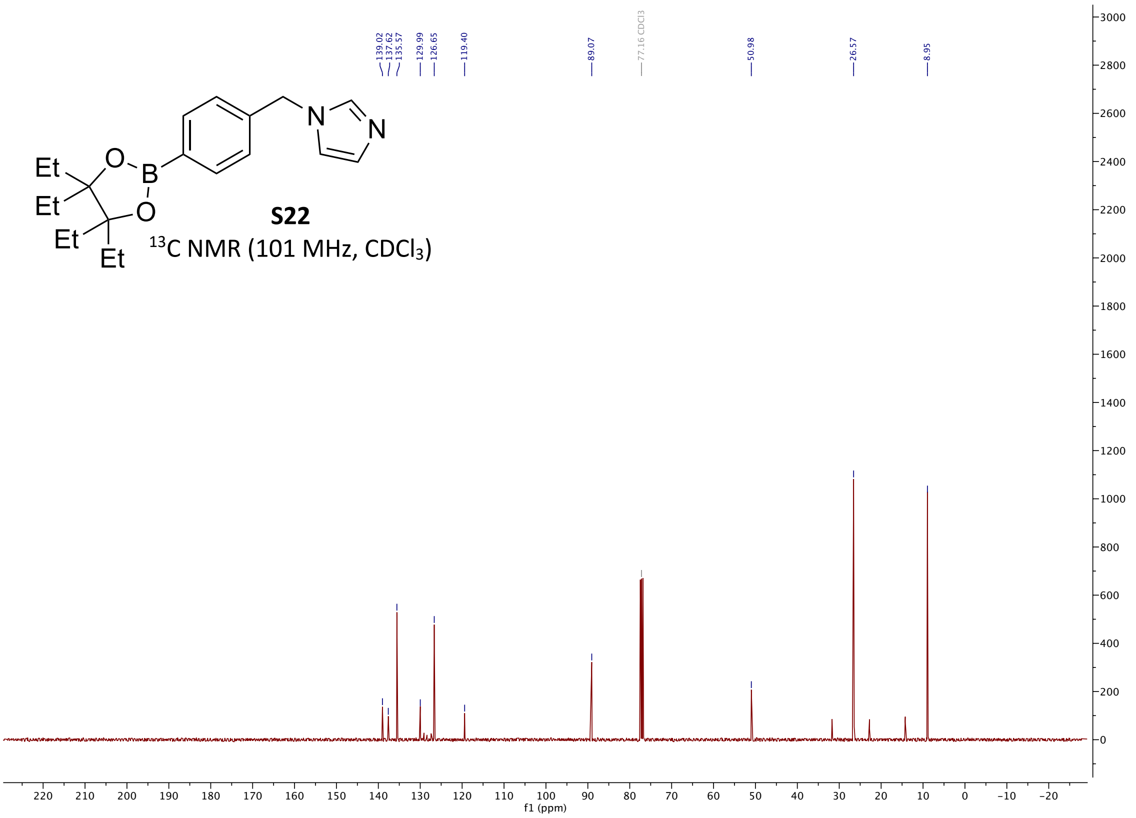

S22
^{13}C NMR (101 MHz, $CDCl_3$)

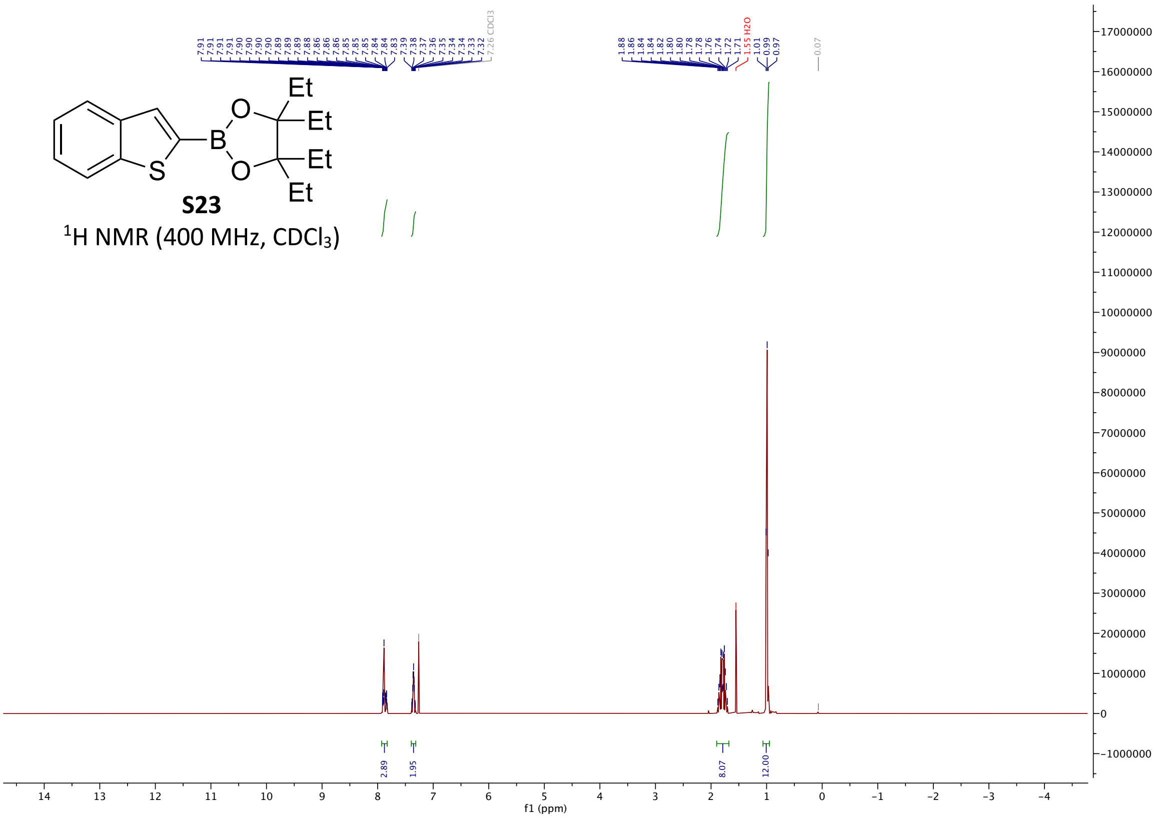

S23

^{1}H NMR (400 MHz, $CDCl_3$)

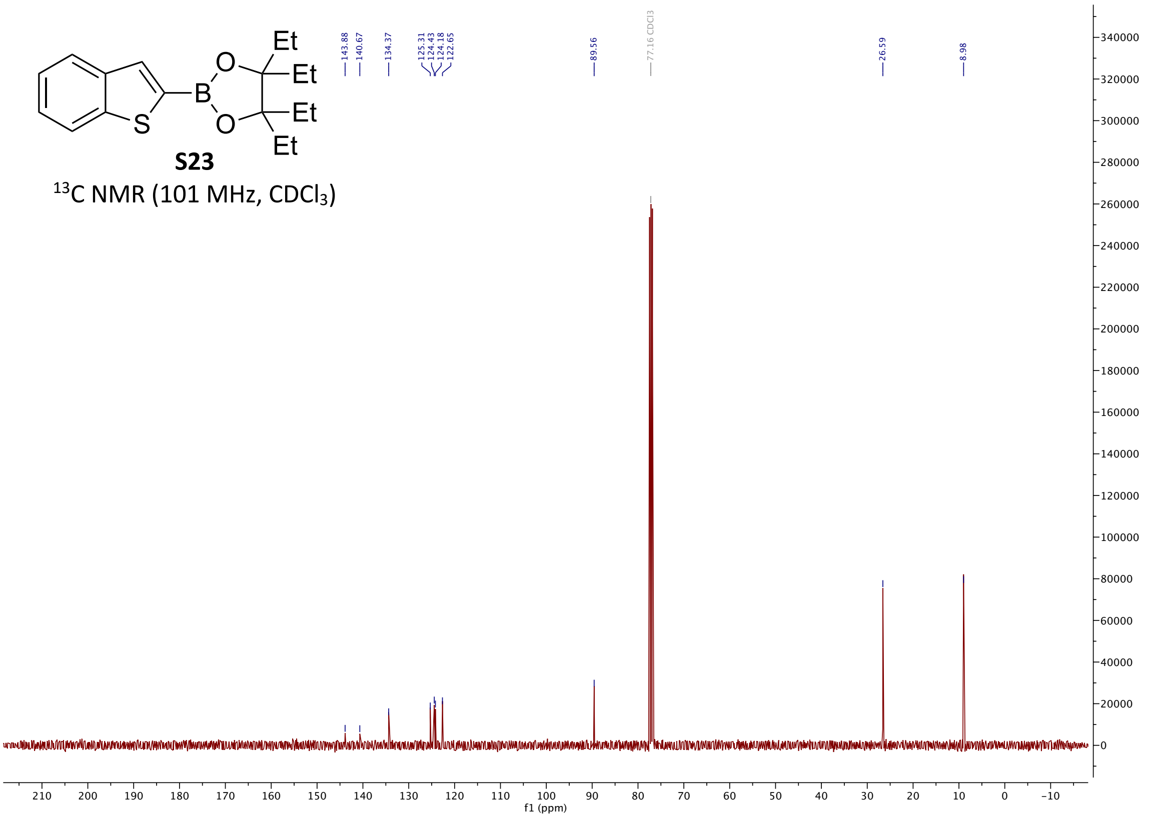

S23

^{13}C NMR (101 MHz, $CDCl_3$)

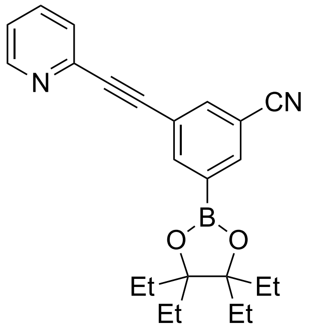

S24

^{1}H NMR (500 MHz, $CDCl_3$)

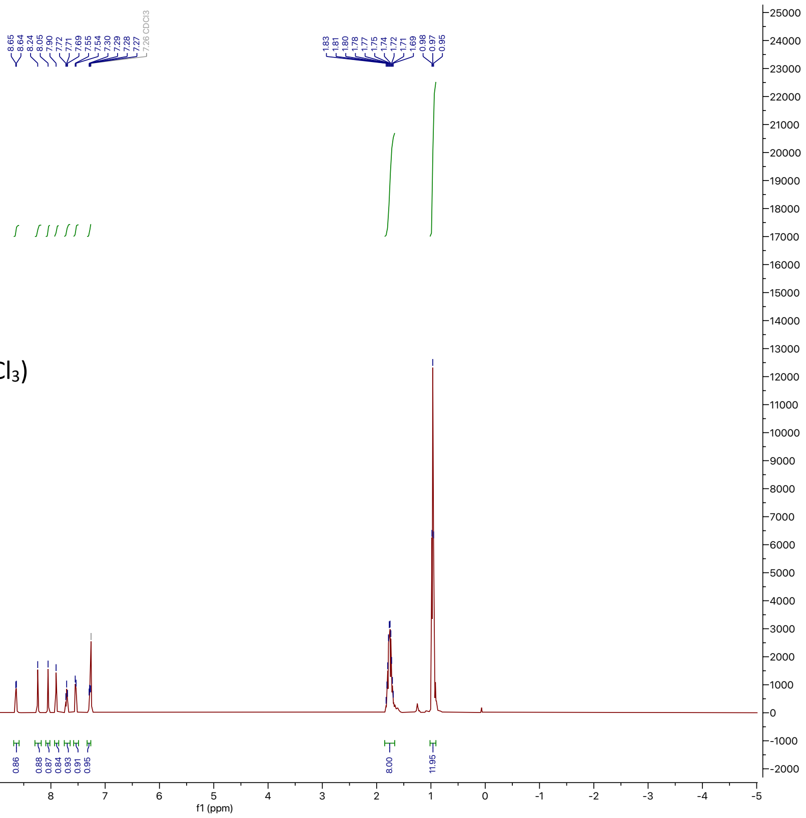

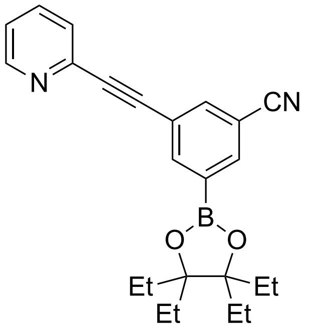

S24

^{13}C NMR (126 MHz, $CDCl_3$)

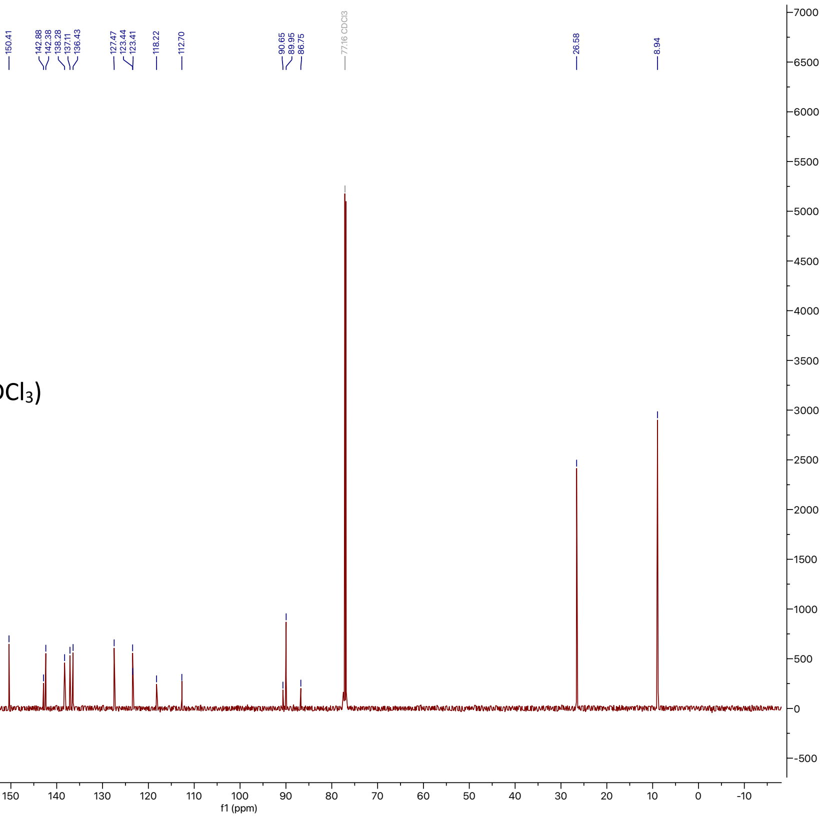

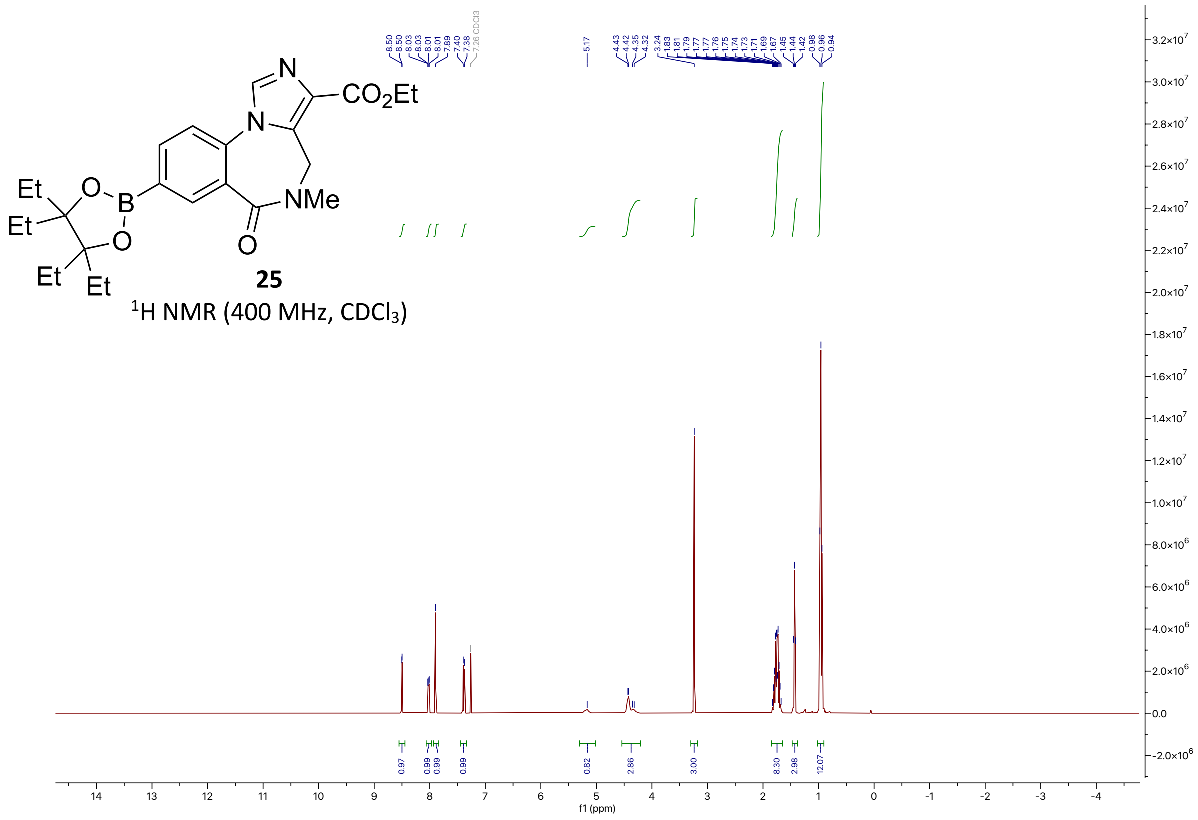

25

^{1}H NMR (400 MHz, $CDCl_3$)

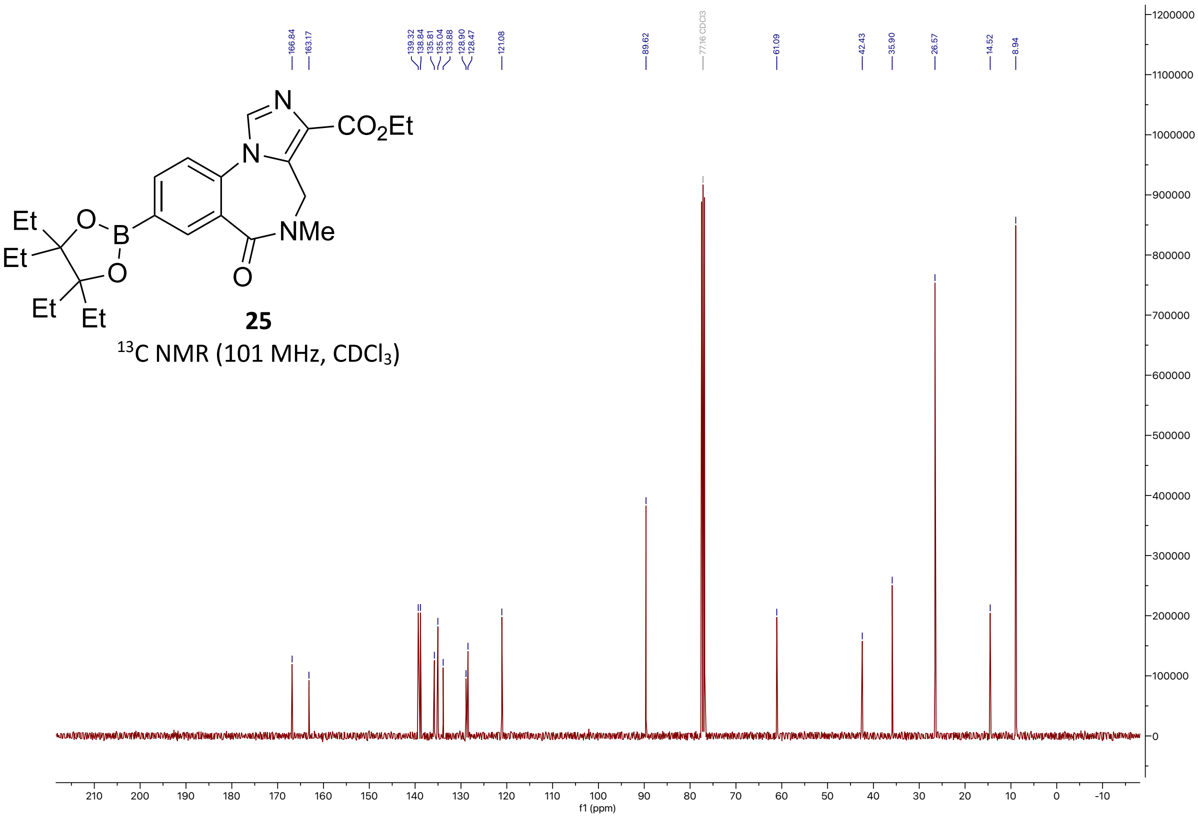

25

^{13}C NMR (101 MHz, $CDCl_3$)

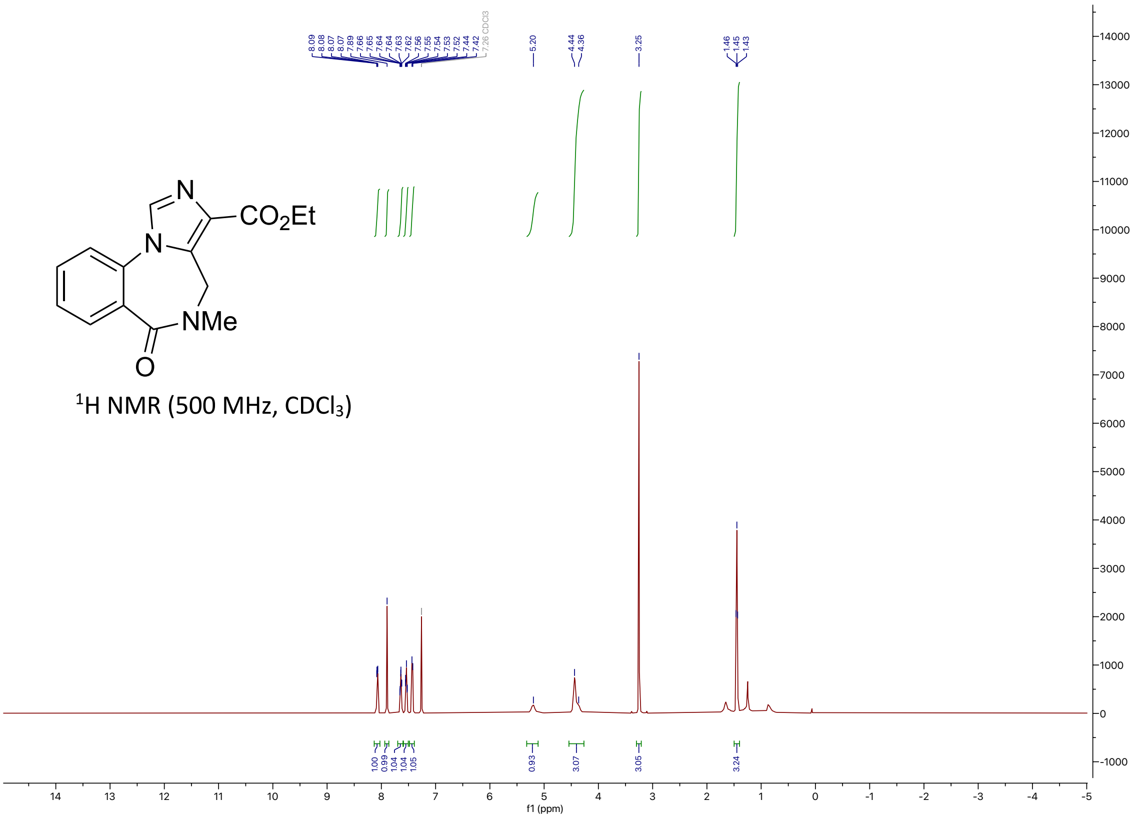
8.09
8.08
8.07
8.07
7.89
7.66
7.65
7.64
7.64
7.63
7.62
7.56
7.55
7.54
7.53
7.52
7.44
7.42
7.26 CDCl3
5.20
4.44
4.36
3.25
1.46
1.45
1.43
N
CO_2Et
N
NMe
O
^1H NMR (500 MHz, $CDCl_3$)
1.00
0.99
1.04
1.04
1.05
0.93
3.07
3.05
3.24
f1 (ppm)

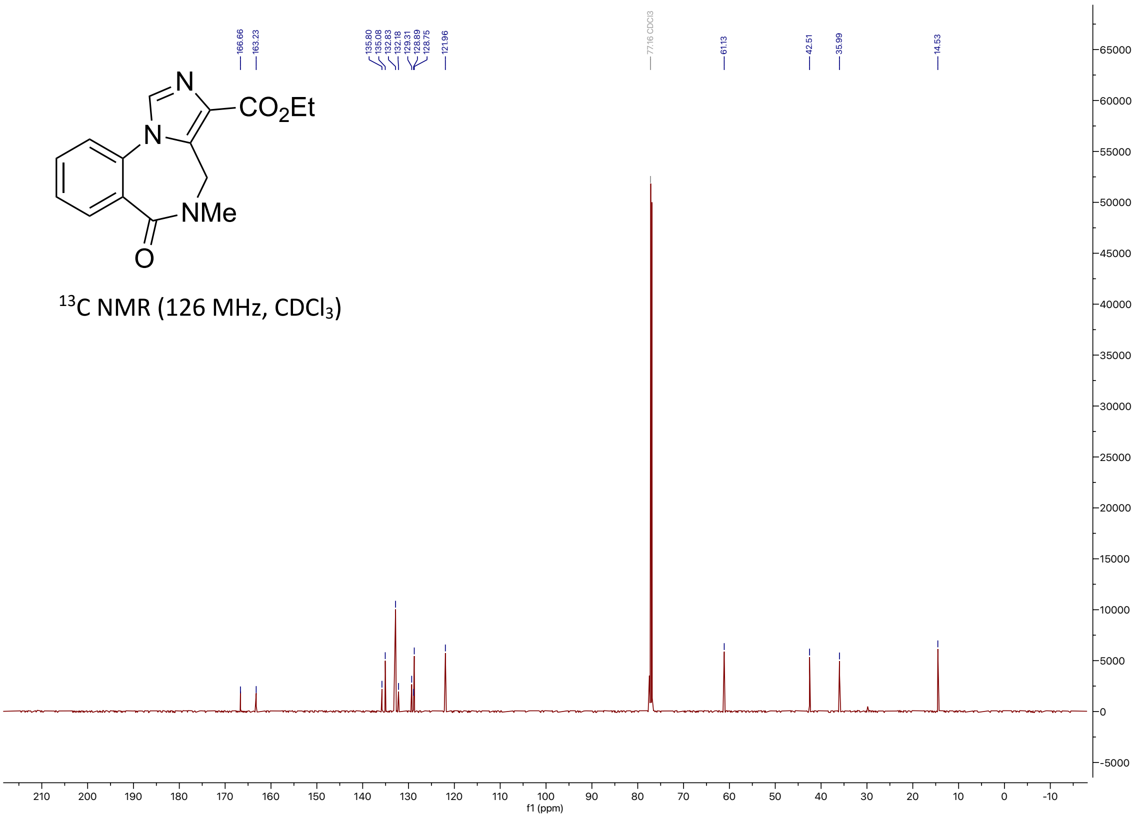
166.66
163.23
135.80
135.08
132.83
132.18
129.31
128.89
128.75
121.96
77.16 CDCl3
61.13
42.51
35.99
14.53
N
CO_2Et
N
NMe
O
^{13}C NMR (126 MHz, $CDCl_3$)
f1 (ppm)

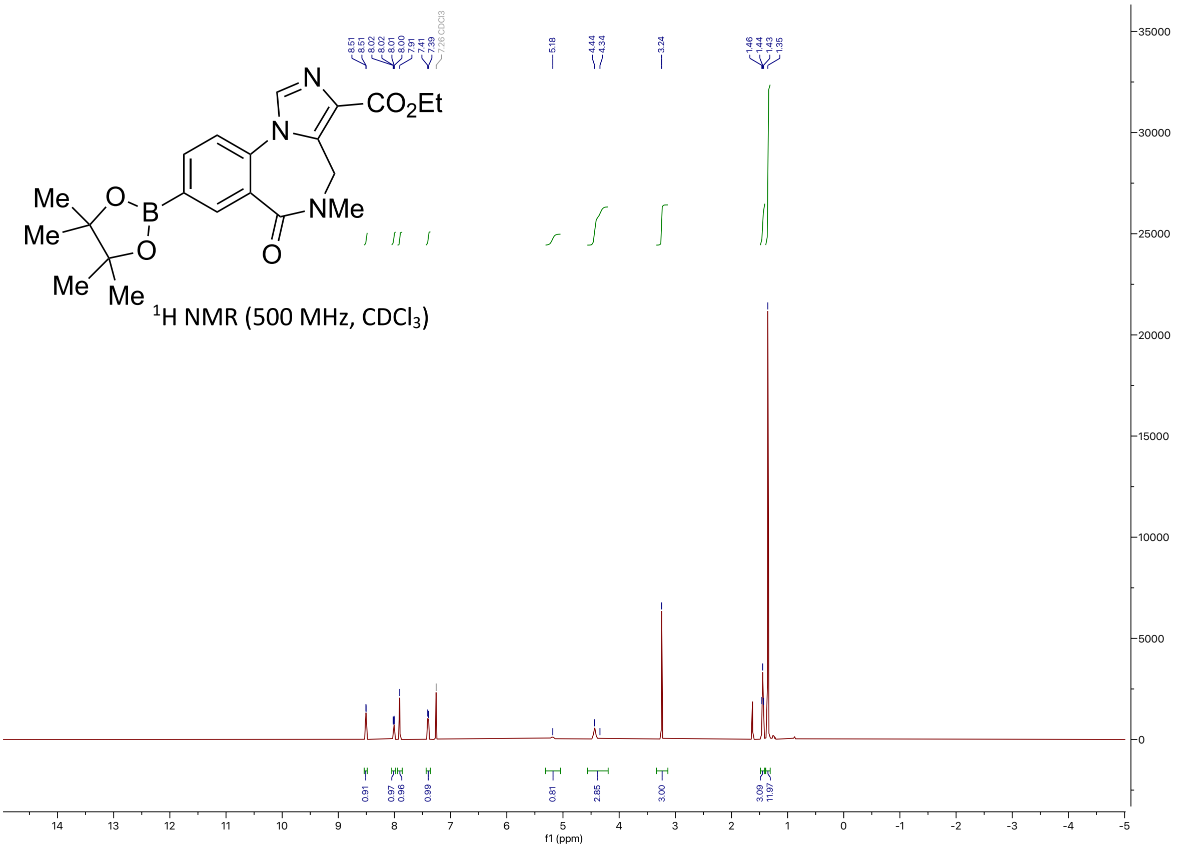
CO2Et
N
NMe
O
Me
B
1H NMR (500 MHz, CDCl3)
f1 (ppm)

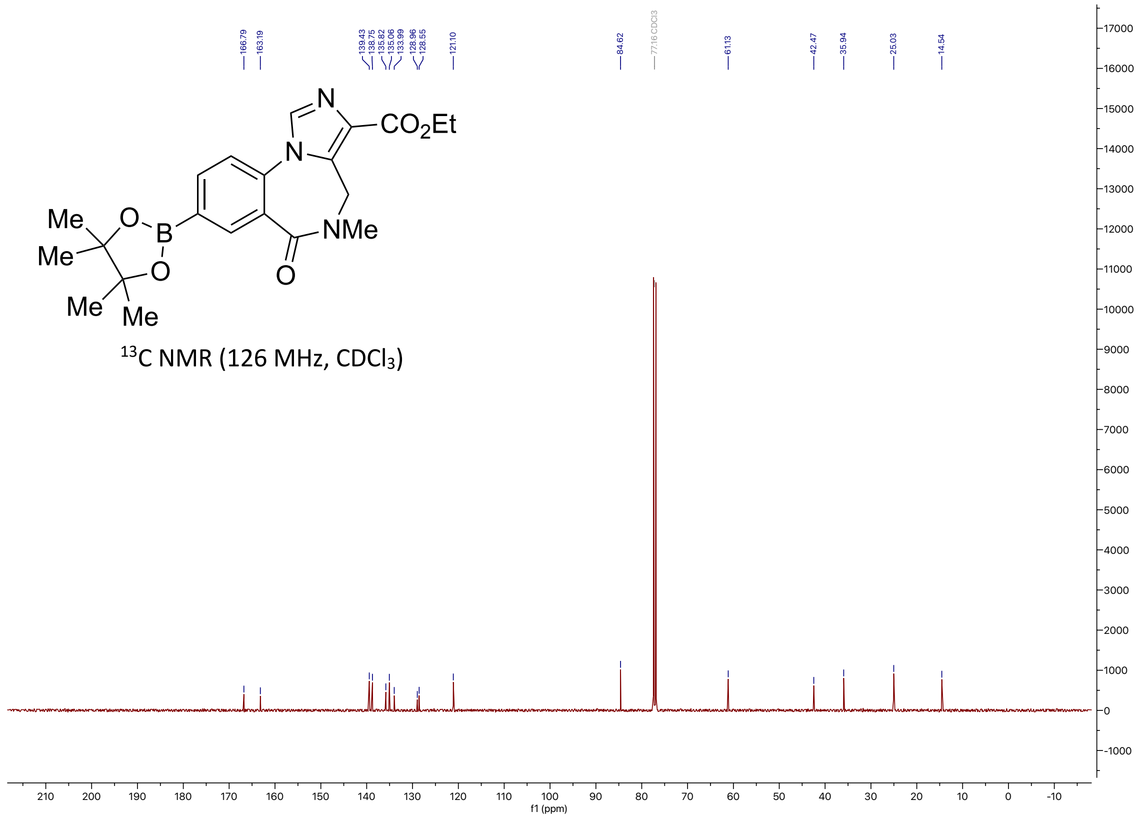
CO2Et
N
NMe
O
Me
B
13C NMR (126 MHz, CDCl3)
f1 (ppm)
